# Metal-Free Synthesis of 2-Substituted Quinazolines *via* Green Oxidation of *o*-Aminobenzylamines: Practical Construction of *N*-Containing Heterocycles Based on a Salicylic Acid-Catalyzed Oxidation System

**DOI:** 10.3389/fchem.2021.822841

**Published:** 2022-02-23

**Authors:** Yuki Yamamoto, Chihiro Yamakawa, Riku Nishimura, Chun-Ping Dong, Shintaro Kodama, Akihiro Nomoto, Michio Ueshima, Akiya Ogawa

**Affiliations:** Department of Applied Chemistry, Graduate School of Engineering, Osaka Prefecture University, Osaka, Japan

**Keywords:** quinazolines, amine oxidation, organocatalyst, one-pot reaction, *N*-heterocycles

## Abstract

Conventional quinazoline synthesis methods involve a highly multistep reaction, and often require excess amounts of substrate to control the product selectivity, leading to significant resource wastage. Hence, in this study, from the viewpoint of green chemistry, we developed a novel metal-free synthetic method for 2-substituted quinazoline derivatives by the 4,6-dihydroxysalicylic acid-catalyzed oxidative condensation of *o*-aminobenzylamines and benzylamines using atmospheric oxygen. In this system, the use of a catalytic amount of BF_3_‧Et_2_O (10 mol%) as a Lewis acid successfully led to the efficient oxidative condensation and intramolecular cyclization of these amines, followed by aromatization to afford the corresponding 2-arylquinazolines in up to 81% yield with excellent atom economy and environmental factor. Furthermore, to expand this green oxidation method to gram-scale synthesis, we investigated the development of an oxidation process using salicylic acid itself as an organocatalyst, and established a method for the practical green synthesis of a series of nitrogen-containing heterocycles. We expect that the findings will contribute to the development of practical synthesis methods for pharmaceutical manufacturing and industrial applications, along with further advancements in green chemistry.

## Introduction

In recent years, with advancements in pharmaceuticals and functional materials, the demand for a higher purity of the basic molecules constituting these materials has increased (Kündig, 2006; Ojima, 2017; Blakemore et al., 2018; Campos et al., 2019; Garcia-Martinez, 2021). Further, to mitigate the environmental impact of manufacturing processes, it is essential to develop resource-recyclable and highly atom-economical synthetic methods (Horvάth and Anastas, 2007; Sheldon, 2012; Hayashi, 2016; Horvάth, 2018). In this context, we recently succeeded in constructing an environmentally friendly metal-free oxidation catalyst system using oxygen (or air) at ambient pressure as an oxidant. Briefly, using 4,6-dihydroxysalicylic acid as an organocatalyst, the oxygen oxidation of amines to imines was achieved under mild conditions (Dong et al., 2016, eq 1). In this reaction, the catalyst can be recycled and used by supporting this salicylic acid derivative on silica gel. In addition, unstable imines can be easily prepared and used directly for the one-pot synthesis of various functional molecules, thus providing a series of innovative catalytic oxidation processes. This metal-free imine synthesis method not only enables the one-pot synthesis of important heterocyclic compounds, but also the highly selective one-pot reactions (e.g., the Ugi reaction) of multicomponent linkages (Dong, et al., 2017; Kumazawa, et al., 2018; Dong et al., 2019; Yamamoto et al., 2021). To further elucidate the versatility of this metal-free imine synthesis method, we attempted one-pot synthesis for reactions that are typically multistep, and succeeded in the metal-free synthesis of quinazoline derivatives (Wang and Gao, 2013; Faisal and Saeed, 2021), which are one of the heterocycles forming the basis of pharmaceuticals, agrochemicals, and functional materials (eq 2).


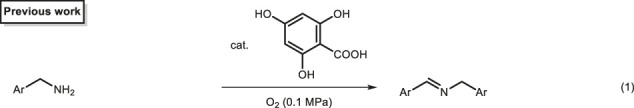



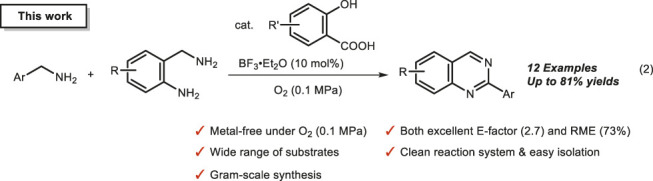


**SCHEME 1:**
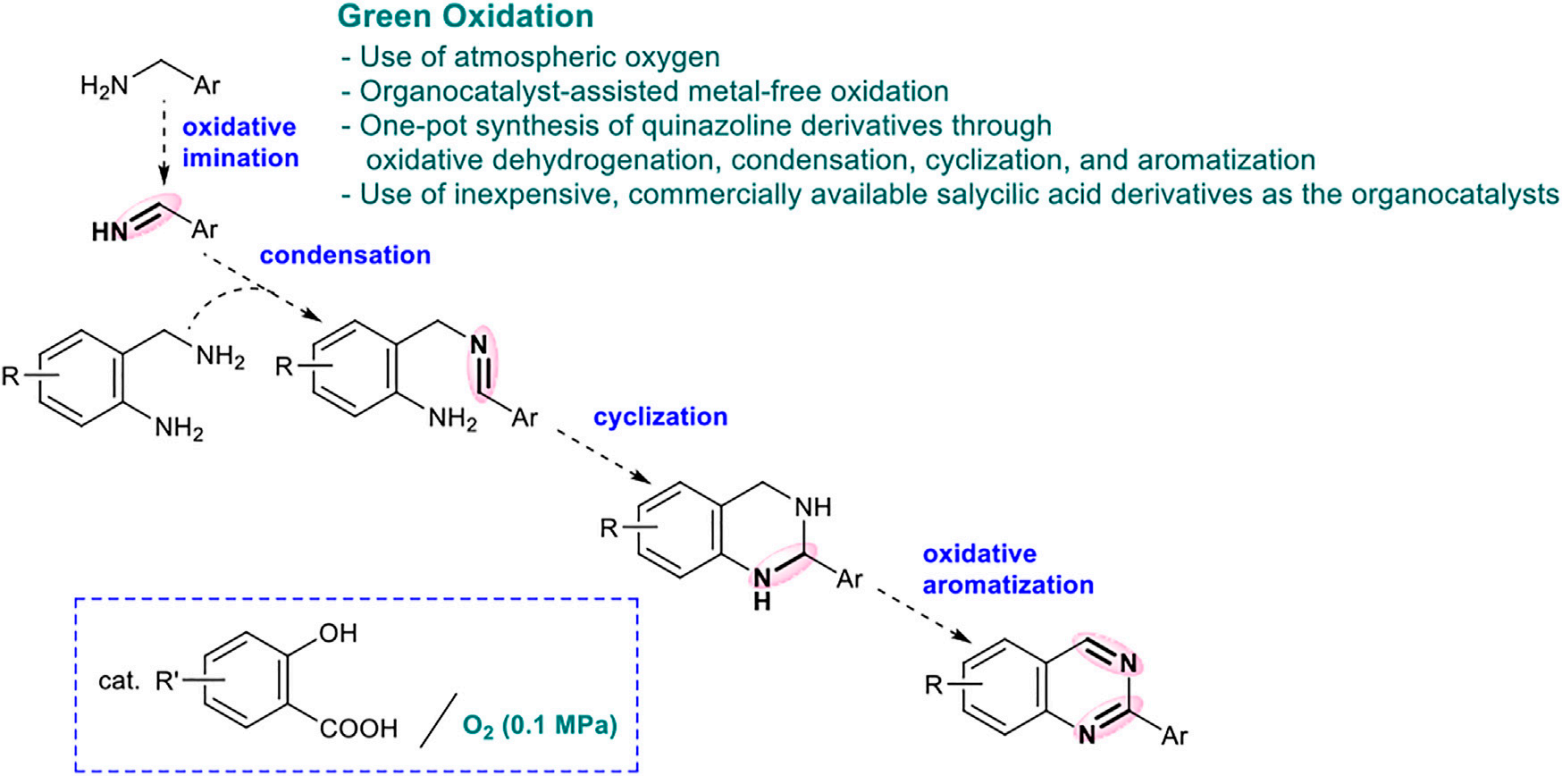
Quinazoline Synthesis *via* the Green Oxidation of *o*-Aminobenzylamine and Benzylamine.

This quinazoline synthesis method is a highly multistep reaction, comprising four reactions: 1) oxidative imine synthesis, 2) intermolecular condensation, 3) intramolecular cyclization, and 4) aromatization, using *o*-aminobenzylamine and benzylamine as the starting materials (Scheme 1). Since many byproducts could be generated from this multistep synthesis of quinazolines, several previous studies used an excess amount of benzylamine to selectively obtain the desired products. From the viewpoint of green chemistry, the development an eco-friendly synthesis method for quinazolines with excellent environmental factor (E-factor (%) = kg waste/kg product) and reaction mass efficiency (RME (%) = kg product/kg all reactants × 100) remains challenging (see the Supplementary Information, Han et al., 2011; Saha et al., 2017; Yamaguchi et al., 2016; Yamaguchi et al., 2017; Gujjarappa et al., 2018.). The availability of quinazolines for large-scale synthesis under metal-free conditions is also an important factor in pharmaceutical and industrial chemistry; however, previous methods achieved synthesis only up to the 1 mmol scale (Tiwari and Bhanage, 2016; Gopalaiah, et al., 2017; Deshmukh and Bhanage, 2018). Thus, it is imperative to develop a practical synthesis method that can be implemented on a larger scale and offers excellent E-factor and RME.

## Materials and Methods

### General Information

Unless otherwise stated, all starting materials were purchased from commercial sources and used without further purification. All solvents were distilled before use. Compound **1b** was prepared according to the previously reported procedure (Amberchan et al., 2021). ^1^H NMR spectra were recorded in CDCl_3_ using the JEOL JNM-ECX400 (400 MHz) FT NMR, JEOL JNM-ECS400 (400 MHz) FT NMR, and the Bruker BioSpin Ascend 400 spectrometer (400 MHz) with Me_4_Si as the internal standard. ^13^C{^1^H} NMR spectra were recorded in CDCl_3_ using the JEOL JNM-ECX400 (100 MHz) FT NMR, JEOL JNM-ECS400 (100 MHz) FT NMR, the Bruker BioSpin Ascend 400 spectrometer (100 MHz).

### General Procedure for the Synthesis of 2-Substituted Quinazolines *via* the Oxidative Coupling of Two Kinds of Benzylamines

2-Aminobenzylamine **1** (3.0 mmol), benzylamine **2** (3.0 mmol), 2,4,6-trihydroxybenzoic acid monohydrate (5 mol%), BF_3_‧Et_2_O (10 mol%), and DMSO (1.0 ml) were added to a 10 ml two-neck flask equipped with an O_2_ balloon at 25°C and stirred at 90°C in an oil bath under an O_2_ atmosphere for 48 h. After the reaction, the resulting mixture was purified by column chromatography using activated alumina as the column filler (eluent: AcOMe/*iso*-hexane) to afford product **3**.

*2-Phenylquinazoline* (**3aa**) (CAS no. 25855-20-3) (Yamaguchi et al., 2016). Yellow solid, 392.9 mg, 64% yield; ^1^H NMR (400 MHz, CDCl_3_): δ 9.47 (s, 1H), 8.62 (dd, *J* = 7.8, 1.8 Hz, 2H), 8.09 (d, *J* = 8.7 Hz, 1H), 7.93–7.89 (m, 2H), 7.61 (t, *J* = 7.6 Hz, 1H), 7.57–7.49 (m, 3H); ^13^C{^1^H} NMR (100 MHz, CDCl_3_): δ 161.2, 160.6, 150.9, 138.1, 134.2, 130.7, 128.74, 128.66, 127.4, 127.2, 123.7 (one carbon was overlapped to others).

*2-*(*2-Methoxyphenyl*)*quinazoline* (**3ab**) (CAS no. 27131-17-5) (Gopalaiah et al., 2017). Yellow solid, 261.0 mg, 37% yield; ^1^H NMR (400 MHz, CDCl_3_): δ 9.47 (s, 1H), 8.09 (d, *J* = 8.2 Hz, 1H), 7.88–7.83 (m, 2H), 7.80 (dd, *J* = 7.8, 1.8 Hz, 1H), 7.56 (t, *J* = 7.6 Hz, 1H), 7.44–7.40 (m, 1H), 7.09 (t, *J* = 7.3 Hz, 1H), 7.03 (d, *J* = 8.2 Hz, 1H), 3.85 (s, 3H); ^13^C{^1^H} NMR (100 MHz, CDCl_3_): δ 162.5, 160.1, 157.8, 150.7, 134.2, 131.9, 131.0, 129.0, 128.6, 127.6, 127.1, 123.2, 120.9, 112.0, 56.1.

*2-*(*3-Methoxyphenyl*)*quinazoline* (**3ac**) (CAS no. 1208259-21-5) (Wendlandt and Stahl, 2014). Yellow solid, 513.8 mg, 72% yield; ^1^H NMR (400 MHz, CDCl_3_): δ 9.30 (s, 1H), 8.24–8.22 (m, 1H), 8.19–8.18 (m, 1H), 7.99 (d, *J* = 8.2 Hz, 1H), 7.76–7.70 (m, 2H), 7.44–7.38 (m, 2H), 7.04–7.01 (m, 1H), 3.87 (s, 3H); ^13^C{^1^H} NMR (100 MHz, CDCl_3_): δ 160.9, 160.5, 160.1, 150.8, 139.6, 134.2, 129.7, 128.8, 127.4, 127.2, 123.7, 121.2, 117.4, 113.0, 55.5.

*2-*(*4-Methoxyphenyl*)*quinazoline* (**3ad**) (CAS no. 67205-04-3) (Han et al., 2012). Yellow solid, 308.5 mg, 44% yield; ^1^H NMR (400 MHz, CDCl_3_): δ 9.42 (s, 1H), 8.58 (dd, *J* = 6.8, 1.8 Hz, 2H), 8.04 (d, *J* = 8.6 Hz, 1H), 7.91–7.86 (m, 2H), 7.59–7.55 (m, 1H), 7.05 (dd, *J* = 6.8, 2.3 Hz, 2H), 3.90 (s, 3H); ^13^C{^1^H} NMR (100 MHz, CDCl_3_): δ 161.9, 160.9, 160.5, 150.9, 134.1, 130.8, 130.3, 128.5, 127.2, 126.9, 123.4, 114.1, 55.5.

*2-*(*2-Methylphenyl*)*quinazoline* (**3ae**) (CAS no. 1208259-15-7) (Ma et al., 2017). Yellow solid, 322.1 mg, 49% yield; ^1^H NMR (400 MHz, CDCl_3_): δ 9.46 (s, 1H), 8.07 (d, *J* = 8.6 Hz, 1H), 7.94–7.86 (m, 3H), 7.59 (t, *J* = 7.5 Hz, 1H), 7.36–7.32 (m, 3H), 2.61 (s, 3H); ^13^C{^1^H} NMR (100 MHz, CDCl_3_): δ 164.1, 160.2, 150.5, 138.7, 137.5, 134.2, 131.4, 130.8, 129.4, 128.6, 127.6, 127.2, 126.1, 123.0, 21.2.

*2-*(*3-Methylphenyl*)*quinazoline* (**3af**) (CAS no. 1208259-19-1) (Chakraborty et al., 2019). Yellow solid, 398.2 mg, 60% yield; ^1^H NMR (400 MHz, CDCl_3_): δ 9.40 (s, 1H), 8.42 (m, 2H), 8.05 (d, *J* = 8.8 Hz, 1H), 7.86–7.81 (m, 2H), 7.55–7.51 (m, 1H), 7.41 (t, *J* = 7.6 Hz, 1H), 7.30 (d, *J* = 7.5 Hz, 1H), 2.47 (s, 3H); ^13^C{^1^H} NMR (100 MHz, CDCl_3_): δ 161.2, 160.4, 150.8, 138.3, 138.0, 134.1, 131.5, 129.2, 128.6, 127.2, 127.1, 123.6, 21.6.

*2-*(*4-Methylphenyl*)*quinazoline* (**3ag**) (CAS no. 80089-59-4) (Ma et al., 2017). Yellow solid, 326.5 mg, 49% yield; ^1^H NMR (400 MHz, CDCl_3_): δ 9.44 (s, 1H), 8.51 (d, *J* = 8.2 Hz, 2H), 8.06 (d, *J* = 8.6 Hz, 1H), 7.91–7.86 (m, 2H), 7.60–7.56 (m, 1H), 7.34 (d, *J* = 8.2 Hz, 2H), 2.44 (s, 3H); ^13^C{^1^H} NMR (100 MHz, CDCl_3_): δ 161.2, 160.5, 150.9, 141.0, 135.4, 134.1, 129.5, 128.6, 128.2, 127.2, 127.1, 123.6, 21.6.

*2-*(*4-tert-Butylphenyl*)*quinazoline* (**3ah**) (CAS no. 1259300-25-8) (Yamaguchi et al., 2016). Yellow solid, 608.7 mg, 77% yield; ^1^H NMR (400 MHz, CDCl_3_): δ 9.33 (s, 1H), 8.56 (dd, *J* = 8.2, 1.8 Hz, 2H), 8.01 (d, *J* = 8.2 Hz, 1H), 7.77–7.73 (m, 2H), 7.54 (dd, *J* = 8.6, 1.8 Hz, 2H), 7.43 (t, *J* = 7.5 Hz, 1H), 1.37 (s, 9H); ^13^C{^1^H} NMR (100 MHz, CDCl_3_): δ 161.1, 160.5, 154.0, 150.9, 135.5, 134.2, 134.0, 128.6, 127.2, 127.1, 125.7, 123.6, 35.0, 31.4.

*2-*(*3-Fluorophenyl*)*quinazoline* (**3aj**) (CAS no. 1596243-24-1) (Wan et al., 2019). Yellow solid, 404.6 mg, 60%; ^1^H NMR (400 MHz, CDCl_3_): δ 9.37 (s, 1H), 8.40–8.37 (m, 1H), 8.32–8.29 (m, 1H), 8.02 (d, *J* = 8.8 Hz, 1H), 7.86–7.82 (m, 2H), 7.56–7.53 (m, 1H), 7.48–7.42 (m, 1H), 7.19–7.14 (m, 1H); ^13^C{^1^H} NMR (100 MHz, CDCl_3_): δ 163.3 (d, *J*_C–F_ = 243.4 Hz), 160.5, 159.7 (d, *J*_C–F_ = 3.1 Hz), 150.6, 140.5 (d, *J*_C–F_ = 7.8 Hz), 134.2, 130.0 (d, *J*_C–F_ = 7.9 Hz), 128.7, 127.6, 127.1, 124.2 (d, *J*_C–F_ = 2.8 Hz), 123.7, 117.4 (d, *J*_C–F_ = 21.3 Hz), 115.4 (d, *J*_C–F_ = 23.1 Hz).

*2-*(*3-Chlorophenyl*)*quinazoline* (**3ak**) (CAS no. 1353000-31-3) (Wan et al., 2019). Yellow solid, 278.3 mg, 39%; ^1^H NMR (400 MHz, CDCl_3_): δ 9.45 (s, 1H), 8.63 (m, 1H), 8.52–8.49 (m, 1H), 8.09–8.07 (m, 1H), 7.93–7.89 (m, 1H), 7.64–7.60 (m, 1H), 7.48–7.43 (m, 2H); ^13^C{^1^H} NMR (100 MHz, CDCl_3_): δ 160.6, 159.7, 150.7, 139.9, 134.8, 134.3, 130.6, 129.9.128.70, 128.67, 127.7, 127.2, 126.7, 123.8.

*2-*(*4-Fluorophenyl*)*quinazoline* (**3al**) (CAS no. 1208259-07-7) (Gopalaiah et al., 2017). Yellow solid, 374.2 mg, 56%; ^1^H NMR (400 MHz, CDCl_3_): δ 9.36 (d, *J* = 0.6 Hz, 1H), 8.62–8.57 (m, 2H), 8.02–8.00 (m, 1H), 7.86–7.82 (m, 2H), 7.55–7.51 (m, 1H), 7.20–7.14 (m, 2H); ^13^C{^1^H} NMR (100 MHz, CDCl_3_): δ 164.7 (d, *J*_C–F_ = 248.6 Hz), 160.5, 160.0, 150.7, 134.2 (d, *J*_C–F_ = 2.8 Hz), 134.1, 130.7 (d, *J*_C–F_ = 8.6 Hz), 128.5, 127.2, 127.1, 123.5, 115.5 (d, *J*_C–F_ = 21.5 Hz).

*2-*(*4-Chlorophenyl*)*quinazoline* (**3am**) (CAS no. 80089-58-3) (Yamaguchi et al., 2016). Yellow solid, 491.3 mg, 68% yield; ^1^H NMR (400 MHz, CDCl_3_): δ 9.32 (s, 1H), 8.50 (d, *J* = 8.6 Hz, 2H), 7.98 (d, *J* = 8.6 Hz, 1H), 7.83–7.78 (m, 2H), 7.53–7.49 (m, 1H), 7.43 (d, *J* = 8.6 Hz, 2H); ^13^C{^1^H} NMR (100 MHz, CDCl_3_): δ 160.5, 159.9, 150.6, 136.8, 136.6, 134.3, 130.0, 128.8, 128.6, 127.5, 127.2, 123.6.

*2-*(*3,5-Difluorophenyl*)*quinazoline* (**3an**) (CAS no. 2242488-07-7) (Parua et al., 2018). Yellow solid, 497.9 mg, 69%; ^1^H NMR (400 MHz, CDCl_3_): δ 9.47 (s, 1H), 8.21–8.16 (m, 2H), 8.11–8.09 (m, 1H), 7.98–7.93 (m, 2H), 7.69–7.65 (m, 1H), 6.97–6.92 (m, 1H); ^13^C{^1^H} NMR (100 MHz, CDCl_3_): δ 164.5 (d, *J*_C–F_ = 12.3 Hz), 162.1 (d, *J*_C–F_ = 12.4 Hz), 160.6, 158.7 (dd, *J*_C–F_ = 3.8, 4.0 Hz), 150.5, 141.6 (dd, *J*_C–F_ = 9.6, 9.8 Hz), 134.4, 128.7, 127.9, 127.1, 123.9, 111.5–111.2 (m), 105.7 (dd, *J*_C–F_ = 25.8, 25.4 Hz).

*2-*(*3,4-Difluorophenyl*)*quinazoline* (**3ao**) (CAS no. 1642143-98-3) (Li et al., 2014). Yellow solid, 263.8 mg, 36%; ^1^H NMR (400 MHz, CDCl_3_): δ 9.42 (s, 1H), 8.49–8.44 (m, 1H), 8.41–8.38 (m, 1H), 8.06–8.04 (m, 1H), 7.93–7.89 (m, 2H), 7.64–7.61 (m, 1H), 7.32–7.25 (m, 1H); ^13^C{^1^H} NMR (100 MHz, CDCl_3_): δ 160.6, 159.0, 152.7 (dd, *J*_C–F_ = 165.8, 13.3 Hz), 150.6, 150.1 (dd, *J*_C–F_ = 160.9, 13.0 Hz), 135.3 (dd, *J*_C–F_ = 6.0, 4.4 Hz), 134.4, 128.6, 127.6, 127.2, 124.9 (dd, *J*_C–F_ = 6.7, 3.4 Hz), 123.6, 117.5 (dd, *J*_C–F_ = 34.9, 18.9 Hz).

*4-*(*Quinazolin-2-yl*)*benzonitrile* (**3ap**) (CAS no. 154221-01-9) (Li et al., 2014). Light yellow solid, 381.7 mg, 55%; ^1^H NMR (400 MHz, CDCl_3_): δ 9.48 (s, 1H), 8.75–8.73 (m, 2H), 8.12–8.09 (m, 1H), 7.98–7.93 (m, 2H), 7.82–7.79 (m, 2H), 7.70–7.66 (m, 1H); ^13^C{^1^H} NMR (100 MHz, CDCl_3_): δ 160.7, 159.1, 150.6, 142.1, 134.5, 132.4, 129.0, 128.8, 128.2, 127.2, 123.9, 118.9, 113.7.

*2-*(*4-Nitrophenyl*)*quinazoline* (**3aq**) (CAS no. 80089-57-2) (Saadati et al., 2018). Yellow solid, 375.5 mg, 50%; ^1^H NMR (400 MHz, CDCl_3_): δ 9.51 (s, 1H), 8.81 (m, 2H), 8.36 (m, 2H), 8.13 (m, 1H), 7.98 (m, 2H), 7.70 (m, 1H); ^13^C{^1^H} NMR (100 MHz, CDCl_3_): δ 160.7, 158.9, 150.6, 149.2, 143.9, 134.6, 129.4, 128.8, 128.3, 127.2, 123.9, 123.8.

*2-*[*4-*(*Trifluoromethyl*)*phenyl*]*quinazoline* (**3ar**) [CAS no. 1208259-10-2] (Ye et al., 2013). Yellow solid, 553.0 mg, 67% yield; ^1^H NMR (400 MHz, CDCl_3_): δ 9.39 (s, 1H), 8.69 (d, *J* = 8.2 Hz, 2H), 8.05 (d, *J* = 9.1 Hz, 1H), 7.90–7.86 (m, 2H), 7.74 (d, *J* = 8.2 Hz, 2H), 7.59 (m, 1H); ^13^C{^1^H} NMR (100 MHz, CDCl_3_): δ 160.6, 159.6, 150.7, 141.3, 134.4, 132.2 (m), 128.9, 128.8, 127.9, 127.2, 125.5 (m), 123.8, 123.0.

*2-*(*2-Thienyl*)*quinazoline* (**3as**) (CAS no. 154221-04-2) (Chen et al., 2013). Yellow solid, 461.0 mg, 72% yield; ^1^H NMR (400 MHz, CDCl_3_): δ 9.28 (s, 1H), 8.14–8.13 (m, 1H), 7.95 (d, *J* = 8.6 Hz, 1H), 7.82–7.78 (m, 2H), 7.50–7.46 (m, 2H), 7.18–7.15 (m, 1H); ^13^C{^1^H} NMR (100 MHz, CDCl_3_): δ 160.6, 157.9, 150.6, 144.0, 134.4, 130.1, 129.3, 128.5, 128.2, 127.3, 127.1, 123.4.

*2-*(*Pyridin-3-yl*)*quinazoline* (**3at**) (CAS no. 917224-71-6) (Chakraborty et al., 2019). Yellow solid. 418.1 mg, 67%; ^1^H NMR (400 MHz, CDCl_3_): δ 9.82 (dd, *J* = 2.2, 0.2 Hz, 1H), 9.46 (d, *J* = 0.7 Hz, 1H), 8.86 (dt, *J* = 8.0, 2.0 Hz, 1H), 8.74 (dd, *J* = 5.0, 1.7 Hz, 1H), 8.10–8.07 (m, 1H), 7.98–7.90 (m, 2H), 7.66–7.62 (m, 1H), 7.46–7.43 (m, 1H); ^13^C{^1^H} NMR (100 MHz, CDCl_3_): δ 160.7, 159.2, 151.3, 150.6, 150.3, 135.8, 134.4, 128.7, 127.8, 127.2, 123.8, 123.4.

*6-Bromo-2-phenylquinazoline* (**3ba**) (CAS no. 1004997-72-1) (Taylor et al., 2017). White solid, 190.3 mg, 22% yield; ^1^H NMR (400 MHz, CDCl_3_): δ 9.39 (s, 1H), 8.61–8.59 (m, 2H), 8.08–8.08 (m, 1H), 7.96–7.96 (m, 2H), 7.57–7.52 (m, 3H); ^13^C{^1^H} NMR (100 MHz, CDCl_3_): δ 161.4, 159.5, 149.5, 137.7, 137.7, 131.0, 130.5, 129.3, 128.8, 128.7, 124.6, 120.8.

### Gram-Scale Synthesis of 2-Phenylquinazoline 3aa Based on 4,6-Dihydroxysalicylic Acid-Catalyzed Oxidation System

2-Aminobenzylamine **1** (10 mmol), benzylamine derivative **2** (10 mmol), 2,4,6-trihydroxybenzoic acid monohydrate (5 mol%), BF_3_‧Et_2_O (10 mol%), and DMSO (2.5 ml) were added to a 20 ml two-neck flask equipped with an O_2_ balloon at 25°C and stirred at 90°C in an oil bath under an O_2_ atmosphere for 48 h. After the reaction, the resulting mixture was purified by column chromatography using activated alumina as the column filler (eluent: AcOMe/*iso*-hexane) to furnish product **3aa** in 50% isolated yield (yellow solid, 1.0 g).

### General Procedure for the Salicylic Acid-Catalyzed Oxidation of Benzylamines to Imines

Benzylamine derivative **2** (3.0 mmol), salicylic acid (5 mol%), 4A MS (100 mg), and toluene (1.5 ml) were added to a 10 ml two-neck flask equipped with an O_2_ balloon at 25°C and stirred at 90°C in an oil bath under an O_2_ atmosphere for 16 h. After filtration of the crude product with AcOMe using silica gel, distillation was conducted to afford pure imine **4**.

*N-*(*Phenylmethylene*)*benzenemethanamine* (**4a**) (CAS no. 780-25-6) (Dong et al., 2016). Yellow oil, 264.2 mg, 90% yield; ^1^H NMR (400 MHz, CDCl_3_): δ 8.35 (s, 1H), 7.78–7.75 (m, 2H), 7.39–7.38 (m, 3H), 7.33–7.32 (m, 4H), 7.27–7.23 (m, 1H), 4.80 (s, 2H); ^13^C{^1^H} NMR (100 MHz, CDCl_3_): δ 162.2, 139.5, 136.3, 130.9, 128.8, 128.7, 128.5, 128.1, 127.2, 65.2.

*2-Methoxy-N-*[(*2-methoxyphenyl*)*methylene*]-*benzenemethanamine* (**4b**) (CAS no. 161723-67-7) (Dong et al., 2016). Yellow oil, 286.2 mg, 75%; ^1^H NMR (400 MHz, CDCl_3_): δ 8.84 (s, 1H), 8.04 (dd, *J* = 7.5, 1.6 Hz, 1H), 7.37–7.29 (m, 2H), 7.24–7.19 (m, 1H), 6.98–6.90 (m, 2H), 6.86 (t, *J* = 8.8 Hz, 2H), 4.83 (s, 2H), 3.82 (s, 3H), 3.81 (s, 3H); ^13^C{^1^H} NMR (100 MHz, CDCl_3_) δ 158.9, 158.4, 157.2, 131.9, 129.2, 128.3, 128.1, 127.6, 125.0, 120.9, 120.6, 111.1, 110.3, 59.8, 55.6, 55.5.

*4-Methoxy-N*-[(*4*-*methoxyphenyl*)*methylene*]-*benzenemethanamine* (**4c**) (CAS no. 3261-60-7) (Dong et al., 2016). Yellow oil, 268.1 mg, 70% yield; ^1^H NMR (400 MHz, CDCl_3_): δ 8.27 (s, 1H), 7.70 (d, *J* = 8.6 Hz, 2H), 7.23 (d, *J* = 8.6 Hz, 2H), 6.91–6.87 (m, 4H), 4.70 (s, 2H), 3.79 (s, 3H), 3.76 (s, 3H); ^13^C{^1^H} NMR (100 MHz, CDCl_3_): δ 161.8, 161.1, 158.7, 131.8, 129.9, 129.3, 114.1, 114.0, 64.5, 55.44, 55.37.

*4-Methyl-N-*[(*4-methylphenyl*)*methylene*]-*benzenemethanamine* (**4d**) (CAS no. 71022-60-1) (Dong et al., 2016). Yellow solid, 317.2 mg, 80% yield; ^1^H NMR (400 MHz, CDCl_3_) δ 8.33 (s, 1H), 7.66 (d, *J* = 6.3 Hz, 2H), 7.21–7.14 (m, 6H), 4.76 (s, 2H), 2.37 (s, 3H), 2.33 (s, 3H); ^13^C{^1^H} NMR (100 MHz, CDCl_3_): δ 161.8, 141.1, 136.6, 136.5, 133.7, 129.4, 129.3, 128.3, 128.1, 64.9, 21.6, 21.2.

### General Procedure for the Synthesis of Benzimidazoles Catalyzed by Salicylic Acid Under Atmospheric Oxygen

Benzylamine derivative **2** (4.5 mmol), *o*-phenylenediamine **5** (3.0 mmol), salicylic acid (10 mol% based on **2**), 4A MS (100 mg), and toluene (1.0 ml) were added to a 10 ml two-neck flask equipped with an O_2_ balloon at 25°C and stirred at 70°C in an oil bath under an O_2_ atmosphere for 24 h. After the reaction, the resulting mixture was purified by silica-gel column chromatography (eluent: AcOMe/*iso*-hexane with 5% Et_3_N) to obtain benzimidazole **6** (the yield was based on **5**).

*2-Phenylbenzimidazole* (**6a**) (CAS no. 716-79-0) (Dong et al., 2016). Yellow solid, 455.0 mg, 78% yield; ^1^H NMR (400 MHz, CD_3_OD): δ 8.09 (d, *J* = 7.2 Hz, 2H), 7.60 (s, 2H), 7.53–7.45 (m, 3H), 7.27–7.23 (m, 2H); ^13^C{^1^H} NMR (100 MHz, DMSO-*d*_6_): δ 151.8, 140.1, 130.6, 130.4, 129.5, 128.6, 127.7, 126.9, 122.7, 117.9, 115.6.

*2-*(*2-Methoxyphenyl*)*benzimidazole* (**6b**) (CAS no. 6528-85-4) (Dong et al., 2016). Brown solid, 535.1 mg, 80% yield; ^1^H NMR (400 MHz, DMSO-*d*_6_): δ 12.15 (br, 1H), 8.35 (dd, *J* = 7.6, 1.8 Hz, 1H). 7.65 (m, 2H), 7.48 (m, 1H), 7.25–7.10 (m, 4H), 4.03 (s, 3H); ^13^C{^1^H} NMR (100 MHz, DMSO-*d*_6_): δ 157.2, 149.4, 143.2, 135.2, 131.7, 130.2, 122.5, 122.0, 121.3, 118.9, 118.5, 112.6, 112.4, 56.2.

*2-*(*3-Methoxyphenyl*)*benzimidazole* (**6c**) (CAS no. 36677-36-8) (Dong et al., 2016). Yellow solid, 498.0 mg, 74% yield; ^1^H NMR (400 MHz, CD_3_OD): δ 7.69–7.59 (m, 4H), 7.42 (t, *J* = 7.9 Hz, 1H), 7.27–7.23 (m, 2H), 7.06–7.03 (m, 1H), 3.88 (s, 3H); ^13^C{^1^H} NMR (100 MHz, CD_3_OD): δ 160.4, 151.9, 130.8, 129.9, 122.6, 118.6, 116.0, 111.5, 54.5.

*2-*(*4-Methoxyphenyl*)*benzimidazole* (**6d**) (CAS no. 2620-81-7) (Dong et al., 2016). Yellow solid, 520.1 mg, 78% yield; ^1^H NMR (400 MHz, CD_3_OD): δ 8.02–7.98 (m, 2H), 7.58–7.52 (m, 2H), 7.25–7.21 (m, 2H), 7.04–7.01 (m, 2H), 3.82 (s, 3H); ^13^C{^1^H} NMR (100 MHz, CD_3_OD): δ 161.5, 152.2, 128.0, 122.3, 122.0, 119.4, 116.4, 114.1, 54.5.

*2-*(*4-Methylphenyl*)*benzimidazole* (**6e**) (CAS no. 120-03-6) (Dong et al., 2016). Yellow solid, 466.6 mg, 75% yield; ^1^H NMR (400 MHz, CD_3_OD): δ 7.97 (d, *J* = 8.6 Hz, 2H), 7.59 (m, 2H), 7.35 (d, *J* = 8.2 Hz, 2H), 7.26–7.22 (m, 2H), 2.41 (s, 3H); ^13^C{^1^H} NMR (100 MHz, CD_3_OD): δ 152.2, 140.6, 129.4, 126.9, 126.4, 122.4, 114.6, 20.1.

### Salicylic Acid-Catalyzed Oxidative Synthesis of 2-Phenylbenzothiazole

Benzylamine **2a** (4.0 mmol), 2-aminothiophenol **7** (3.0 mmol), salicylic acid (10 mol% based on **2a**), 4A MS (100 mg), and *p*-xylene (2.0 ml) were added to a 10 ml two-neck flask equipped with an O_2_ balloon 25°C and stirred at 140°C in an oil bath under an O_2_ atmosphere for 24 h. After the reaction, the resulting mixture was purified by silica-gel column chromatography (eluent: AcOMe/*iso*-hexane) to obtain 2-phenylbenzothiazole **8** (the yield was based on **7**).

*2-Phenylbenzothiazole* **8**) (CAS no. 883-93-2) (Kumazawa et al., 2018). White solid, 293.1 mg, 63% yield; ^1^H NMR (400 MHz, CDCl_3_): δ 8.06–8.02 (m, 3H), 7.77 (d, *J* = 7.7 Hz, 1H), 7.43-7.38 (m, 4H), 7.28 (t, *J* = 7.5 Hz, 1H); ^13^C{^1^H} NMR (100 MHz, CDCl_3_): δ 168.2, 154.3, 135.2, 133.7, 131.1, 129.1, 127.7, 126.5, 125.3, 123.4, 121.8.

### Salicylic Acid-Catalyzed Oxidative Synthesis of 2,4,6-Triphenylpyridine

Benzylamine **2a** (1.5 mmol), acetophenone **9** (1.0 mmol), salicylic acid (3.3 mol%), BF_3_‧Et_2_O (6.7 mol%), and DMSO (0.1 ml) were added to a 10 ml two-neck flask, and stirred at 100°C for 18 h under open air. The crude product was purified by silica-gel column chromatography (eluent: AcOMe/*iso*-hexane) to furnish 2,4,6-triphenylpyridine **10**.

*2,4,6-Triphenylpyridine* (**10**) (CAS no. 580-35-8) (Dong et al., 2019). Yellow solid, 88.6 mg, 58% yield; ^1^H NMR (400 MHz, CDCl_3_): δ 8.19 (d, *J* = 6.9 Hz, 4H), 7.86 (s, 2H), 7.72–7.70 (m, 2H), 7.51–7.40 (m, 9H); ^13^C{^1^H} NMR (100 MHz, CDCl_3_): δ 158.5, 151.2, 140.6, 140.0, 130.14, 130.10, 130.0, 129.8, 128.21, 128.19, 118.1.

### Multi-Gram-Scale Synthesis of Imine 4a via the Salicylic Acid-Catalyzed Green Oxidation of Benzylamine 2a

Benzylamine **2a** (110 mmol), salicylic acid (10 mol%), and 4A MS (1 g) were added to a 30 ml two-neck flask equipped with an O_2_ balloon at 25°C and stirred at 90°C in an oil bath under an O_2_ atmosphere for 72 h. After filtration with AcOMe using silica gel, the crude product was purified by distillation to afford pure imine **4a** in 94% isolated yield (yellow oil, 10.1 g).

## Results and Discussion

Considering that the oxidation system used 4,6-dihydroxysalicylic acid, we first investigated the reaction of benzylamine **1a** (3.0 mmol) with *o*-aminobenzylamine **2a** in the presence of the organocatalyst (10 mol%). Heating the mixture at 90°C for 24 h in toluene (1.0 ml) under atmospheric oxygen successfully afforded 2-phenylquinazoline **3aa** in 48% yield (entry 1, Table 1). A further increase in the amount of **2a** to 6.0 mmol did not improve the yield of **3aa** (entry 2). Conversely, a reduction in the amount of solvent resulted in a low yield of **3aa**, which contained oligomers that were insoluble in the solvent (entries 3 and 4). The addition of 15 mol% of the catalyst and increase in the reaction temperature to 110°C did not improve the yield of **3aa** (entries 5 and 6). Interestingly, the addition of a catalytic amount of BF_3_‧Et_2_O (10 mol%) under the same conditions as in entry 1 accelerated the formation of **3aa**, which was obtained in 56% yield. Furthermore, when the reaction solvent was optimized in the presence of BF_3_‧Et_2_O (10 mol%), DMSO was found to be the best solvent (entries 7–11).

**TABLE 1:** Optimization of reaction conditions for the synthesis of 2-phenylquinazoline **3aa**.
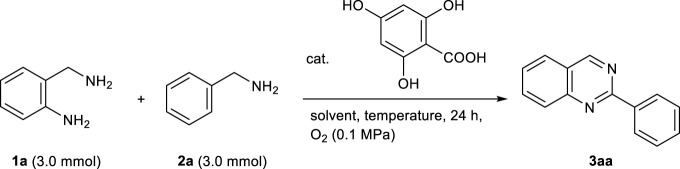

Entry	Solvent (ml)	Temp. (°C)	Cat. (mol%)	Time (h)	Additive (mol%)	Yield 3aa (%)*^a^*
1	Toluene (1.0)	90	10	24	—	48
2*^b^*	Toluene (1.0)	90	10	24	—	41
3	Toluene (0.5)	90	10	24	—	30
4	Neat	90	10	24	—	11
5	Toluene (1.0)	90	15	24	—	21
6	Toluene (1.0)	110	10	24	—	42
7	Toluene (1.0)	90	10	24	BF_3_‧Et_2_O (10)	56
8	Toluene (1.0)	70	10	24	BF_3_‧Et_2_O (10)	23
9	DMSO (1.0)	90	10	24	BF_3_‧Et_2_O (10)	63
10	DMF (1.0)	90	10	48	BF_3_‧Et_2_O (10)	30
11	CH_3_CN (1.0)	Reflux	10	48	BF_3_‧Et_2_O (10)	25
12	DMSO (1.0)	90	15	24	BF_3_‧Et_2_O (10)	58
13	DMSO (0.5)	90	10	24	BF_3_‧Et_2_O (10)	55
14	DMSO (1.0)	90	10	48	BF_3_‧Et_2_O (10)	71
15*^c^*	DMSO (1.0)	90	10	48	BF_3_‧Et_2_O (10)	42
16	DMSO (1.0)	90	10	48	BF_3_‧Et_2_O (30)	58
17	DMSO (1.0)	90	5	48	BF_3_‧Et_2_O (10)	81 (64)
18	DMSO (1.0)	90	1	48	BF_3_‧Et_2_O (10)	54
19	DMSO (1.0)	90	5	48	—	44
20	DMSO (1.0)	90	—	48	BF_3_‧Et_2_O (10)	14
21*^d^*	DMSO (1.0)	90	10	48	BF_3_‧Et_2_O (10)	Trace

a Yields were determined by ^1^H NMR spectroscopy (isolated yield).

b **2a** (6.0 mmol) was used.

c 4A MS (100 mg) was added as an additive.

d Under N_2_ atmosphere.

A detailed study of the reaction conditions based on entry 9 showed that **3aa** was successfully obtained in 81% yield by loading 4,6-dihydroxysalicylic acid (5 mol%) and BF_3_‧Et_2_O (10 mol%), and extending the reaction time to 48 h (entries 12–18). This conversion proceeded even without the catalytic amount of BF_3_‧Et_2_O, giving **3aa** in 44% yield (entry 19). Therefore, the catalytic amount of BF_3_‧Et_2_O may have an accelerating effect on the reaction. In the absence of the organocatalyst or under a N_2_ atmosphere, the yield of **3aa** significantly decreased (entries 20 and 21). These results strongly suggest that the organocatalytic oxidation of benzylamines using 4,6-dihydroxysalicylic acid as the catalyst is one of the key steps in this oxidative cyclization reaction. Notably, this quinazoline synthesis under optimal conditions (entry 17) exhibits excellent E-factor of 2.7 and RME (= 73%).

Using the optimal conditions (Table 1, entry 17), we then evaluated the substrate scope of the metal-free synthesis of 2-substituted quinazolines (Table 2). Various benzylamine derivatives such as *m*-methoxy, *p*-methoxy, *o*-methyl, *m*-methyl, *p*-methyl, *p*-*tert*-butyl, *m*-fluoro, *m*-chloro, *p*-fluoro, *p*-chloro, *p*-cyano, *p*-nitro, and *p*-trifluoromethyl-substituted benzylamines (**2c**–**2h**, **2j**–**2m**, and **2p**–**2r**) were examined, and the corresponding quinazoline derivatives (**3ac**–**3ah**, **3aj**–**3am**, and **3ap**–**3ar**) were obtained in moderate to good yields. When *o*-methoxy- and *o*-bromobenzylamine (**2b** and **2i**) were used as substrates, the yields of **3ab** and **3ai** were lower (37 and 17% yields, respectively) due to steric hindrance. This method was also applicable to fluorine-disubstituted benzylamines (**2n** and **2o**), and the corresponding quinazoline derivatives were obtained in moderate to good yields, respectively (**3an** and **3ao**). The use of 2-thiophenemethylamine (**2s**) and 3-(aminomethyl)pyridine (**2t**) were also examined, and product **3ak** and **3at** were obtained in 72 and 67% yields, respectively. *o*-Aminobenzylamine derivatives **1b** could also be used in the reaction, and quinazoline **3ba** was obtained in 44% yield. The reaction of *o*-aminobenzylamine **1a** with 1-hexylamine could not afford the corresponding product in sufficient yield, due to the low conversion of 1-hexylamine to the corresponding imine under the reaction condition. The quinazoline synthesis could also be conducted on a Gram scale, where **3aa** was isolated in 50% yield (1.0 g). Note that this quinazoline synthesis was carried out using salicylic acid itself as an organocatalyst, and **3aa** was obtained in 48% yield by prolonging the reaction to 5 days.

**TABLE 2:** Reaction scope for the metal-free/oxidative synthesis of 2-substituted quinazolines.
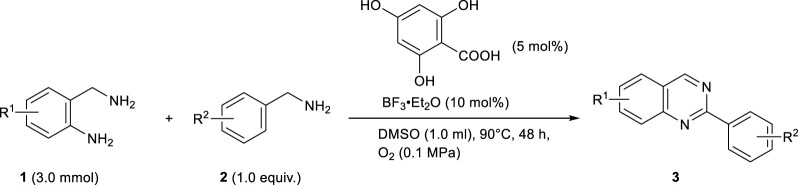

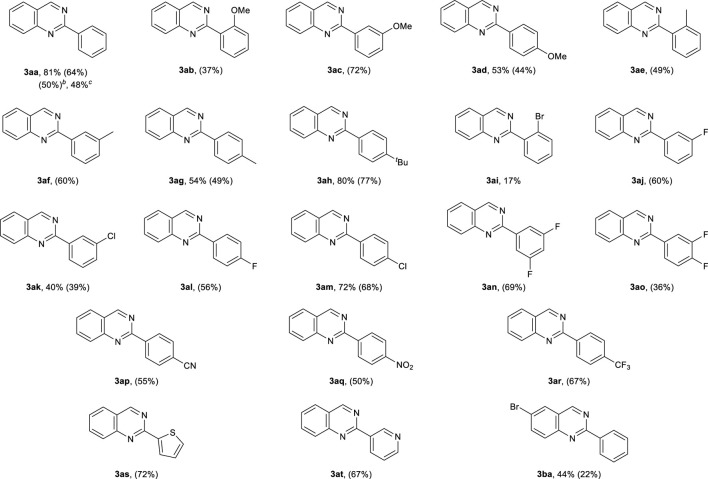

a Yields were determined by ^1^H NMR spectroscopy (isolated yields).

b Reaction conditions: **1a** (10.0 mmol), **2a** (1.0 equiv.), 4,6-dihydroxysalicylic acid (5 mol%), BF_3_‧Et_2_O (10 mol%), DMSO (2.5 ml), 90°C, 48 h, O_2_ (0.1 MPa). ^*c*^Reaction conditions: **1a** (3.0 mmol), **2a** (3.0 mmol), salicylic acid (10 mol%), 4A MS (100 mg), DMSO (1.0 ml), 90°C, 5 days, O_2_ (0.1 MPa).

To gain insights into the reaction mechanism for the oxidative formation of 2-arylquinazolines from two kinds of benzylamines, several control experiments were conducted. When imine **4a** (3.0 mmol), instead of **2a**, was allowed to react with **1a** (3.0 mmol) under the optimal conditions for quinazoline synthesis, the desired product **3aa** was obtained in 13% yield (eq 3). This indicates that imine **4a** might not be an important intermediate in this system. In addition, when the oxidation of benzylamine **1a** or 2-aminobenzylamine **2a** was conducted independently in the presence of 4,6-dihydroxysalicylic acid (5 mol%) and BF_3_‧Et_2_O (10 mol%) in DMSO, the resulting imines **4a** and **4b** were obtained in 14 and 9% yields, respectively (eq 4). Thus, **1a** and **2a** could be oxidized under the reaction conditions, and active imine species might be initially formed.


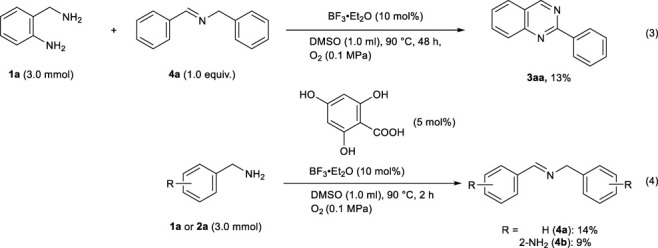


**SCHEME 2:**
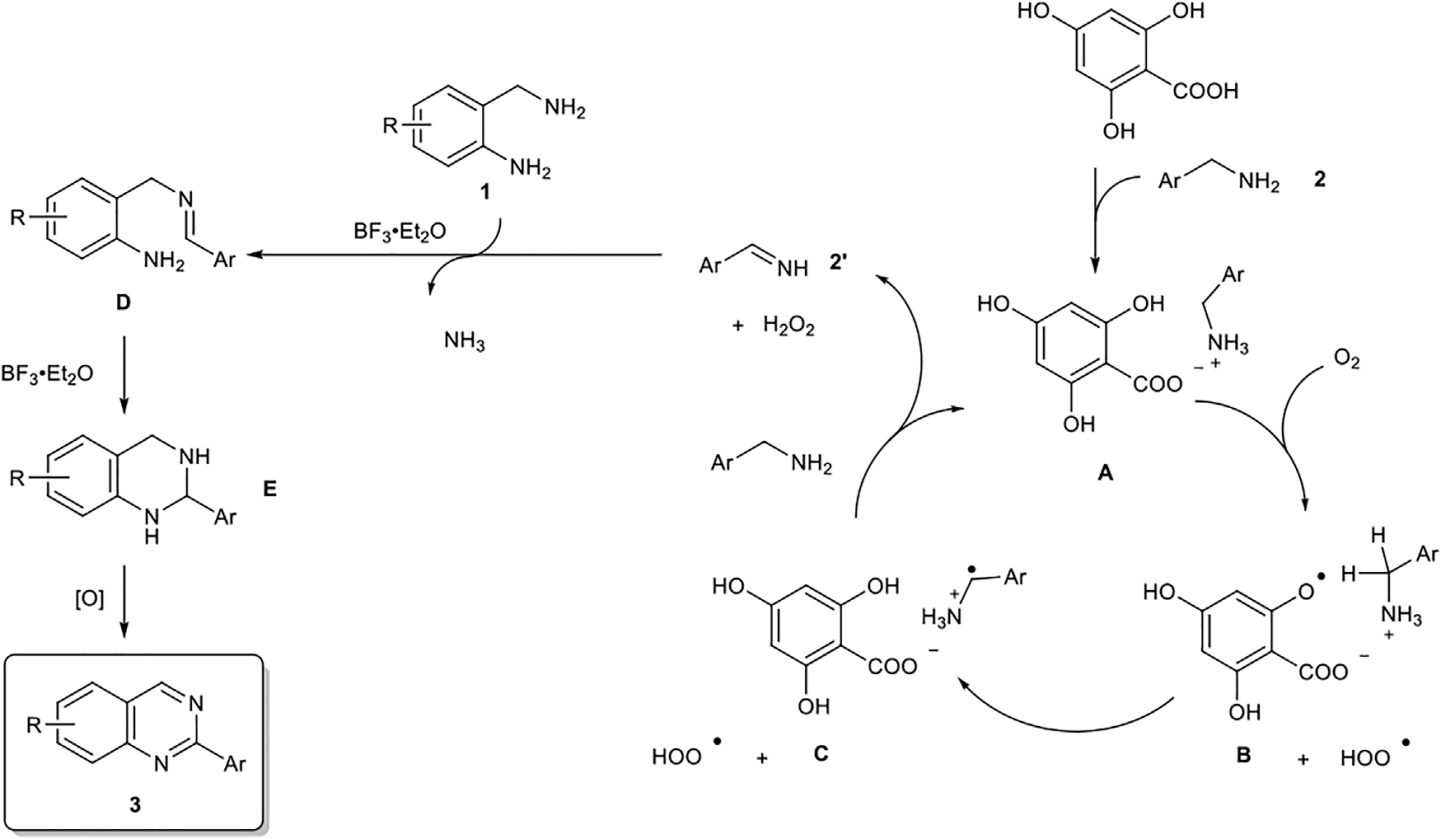
Proposed Reaction Pathways for the Organocatalytic Oxidative Formation of 2-Arylquinazoline.

Based on the results of control experiments (entries 19–20 in Table 1, and eqs 3–4) and our previous studies, a possible reaction pathway for the organocatalytic oxidative formation of 2-arylquinazolines from two kinds of benzylamines is shown in Scheme 2.

First, benzylamine **2** reacts with 4,6-dihydroxysalicylic acid to form salt **A**, which undergoes hydrogen abstraction by O_2_ to generate aryloxy radical **B** and HOO•. The hydrogen abstraction of the benzyl group occurs intramolecularly to form radical cation **C** (Griller, et al., 1981; Nazran and Griller, 1983; Maclnnes, et al., 1987; Salamone, et al., 2011), which in turn affords imine **2′** under the action of HOO•. The subsequent amino group exchange reaction of **2’** with 2-aminobenzylamine **1** is smoothly proceeded in the presence of BF_3_‧Et_2_O to yield **D**. The intramolecular cyclization of **D** is accelerated by BF_3_‧Et_2_O to yield **E**. Finally, the oxidative aromatization of **E** results in the corresponding 2-arylquinazoline **3**.

In the quinazoline synthesis from benzylamine and *o*-aminobenzylamine, the most important point is the highly selective conversion of benzylamine to the corresponding imine. If *o*-aminobenzylamine undergoes imination, the resulting product cannot be converted into quinazoline. To solve this problem, quinazolines are typically synthesized by using excess amounts of benzylamine as an imine precursor, and the yield of quinazolines is calculated based on the lesser amount of *o*-aminobenzylamine used. However, in such methods, benzylidenebenzylamine (PhCH=NCH_2_Ph), which is formed by oxidative dimerization of benzylamine, is often produced as a byproduct, and the reaction system becomes complicated, which not only makes it time-consuming to isolate the quinazoline product, but also makes it difficult to scale up the reaction. In fact, there are no reports of gram-scale synthesis of related quinazolines reported so far. In contrast, in the present salicylic acid-catalyzed quinazoline synthesis method, the salicylic acid derivative predominantly forms a salt with benzylamine, and the imination proceeds exclusively for benzylamine. Therefore, the reaction could proceed with equimolar amounts of benzylamine and *o*-aminobenzylamine to give quinazoline derivatives in high yields. Noteworthy is that this method is not only excellent in E-factor (= 2.7), but also the best quinazoline synthesis method in terms of RME (= 73%). As shown in the ^1^H NMR spectrum of the unpurified crude product after the reaction (see, Supplementary Information), only quinazoline and solvent peaks could be detected in this system, and the reaction system is extremely clean. In addition, this is the only example of application of this method to the gram-scale synthesis of quinazolines from benzylamine and *o*-aminobenzylamine.

As shown in Table 2, the formation of 2-arylquinazoline scaffolds was catalyzed even using salicylic acid. Salicylic acid is a more common reagent compared to 4,6-dihydroxysalicylic acid. In order to make the synthesis of nitrogen-containing functional molecules industrially practical, it is necessary to optimize the catalytic system using salicylic acid as an organocatalyst. Therefore, we focused on using salicylic acid as the organocatalyst for the oxidation of benzylamines and its application to the practical synthesis of *N*-heterocycles. Our previous work has revealed that the catalytic reactivity of salicylic acid itself is somewhat lower than that of 4,6-dihydroxysalicylic acid for the oxidation of benzylamines (Dong et al., 2016). Therefore, we further optimized the reaction conditions in order to construct an oxidation system in which the salicylic acid catalyst works effectively (see the Supplementary Information).

**SCHEME 3:**
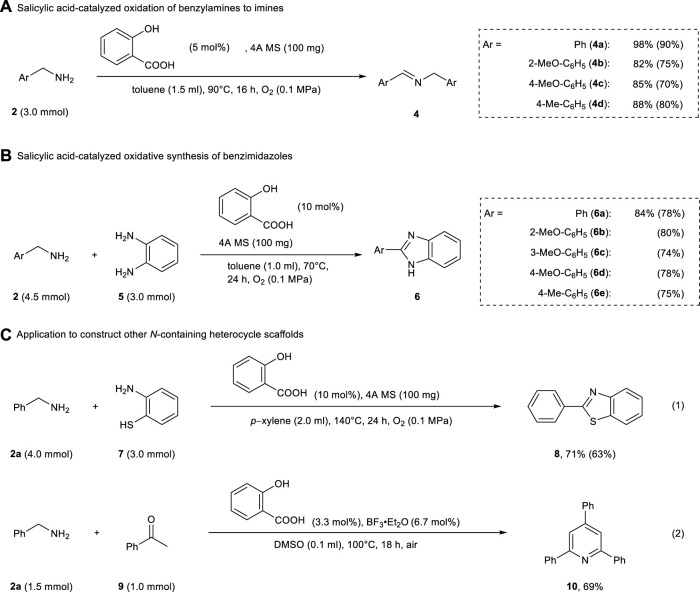
Salicylic acid-catalyzed oxidation of benzylamines and its application in the construction of *N*-Containing heterocycles. Yields were determined by ^1^H NMR spectroscopy (isolated yields). **(A)** Salicyclic acid-catalyzed oxidation of benzylamines to imines. **(B)** Salicyclic acid-catalyzed oxidative synthesis of benzimidazoles. **(C)** Application to construct other *N*-containing heterocycle scaffoids.

The oxidation of benzylamine proceeded well with 5 mol% of salicylic acid and 4A MS (to inhibit hydrolysis of the formed imine), yielding the corresponding imines **4a**–**4e** in 82–98% yields (Scheme 3A). In addition, the oxidative condensation of benzylamine **2** and 1,2-phenylenediamine **5** in the presence of salicylic acid (10 mol%) and 4A MS (100 mg) afforded various benzimidazoles **6a–6e** in good yields (Scheme 3B). Under similar conditions, 2-phenylbenzothiazole **8** was obtained from benzylamine **2a** and 2-aminobenzenethiol **7** in 71% yield (eq 1, Scheme 3C). This salicylic acid-catalyzed oxidation of benzylamines was also successfully applied to the one-pot synthesis of 2,4,6-triphenylpyridine (eq 2, Scheme 3C). As described above, our method was as effective as or more effective than the system using 4,6-dihydroxysalicylic acid for the construction of *N*-heterocycles.

As illustrated in Scheme 3, the key step was the organocatalytic oxidation of benzylamines to imines. Considering bulk synthesis via these organocatalytic reactions, it is important that the oxidation of benzylamines proceeds smoothly, even when scaled up for the synthesis of practical *N*-containing functional molecules for pharmaceutical and industrial applications. The salicylic acid-catalyzed oxidation of benzylamine **2a** could be successfully conducted under the neat condition at the scale of 110 mmol, and the corresponding imine **4a** was isolated in 94% yield (Table 3). Thus, this salicylic acid-catalyzed oxidative transformation of benzylamines can be an environmentally friendly, useful, and low-cost synthetic method in organic chemistry.

**TABLE 3:** Optimization of multi-gram-scale synthesis of imines via the salicylic acid-catalyzed green oxidation of benzylamine.
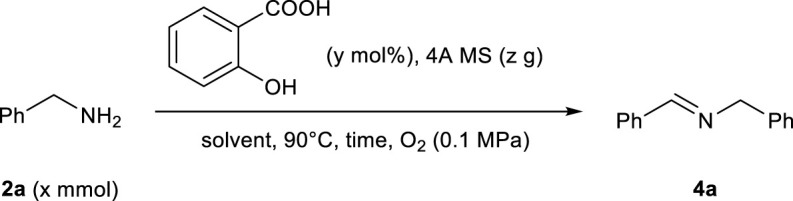

Entry	2a (mmol)	Cat. (mol%)	4A MS (g)	Solvent (ml)	Time (h)	Yield 4a (%)*^a^*
1	30	5	—	Toluene (15)	16	29
2	30	5	—	Neat	16	45
3	30	10	—	Neat	16	72
4	100	10	—	Neat	16	26
5	100	10	1	Neat	48	58
6	110	10	1	Neat	72	(94)

a Yields were determined by ^1^H NMR spectroscopy (isolated yields).

## Conclusion

In this study, we developed a metal-free method for the synthesis of 2-substituted quinazoline derivatives via the oxidative condensation of *o*-aminobenzylamines with benzylamines using 4,6-dihydroxysalicylic acid as the catalyst under atmospheric oxygen. Since the construction of the quinazoline scaffolds involves a highly multistep reaction, conventional methods often required an excess amount of substrate to control the product selectivity, resulting in a high amount of wastage. In contrast, our method could be conducted under mild conditions, and the corresponding quinazolines could be obtained with excellent atom economy, an E-factor of 2.7, and RME of 73%. Furthermore, this excellent eco-friendly system could achieve the synthesis of quinazolines up to a scale of 10 mmol, for the first time. Interestingly, the organocatalytic construction of quinazolines could be carried out using only salicylic acid, and the salicylic acid-catalyzed oxidation system could be applied to the green and practical synthesis of a series of nitrogen-containing functional compounds. We expect that the development of this environmentally friendly salicylic acid-catalyzed oxidation system will provide practical synthesis methods for pharmaceutical manufacturing and industrial applications, and contribute to further development in green chemistry.

## Data Availability

The raw data supporting the conclusion of this article will be made available by the authors, without undue reservation.
