# Supplementary material for: Metal-Free Synthesis of 2-Substituted Quinazolines via Green Oxidation of o-Aminobenzylamines: Practical Construction of N-Containing Heterocycles Based on a Salicylic Acid-Catalyzed Oxidation System
Source: Front Chem. 2022 Feb 23;9:822841. doi: 10.3389/fchem.2021.822841 (PMC8905626; doi:10.3389/fchem.2021.822841)

Supporting Information

Metal-Free Synthesis of 2-Substituted Quinazolines via Green Oxidation of *o*-Aminobenzylamines: Practical Construction of *N*-Containing Heterocycles Based on a Salicylic Acid-Catalyzed Oxidation System

Yuki Yamamoto[1], Chihiro Yamakawa[1], Riku Nishimura[1], Chun-ping Dong[1],

Shintaro Kodama[1*], Akihiro Nomoto[1], Michio Ueshima[1], and Akiya Ogawa[1*]

[1]Department of Applied Chemistry, Graduate School of Engineering, Osaka Prefecture University, 1-1 Gakuen-cho, Nakaku, Sakai, Osaka 599-8531, Japan

skodama@chem.osakafu-u.ac.jp, ogawa@chem.osakafu-u.ac.jp

CONTENTS

	Pages
**Scheme S1**. Comparison of the E-factor of some alternative methods for synthesis of quinazolines and this work	S2
**Figure S1.** ^{1}H NMR spectrum of crude **3aa** after the reaction (entry 17 in Table 1)	S3
**Table S1**. Optimization of reaction conditions for salicylic acid-catalyzed oxidation of benzylamine to the corresponding imine	S3
Copies of ^{1}H and ^{13}C{^{1}H} NMR spectra of compounds **3aa**–**3ah**, **3aj–3at**, and **3ba**	S4–S23
Copies of ^{1}H and ^{13}C{^{1}H} NMR spectra of compounds **4a**–**4d**	S24–S27
Copies of ^{1}H and ^{13}C{^{1}H} NMR spectra of compounds **6a**–**6e**, **8**, and **9**	S28–S34

Scheme S1. Comparison of the E-factor of some alternative methods for synthesis of quinazolines and this work

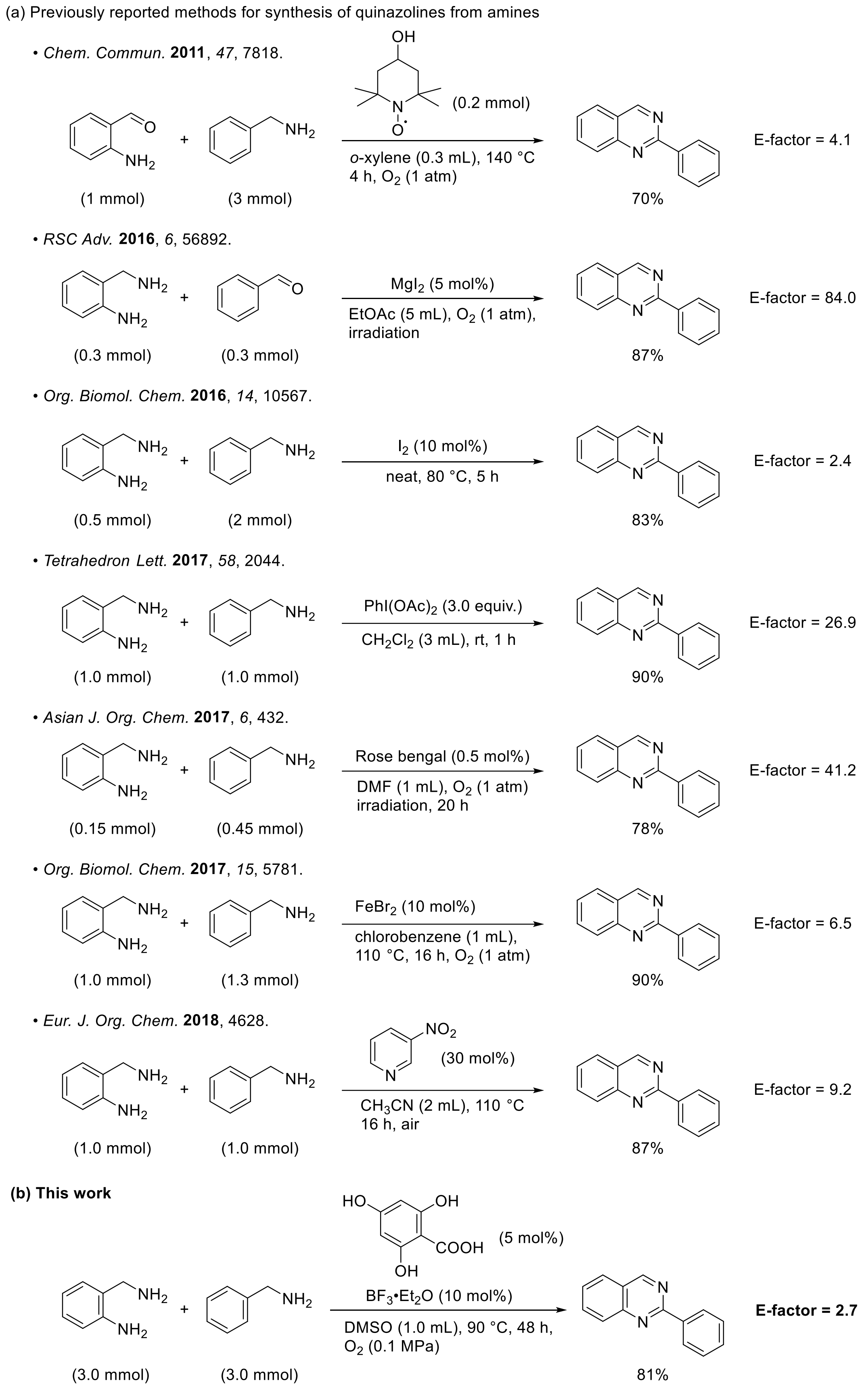

Figure S1. ^{1}H NMR spectrum of crude **3aa** after the reaction (entry 17 in Table 1, in $CDCl_3$)

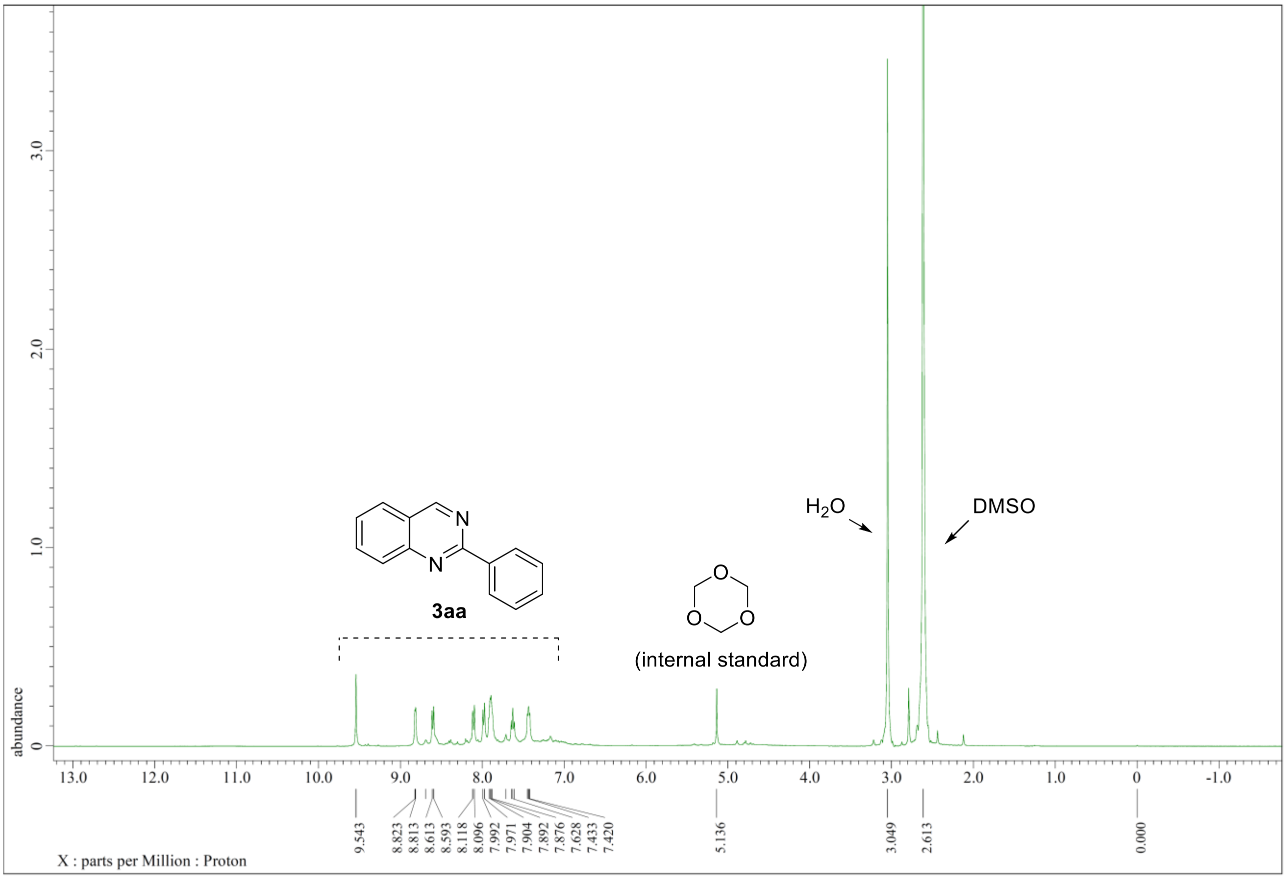

Table S1. Optimization of reaction conditions for salicylic acid-catalyzed oxidation of benzylamine to the corresponding imine

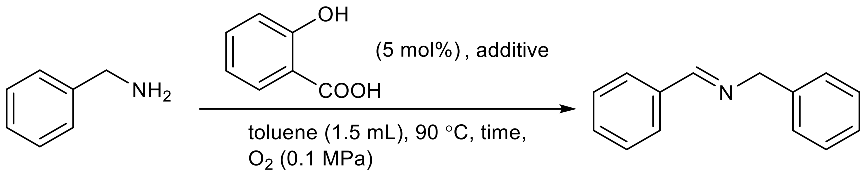

Entry	Additive (mg)	Time (h)	Yield **4a** (%)[a]
1	-	2	14
2	-	4	26
3	-	16	79
4	-	24	73
5	4A MS (100)	16	98 (87)

[a]Yields were determined by ^{1}H NMR spectroscopy (isolated yield)

Figure S2: Copies of ^{1}H and ^{13}C{^{1}H} NMR spectra of compound **3aa**

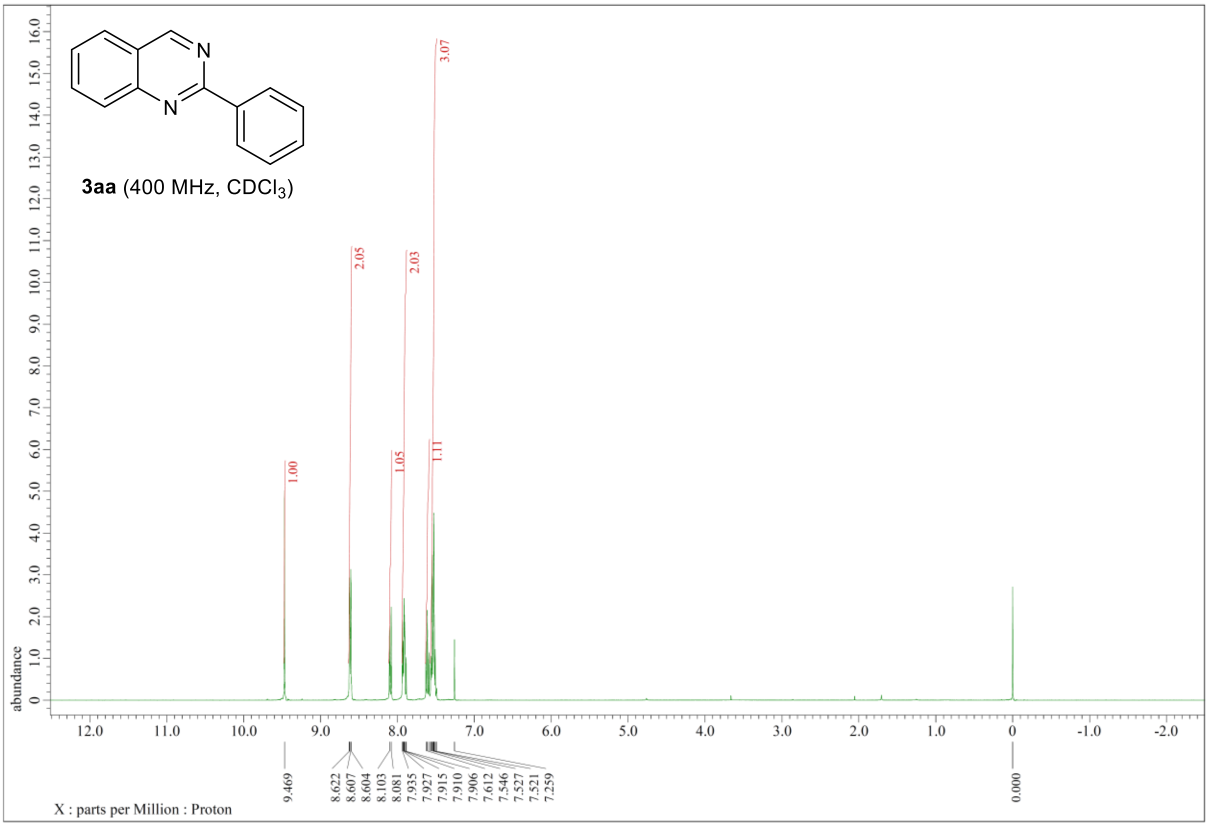

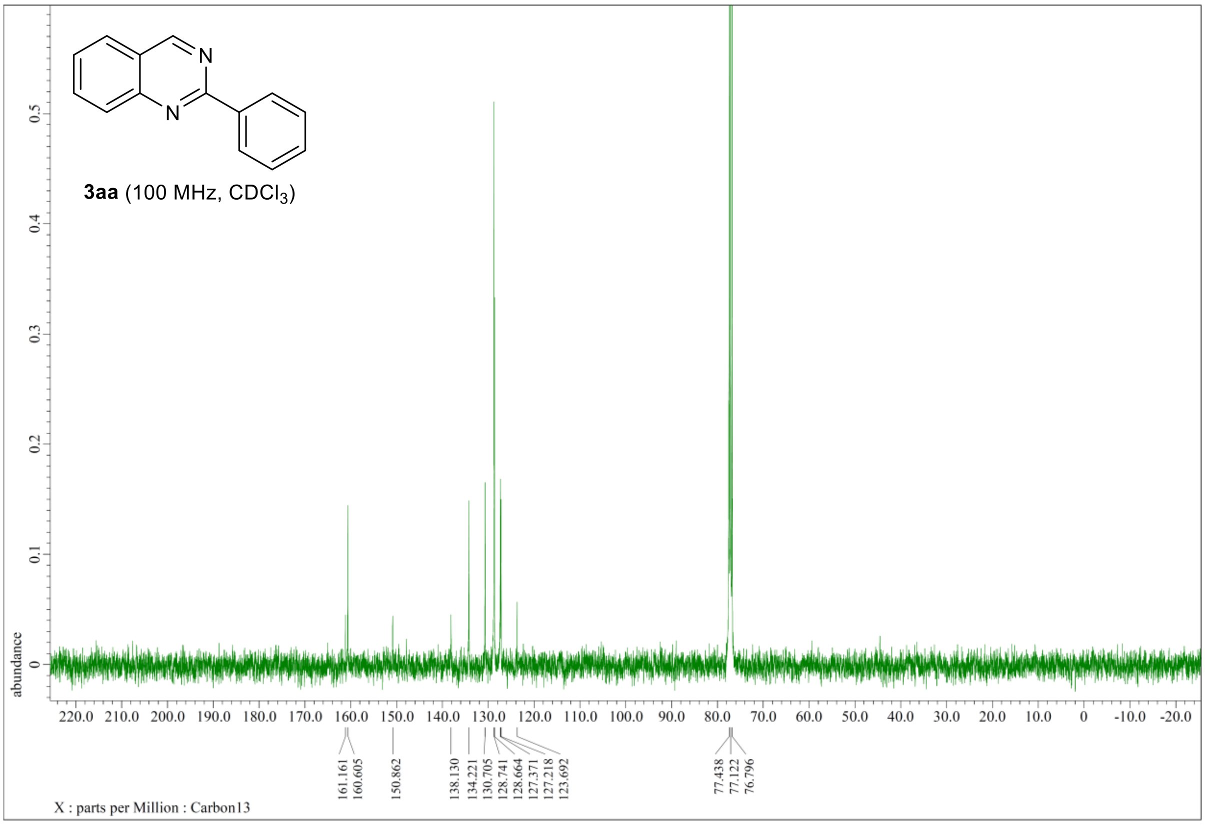

Figure S3: Copies of ^{1}H and ^{13}C{^{1}H} NMR spectra of compound **3ab**

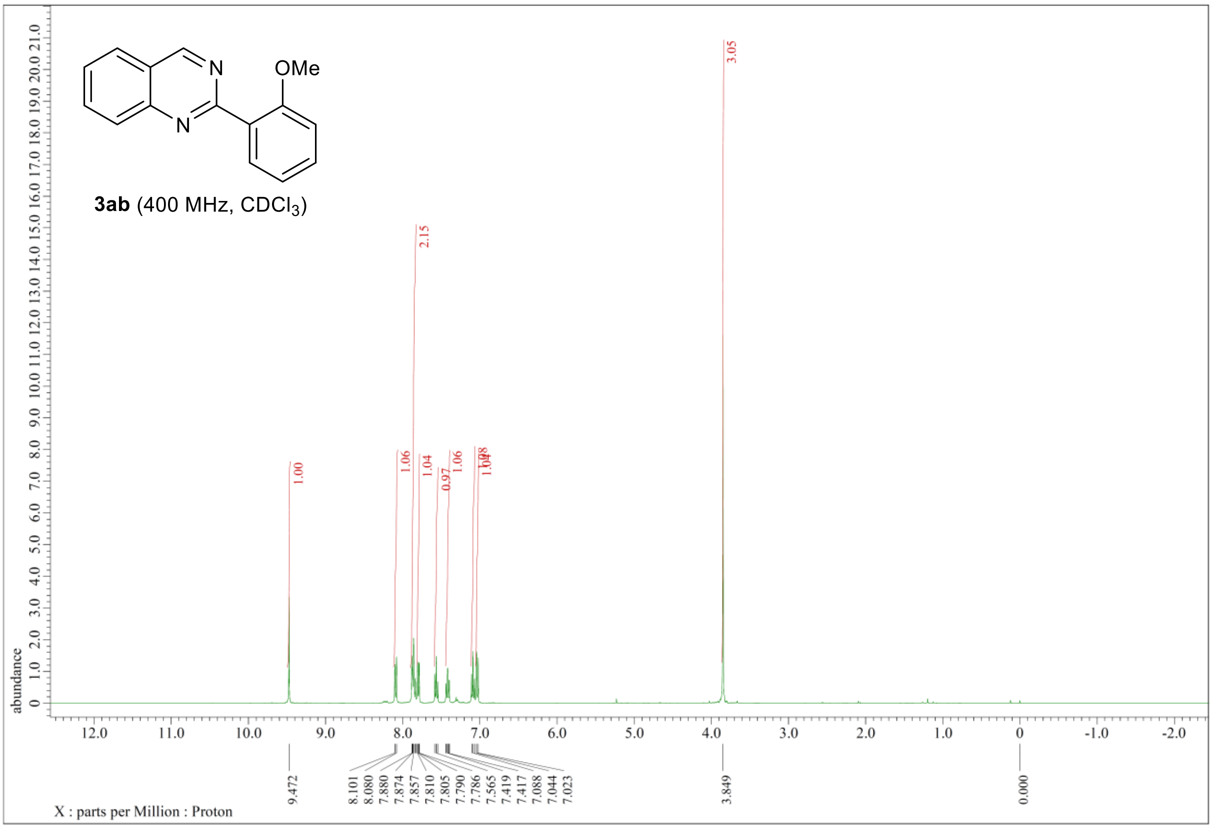

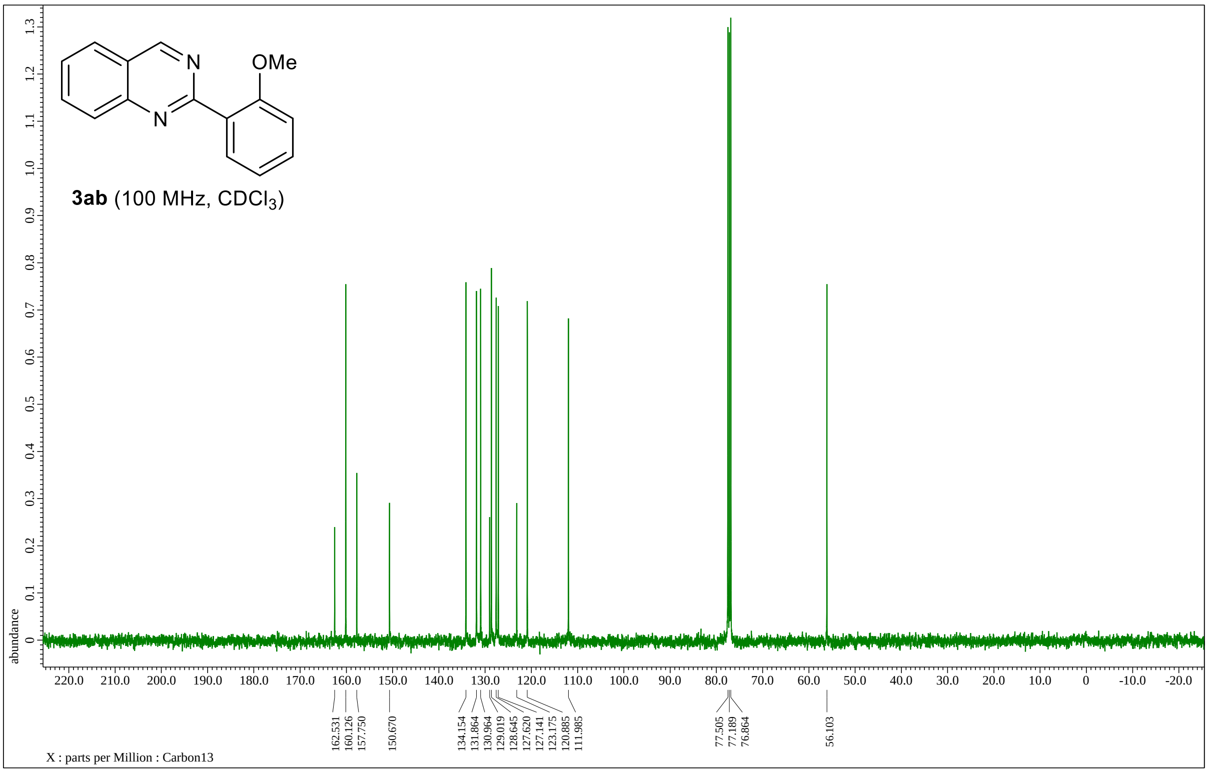

Figure S4: Copies of ^{1}H and $^{13}C\{^{1}H\}$ NMR spectra of compound **3ac**

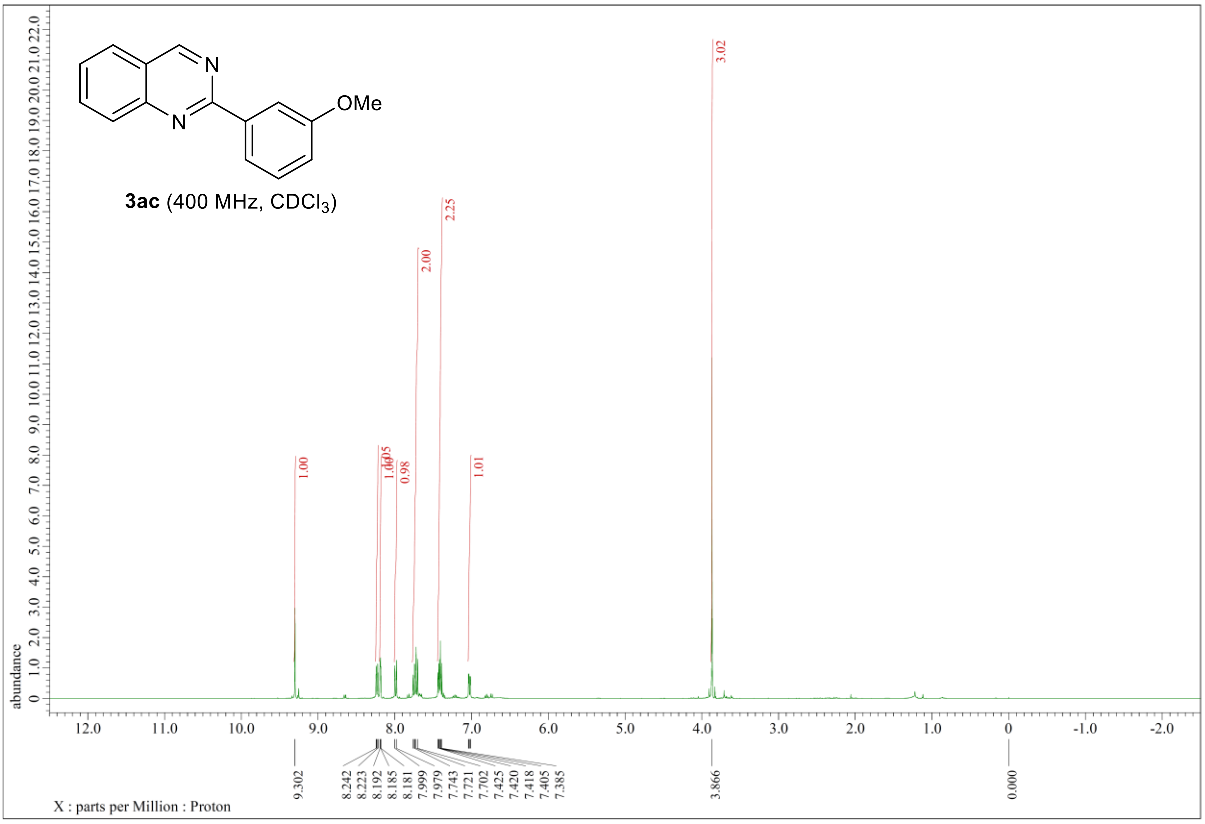

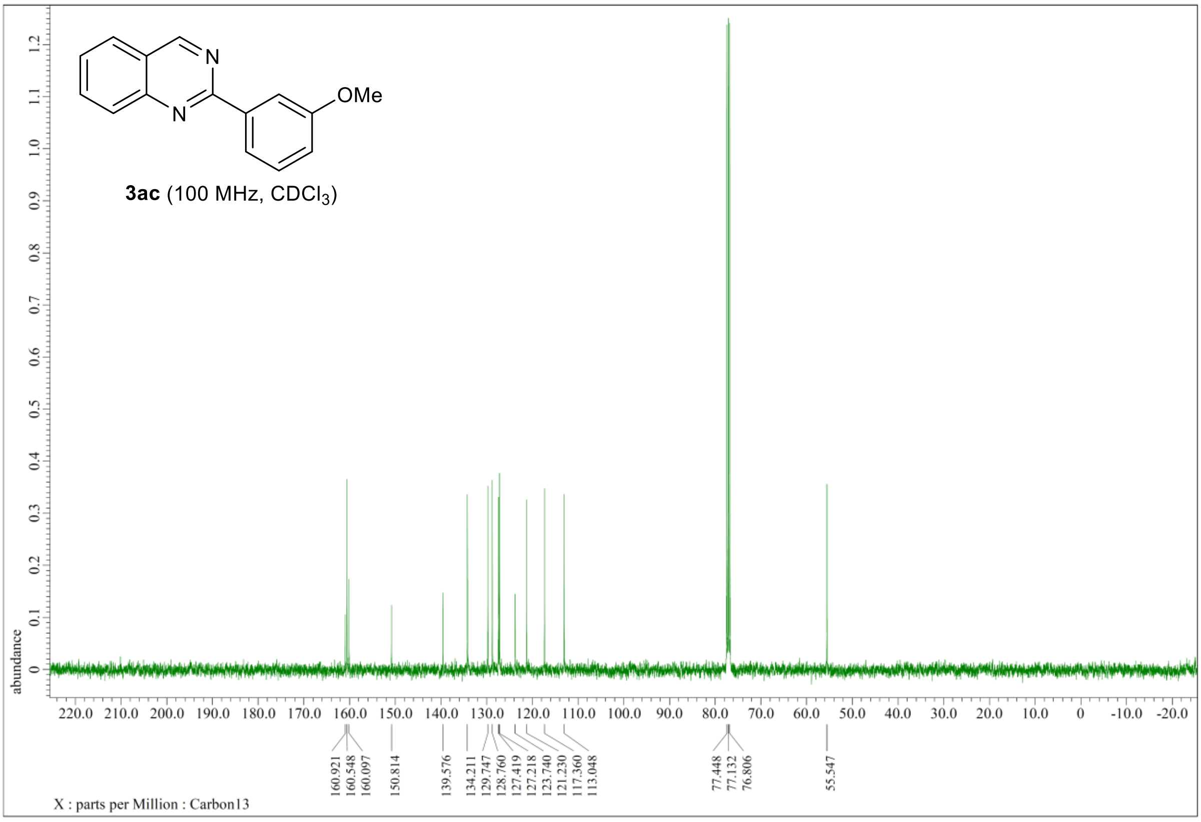

Figure S5: Copies of ^{1}H and ^{13}C{^{1}H} NMR spectra of compound **3ad**

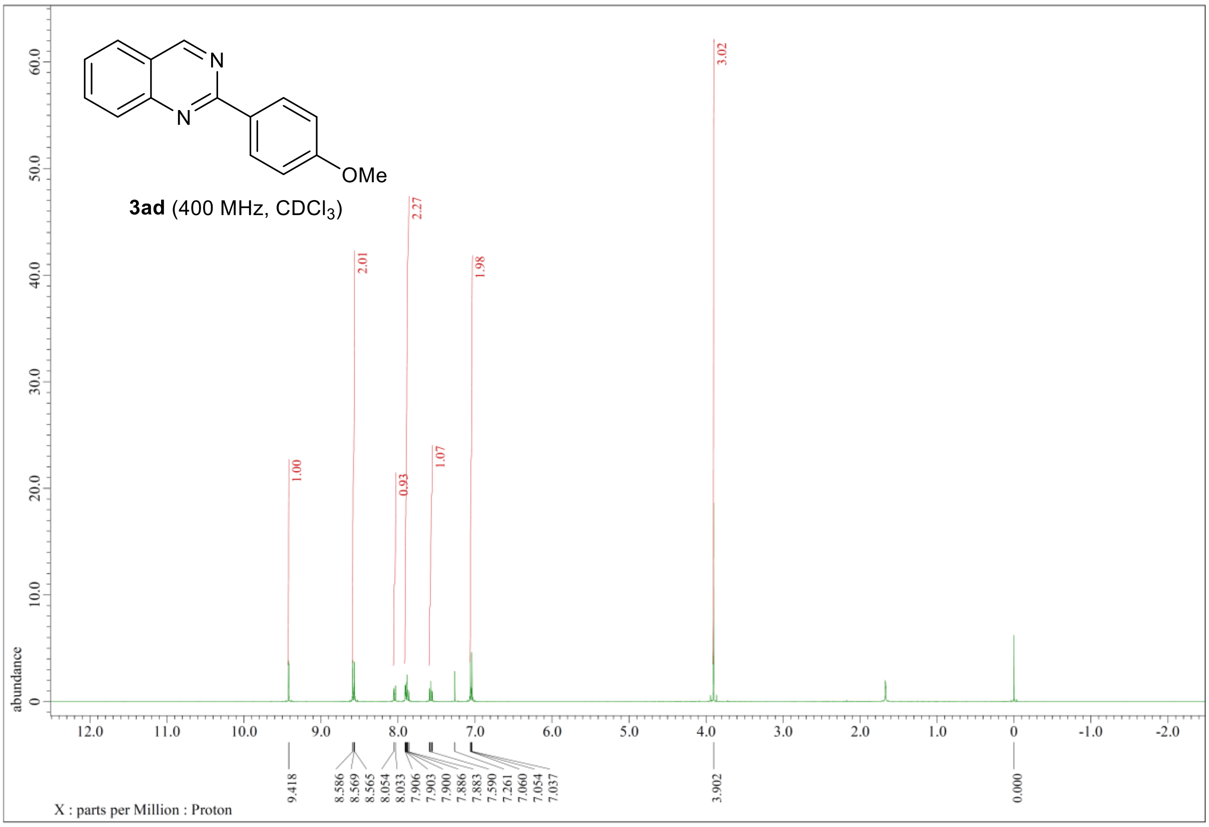

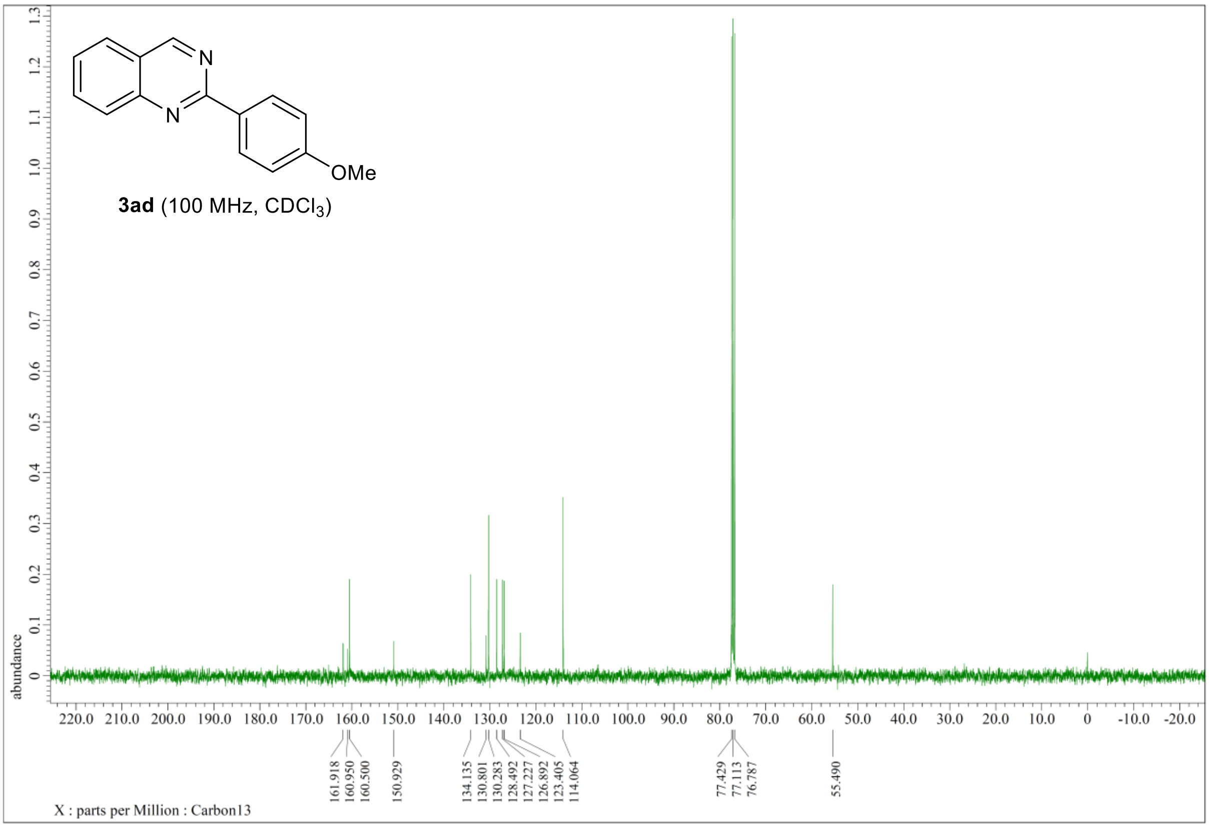

Figure S6: Copies of ^{1}H and ^{13}C{^{1}H} NMR spectra of compound **3ae**

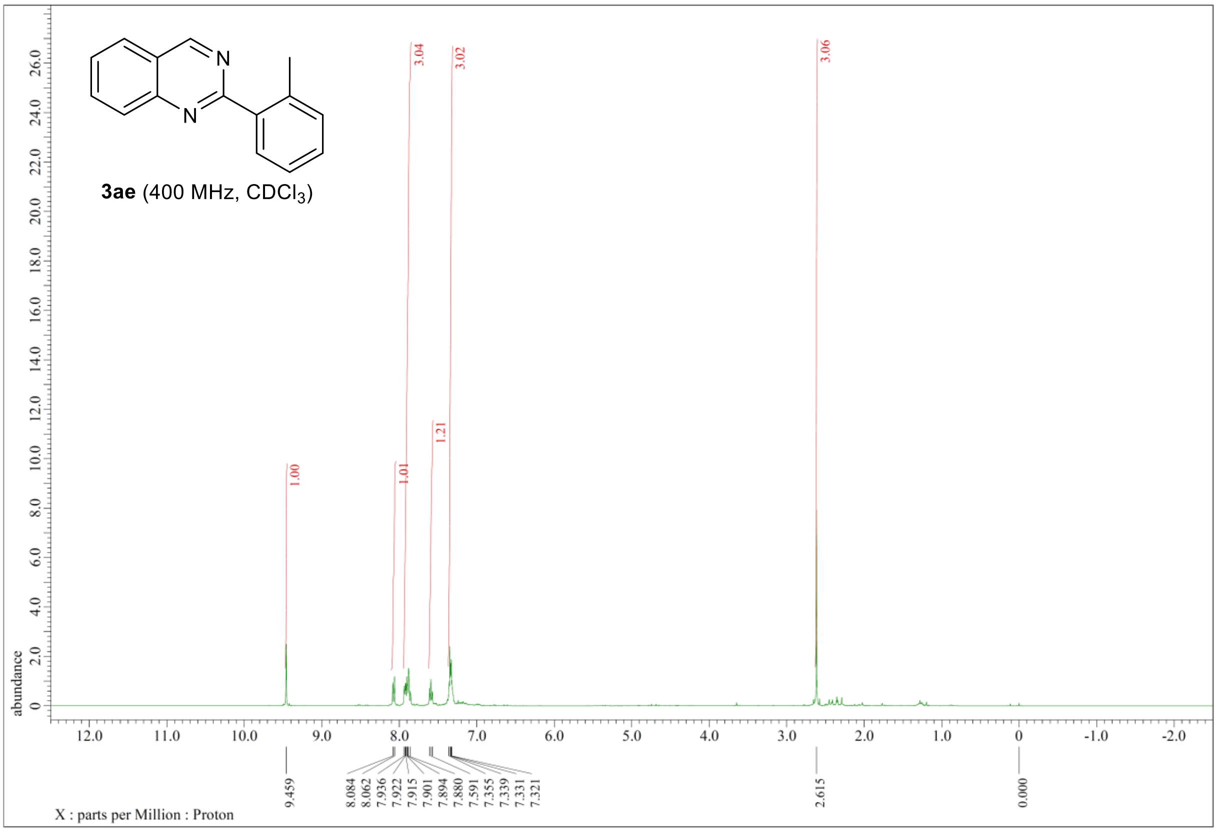

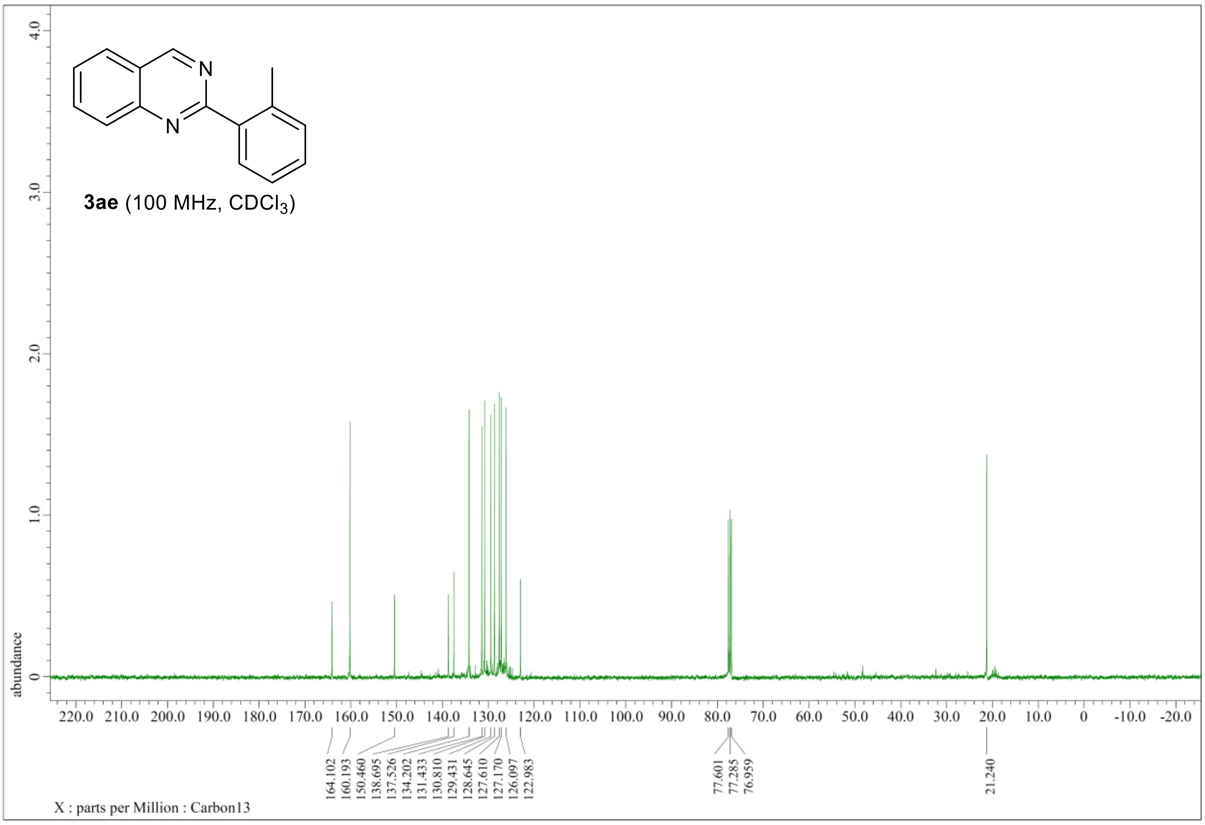

Figure S7: Copies of ^{1}H and $^{13}C\{^{1}H\}$ NMR spectra of compound **3af**

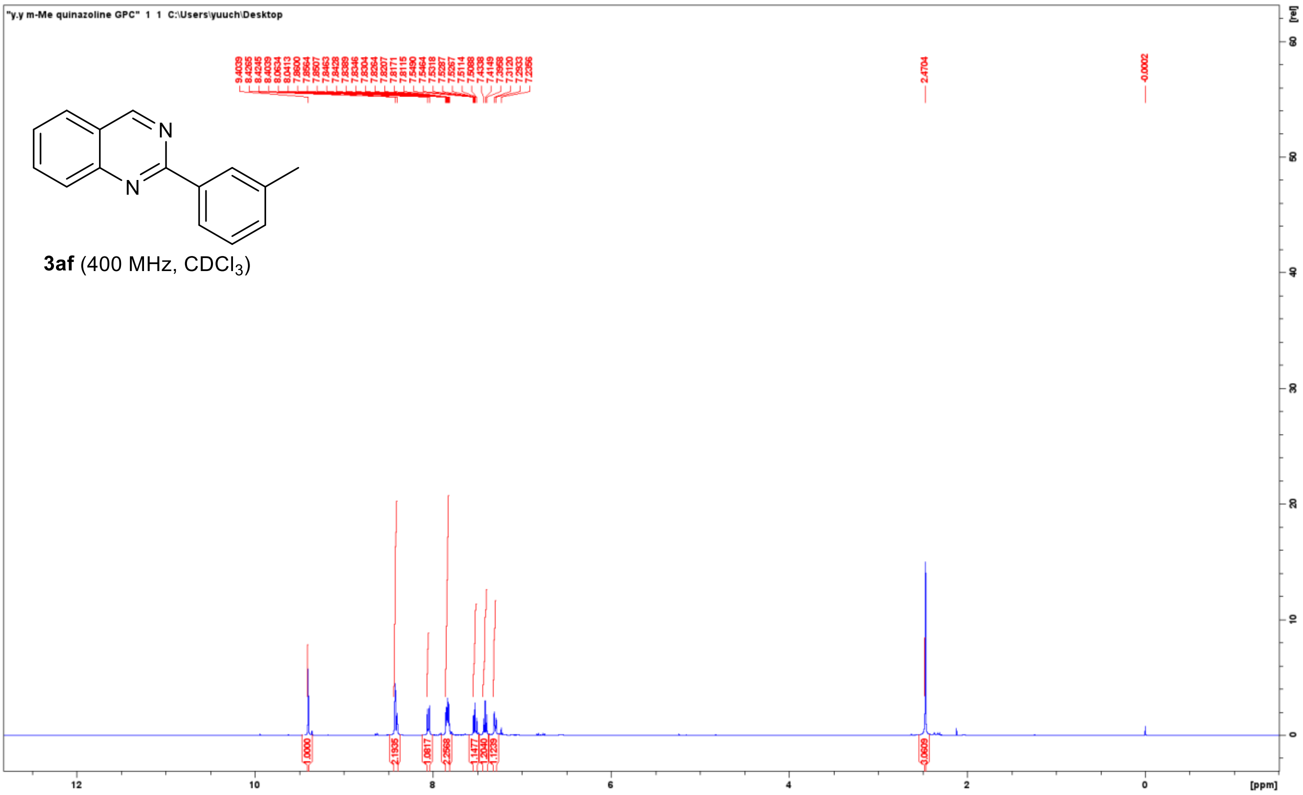

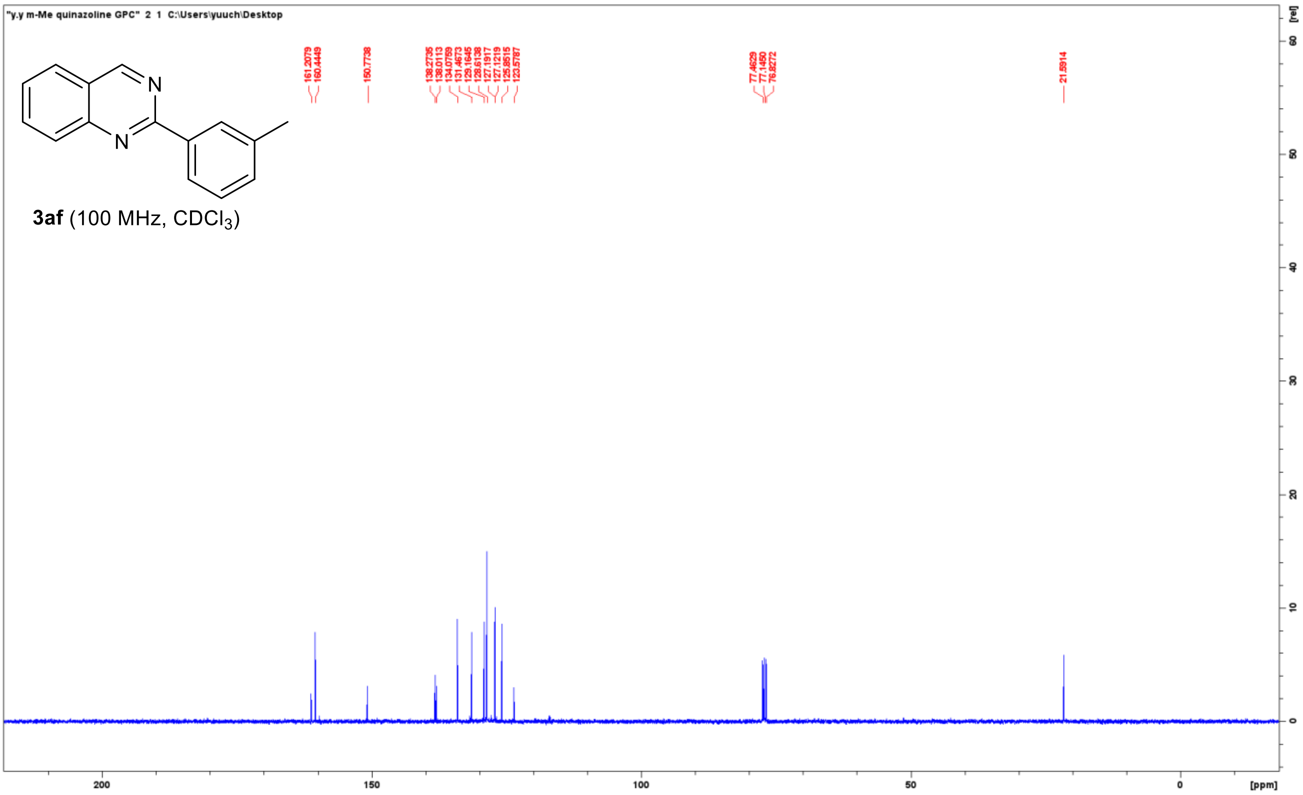

Figure S8: Copies of ^{1}H and ^{13}C{^{1}H} NMR spectra of compound **3ag**

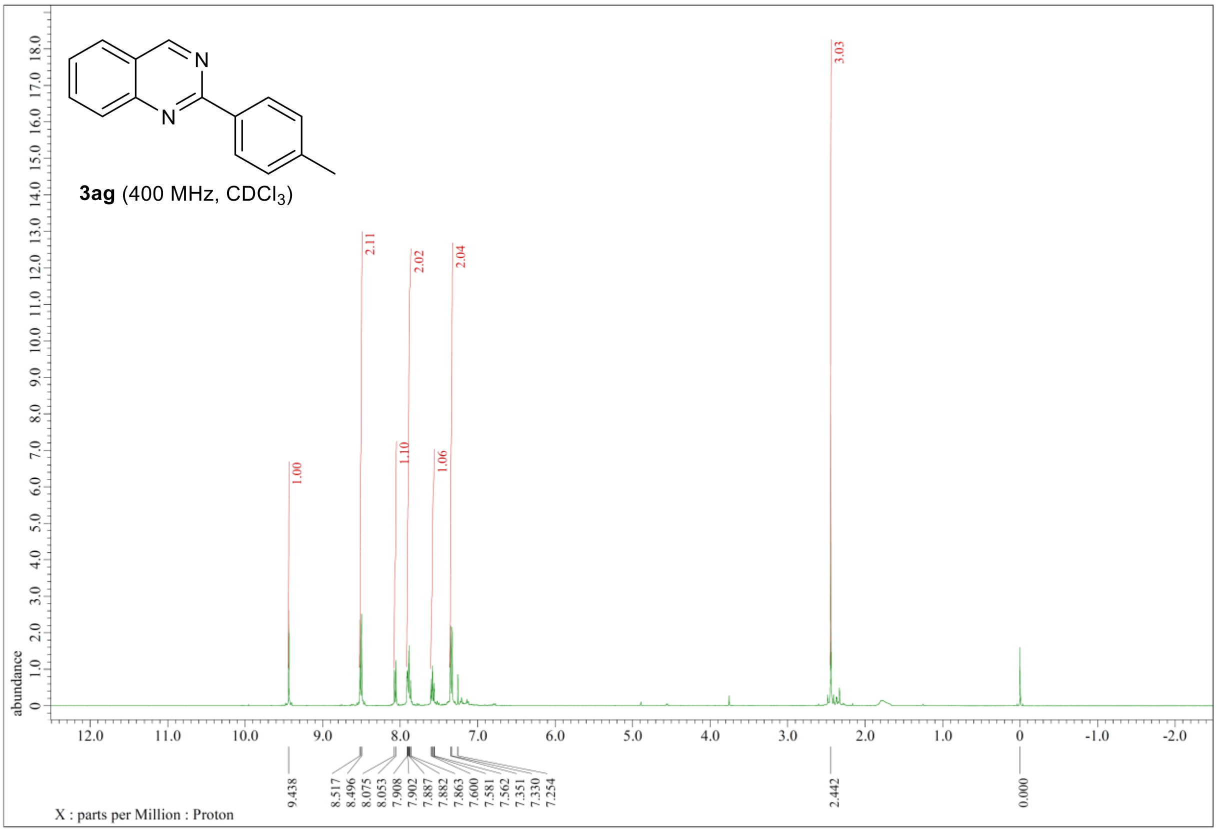

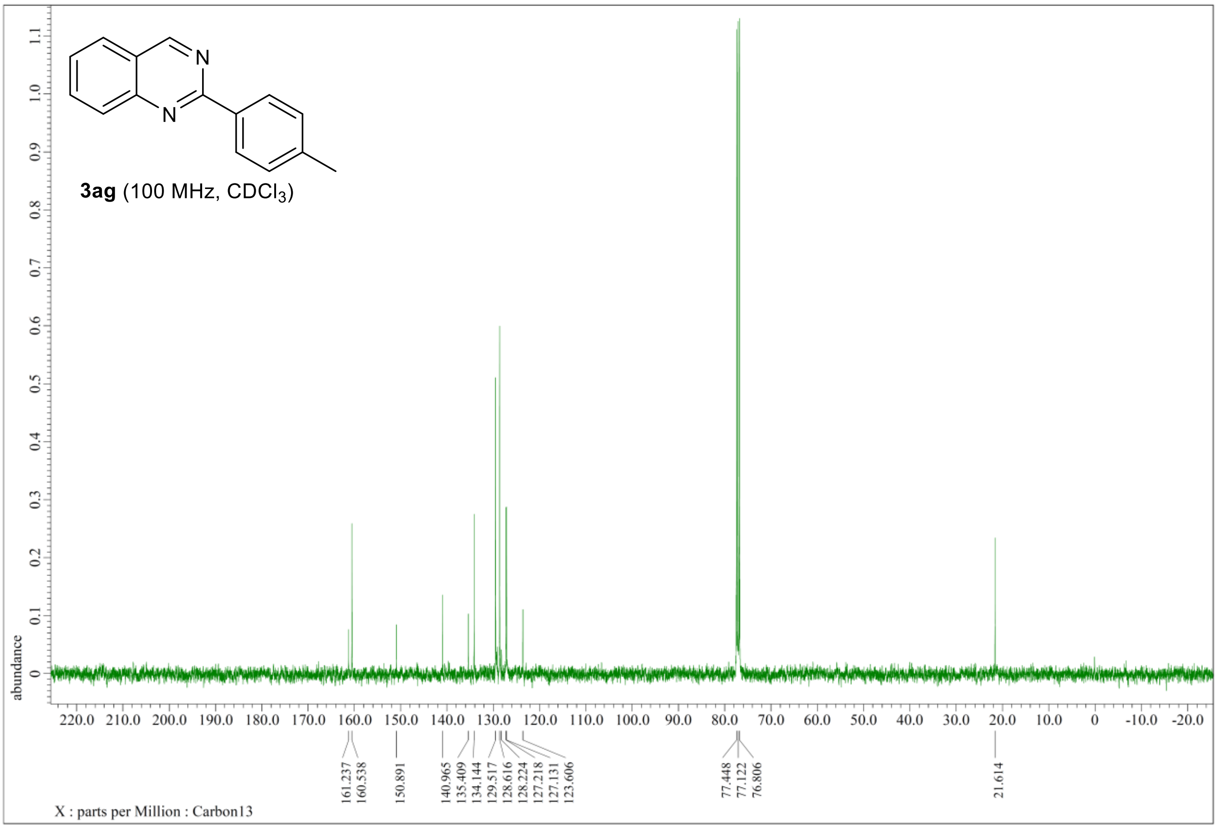

Figure S9: Copies of ^{1}H and ^{13}C{^{1}H} NMR spectra of compound **3ah**

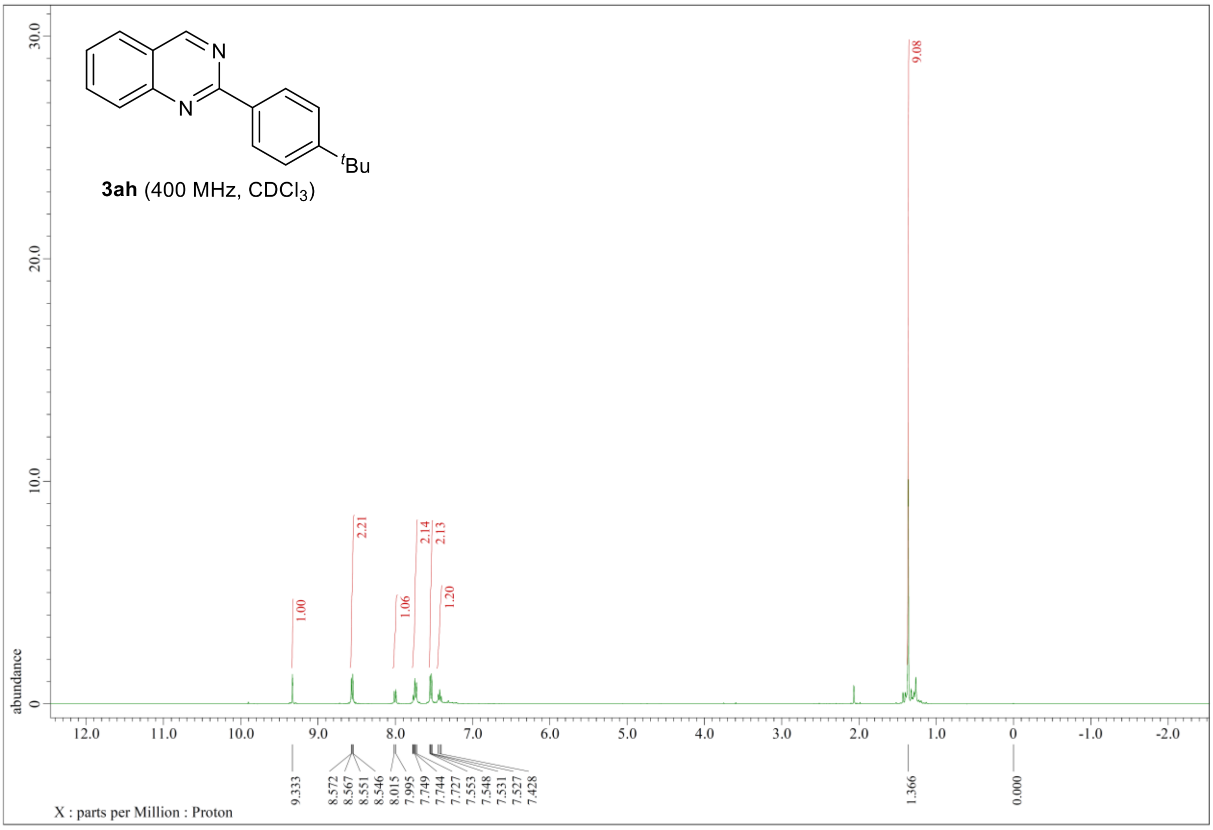

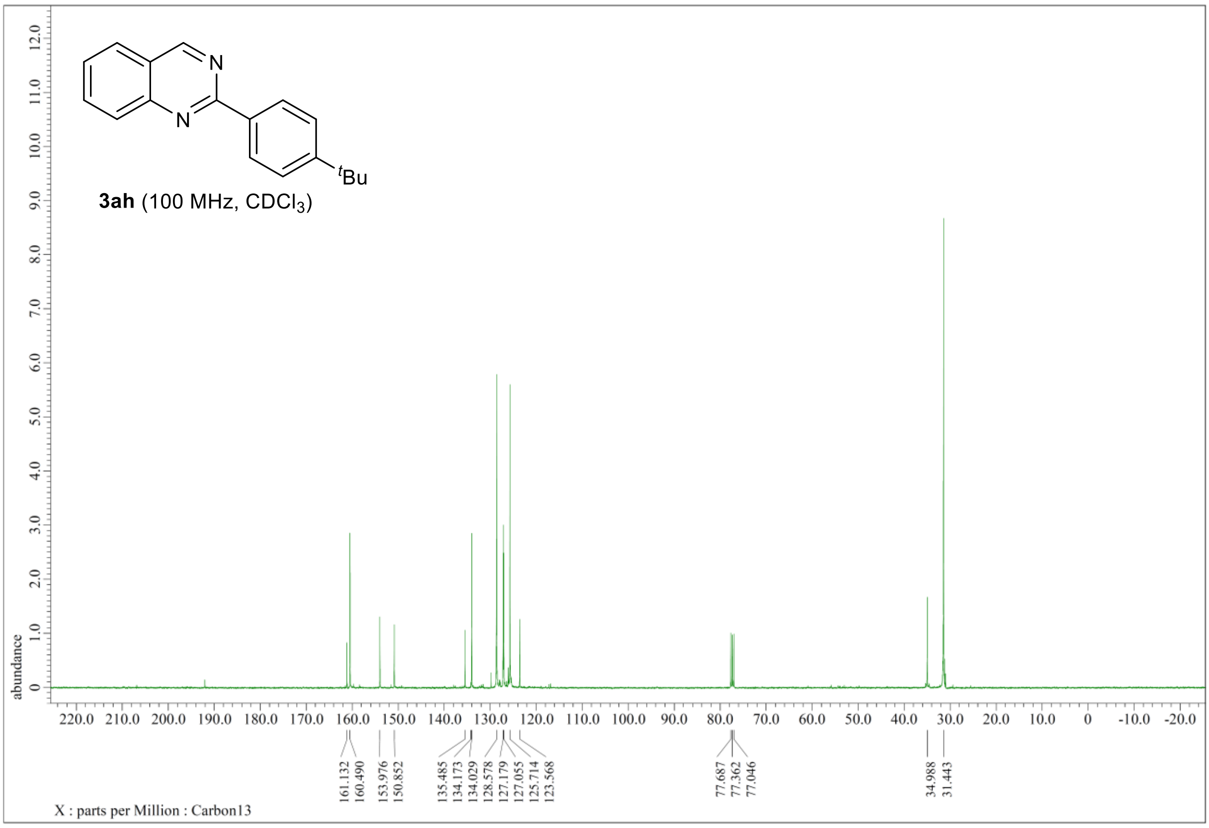

Figure S10: Copies of ^{1}H and $^{13}C\{^{1}H\}$ NMR spectra of compound **3aj**

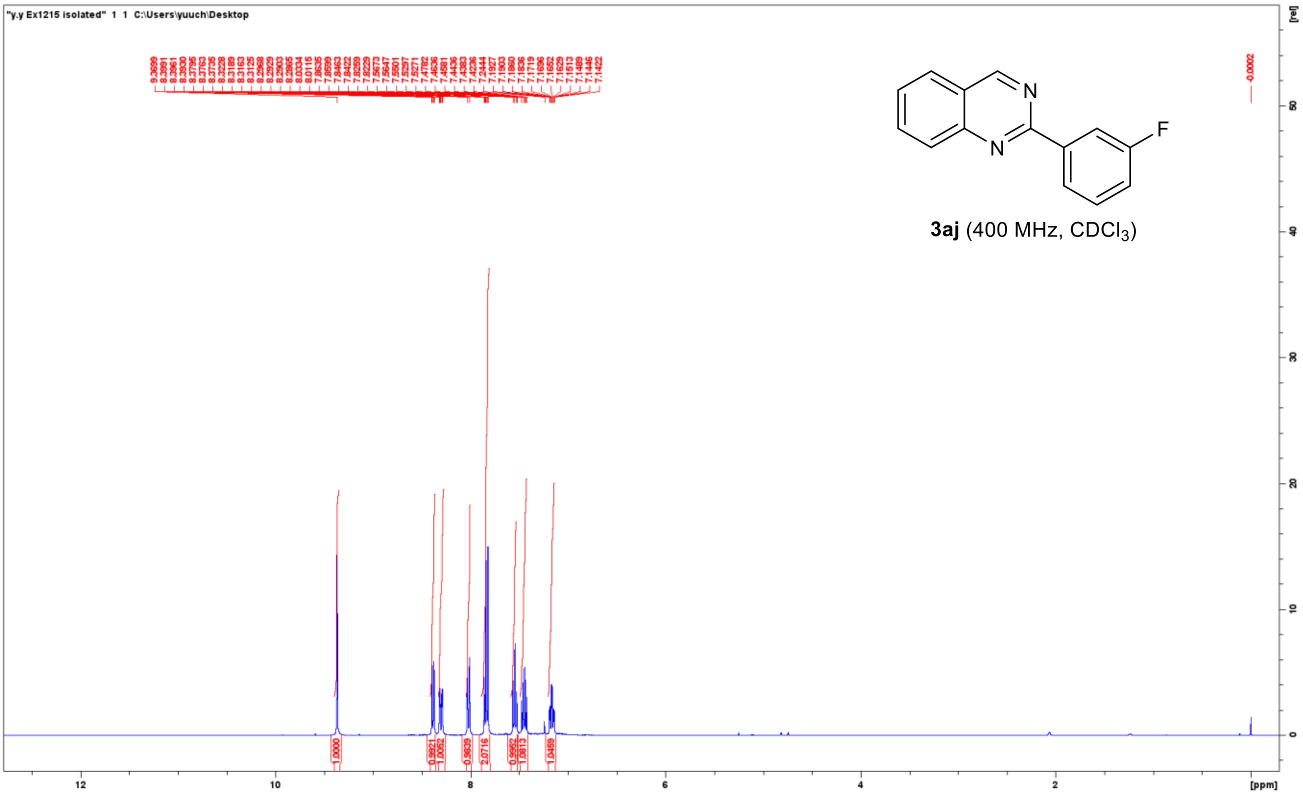

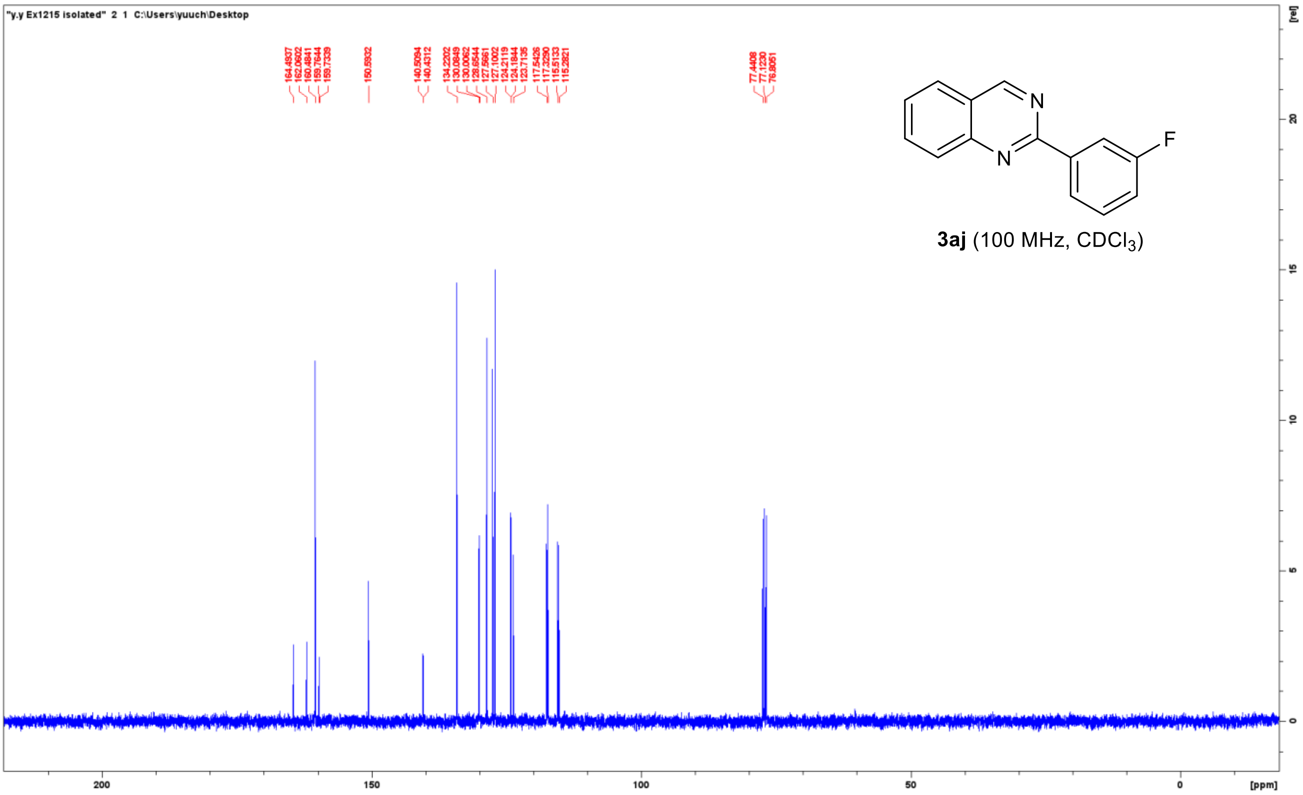

Figure S11: Copies of ^{1}H and $^{13}C\{^{1}H\}$ NMR spectra of compound **3ak**

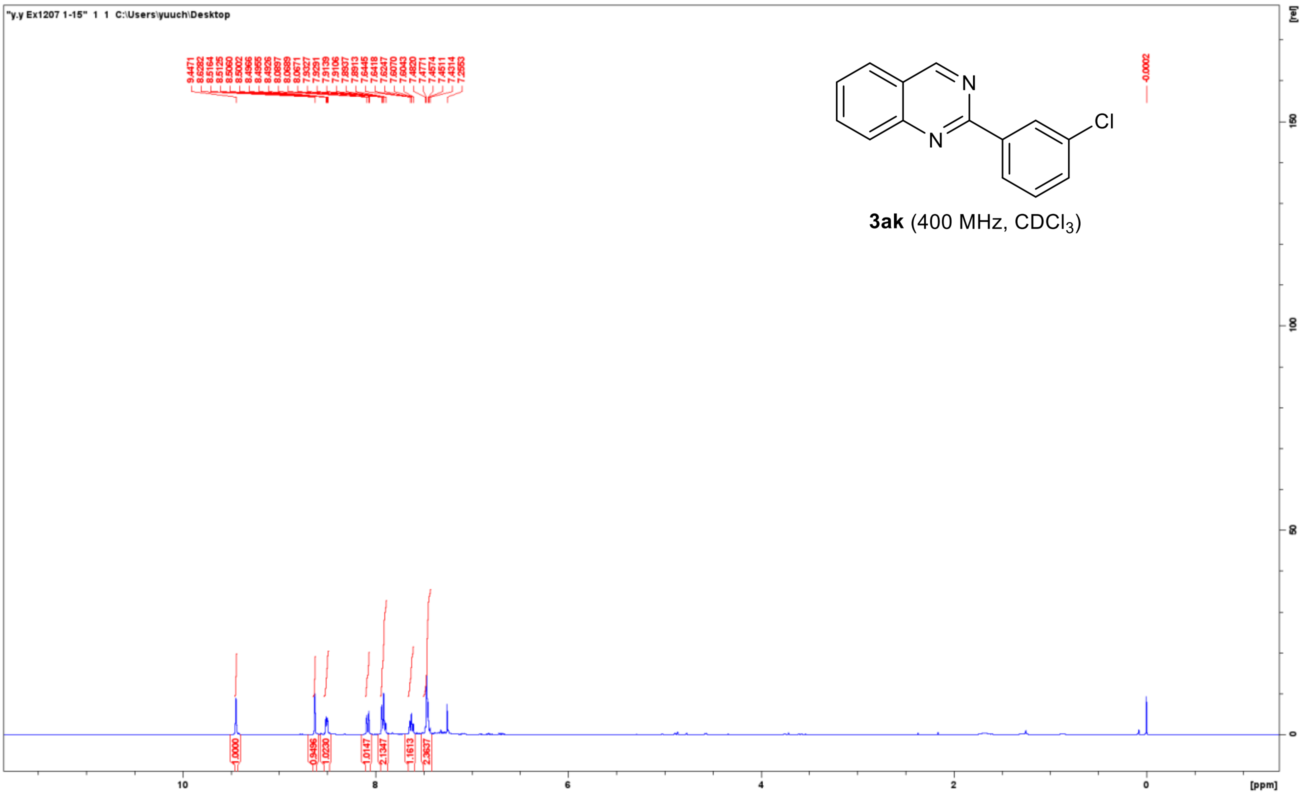

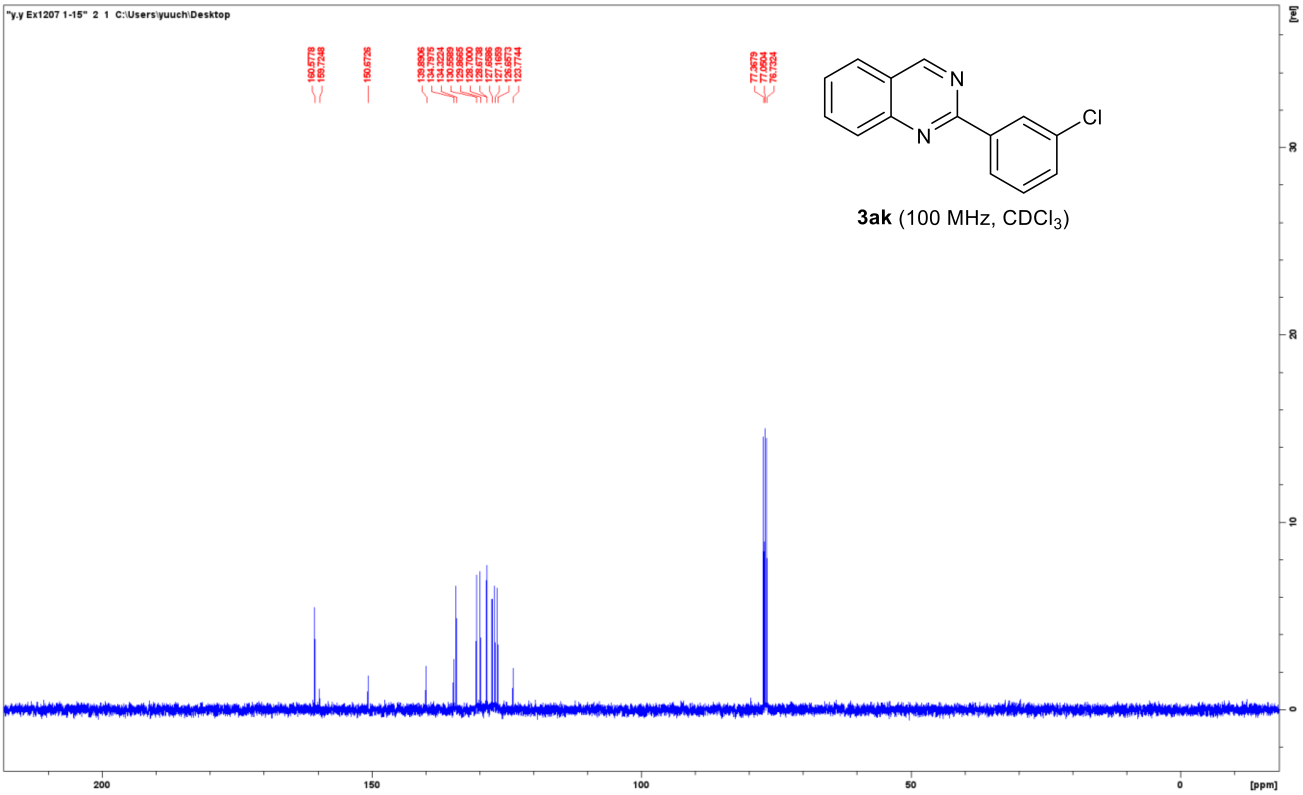

Figure S12: Copies of ^{1}H and ^{13}C{^{1}H} NMR spectra of compound **3al**

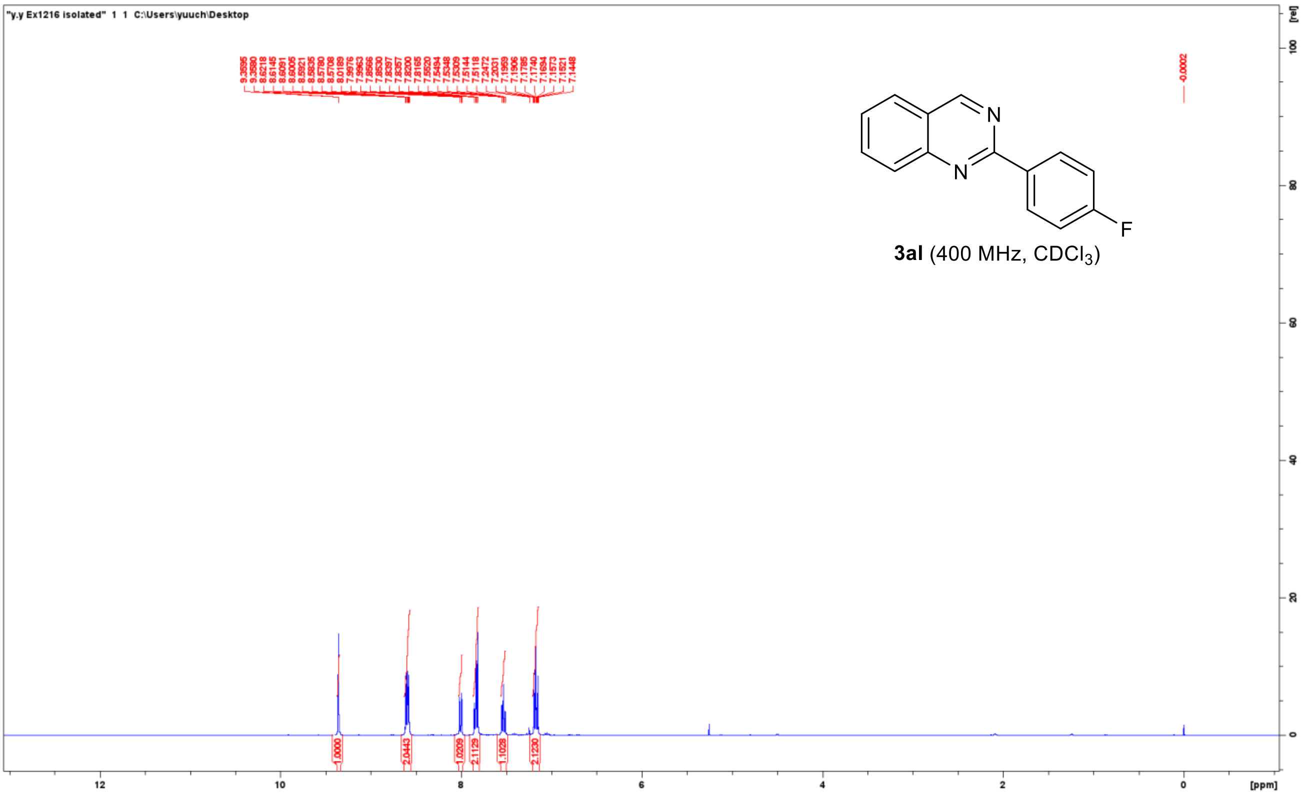

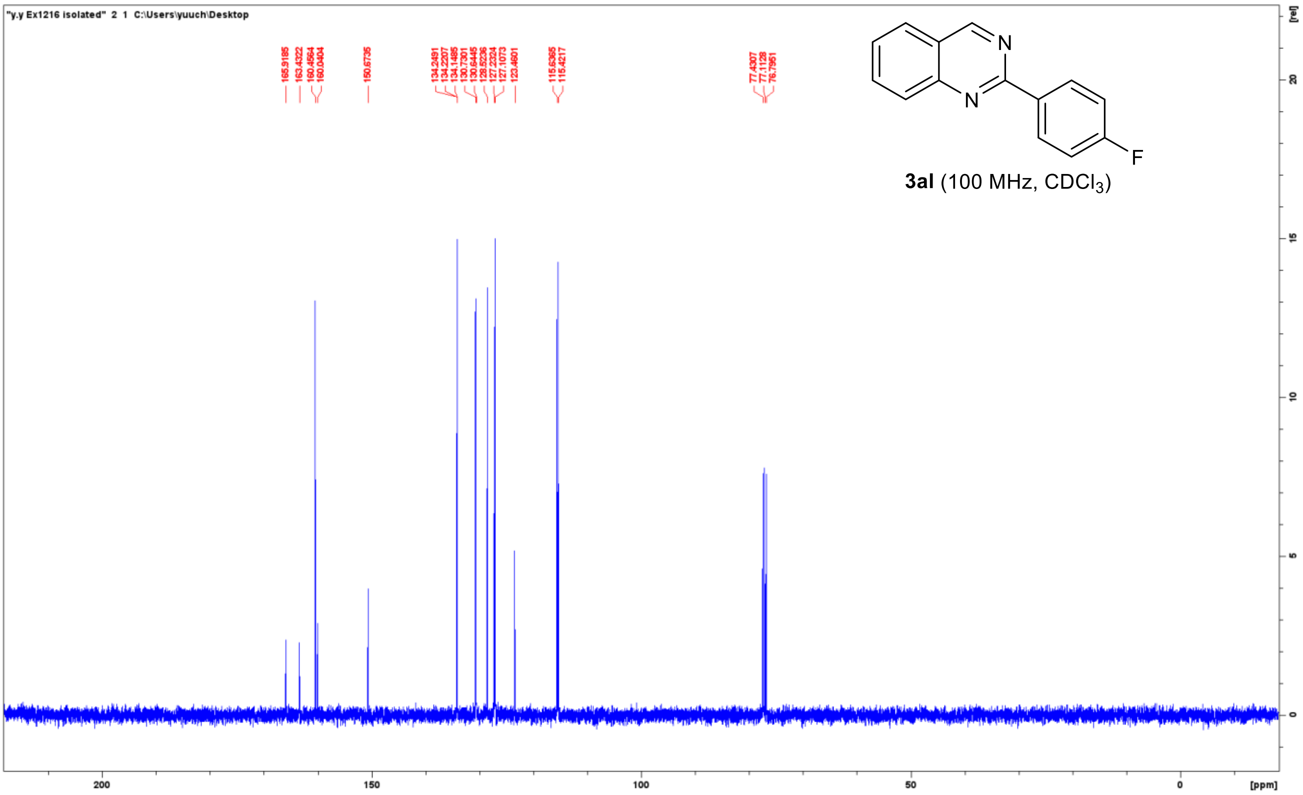

Figure S13: Copies of ^{1}H and ^{13}C{^{1}H} NMR spectra of compound **3am**

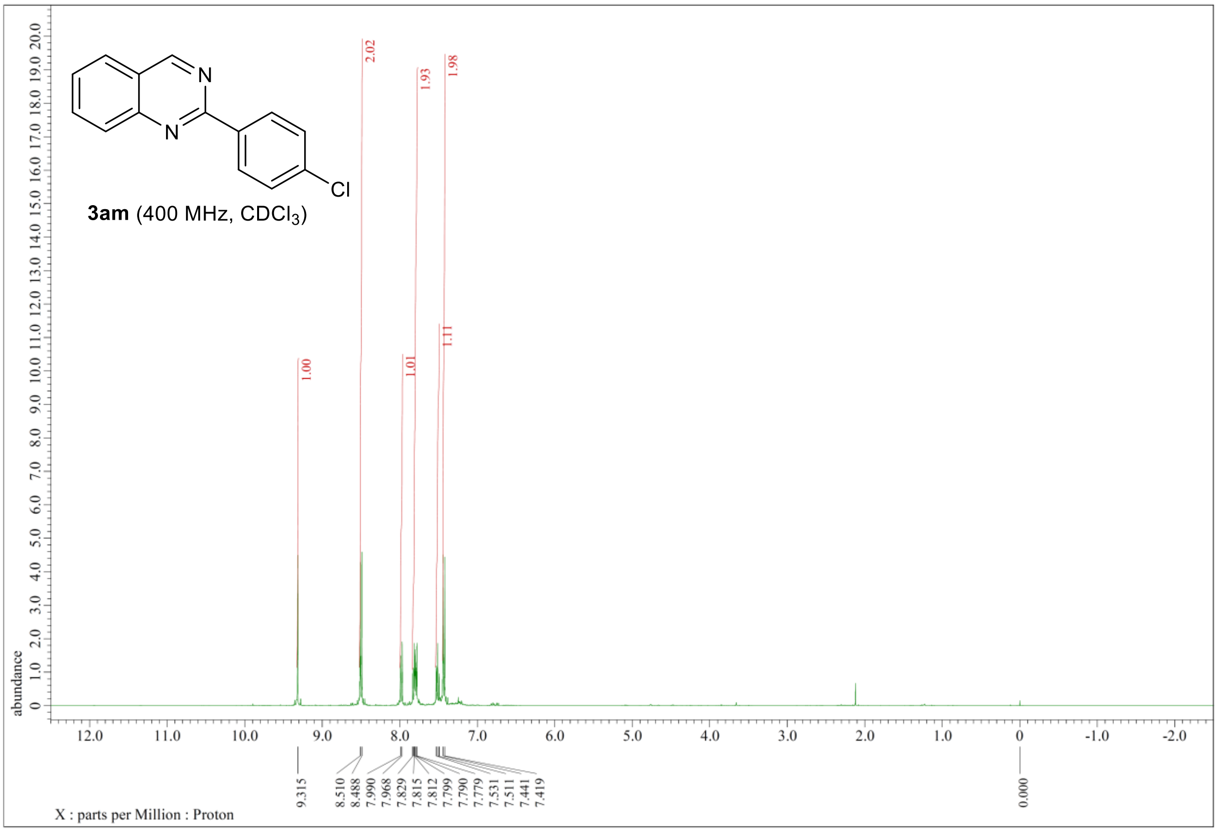

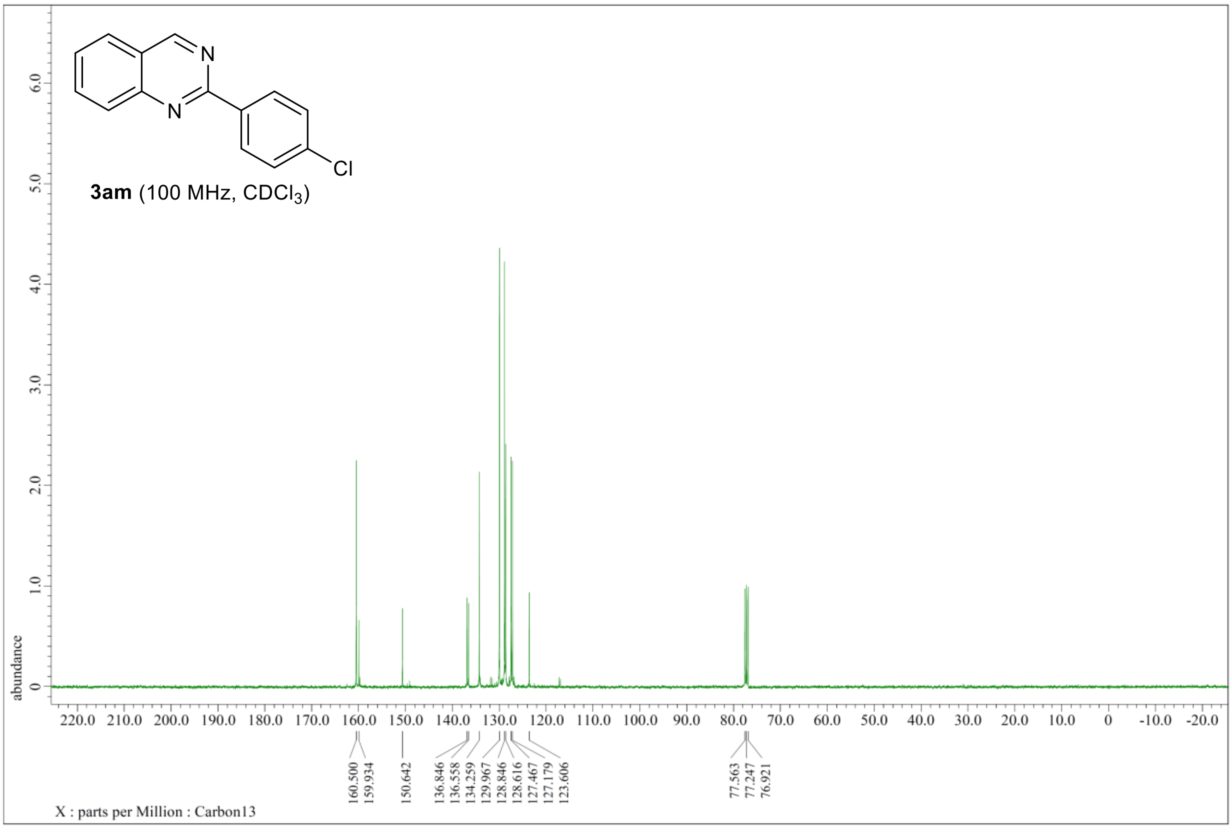

Figure S14: Copies of ^{1}H and $^{13}C\{^{1}H\}$ NMR spectra of compound **3an**

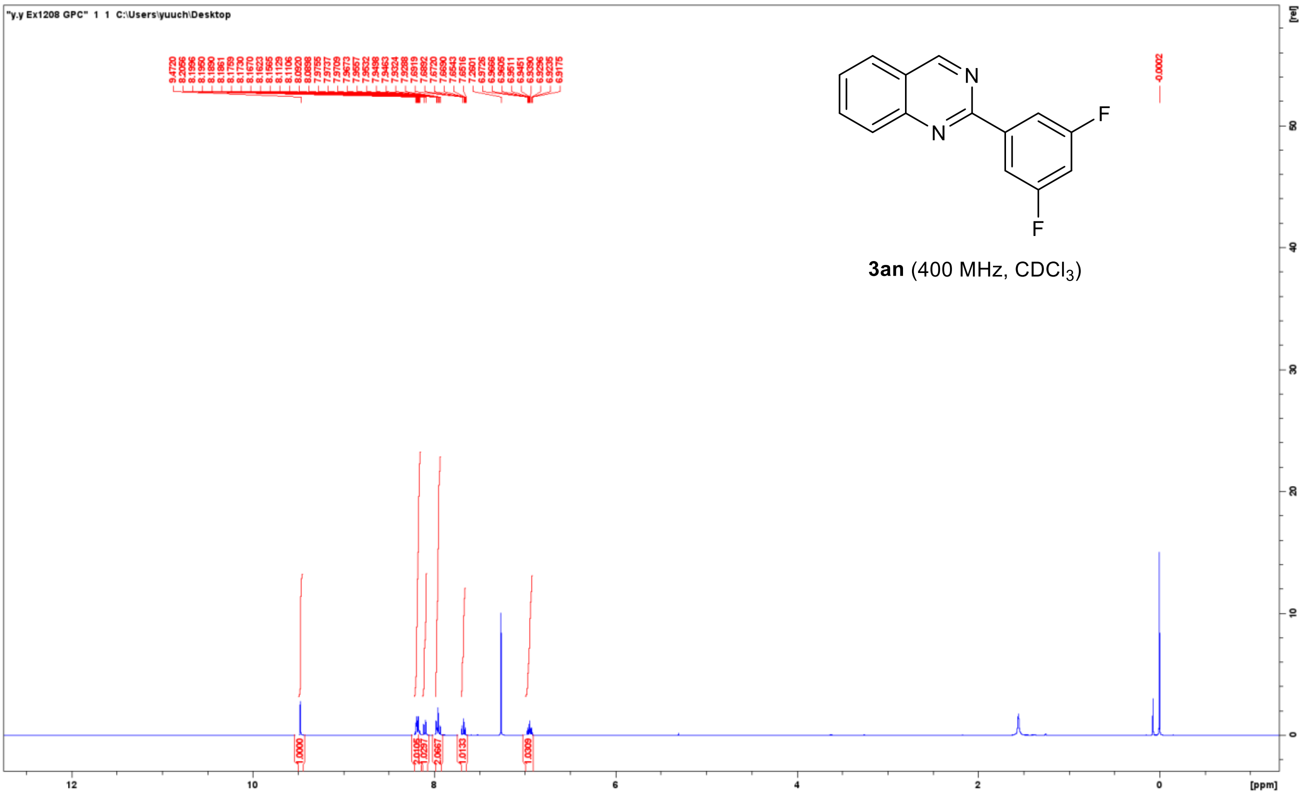

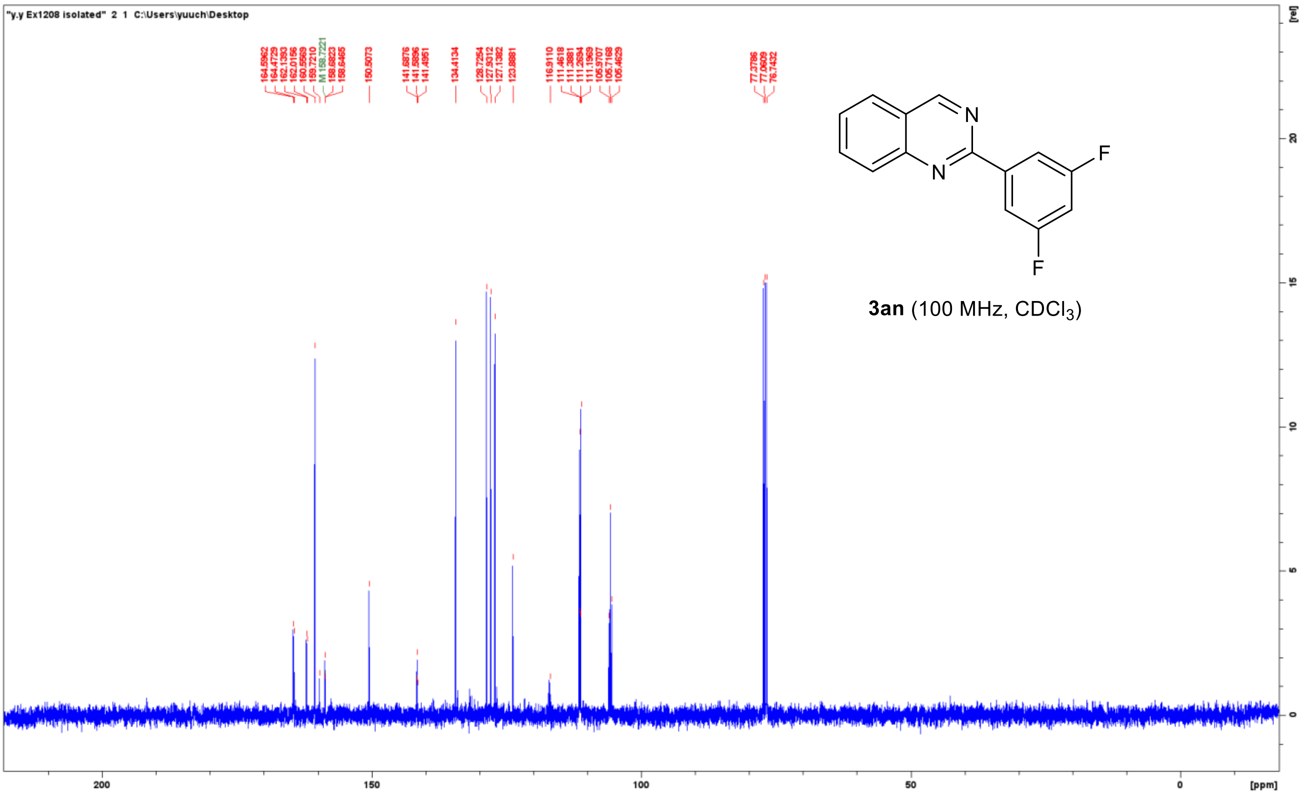

Figure S15: Copies of ^{1}H and ^{13}C{^{1}H} NMR spectra of compound **3ao**

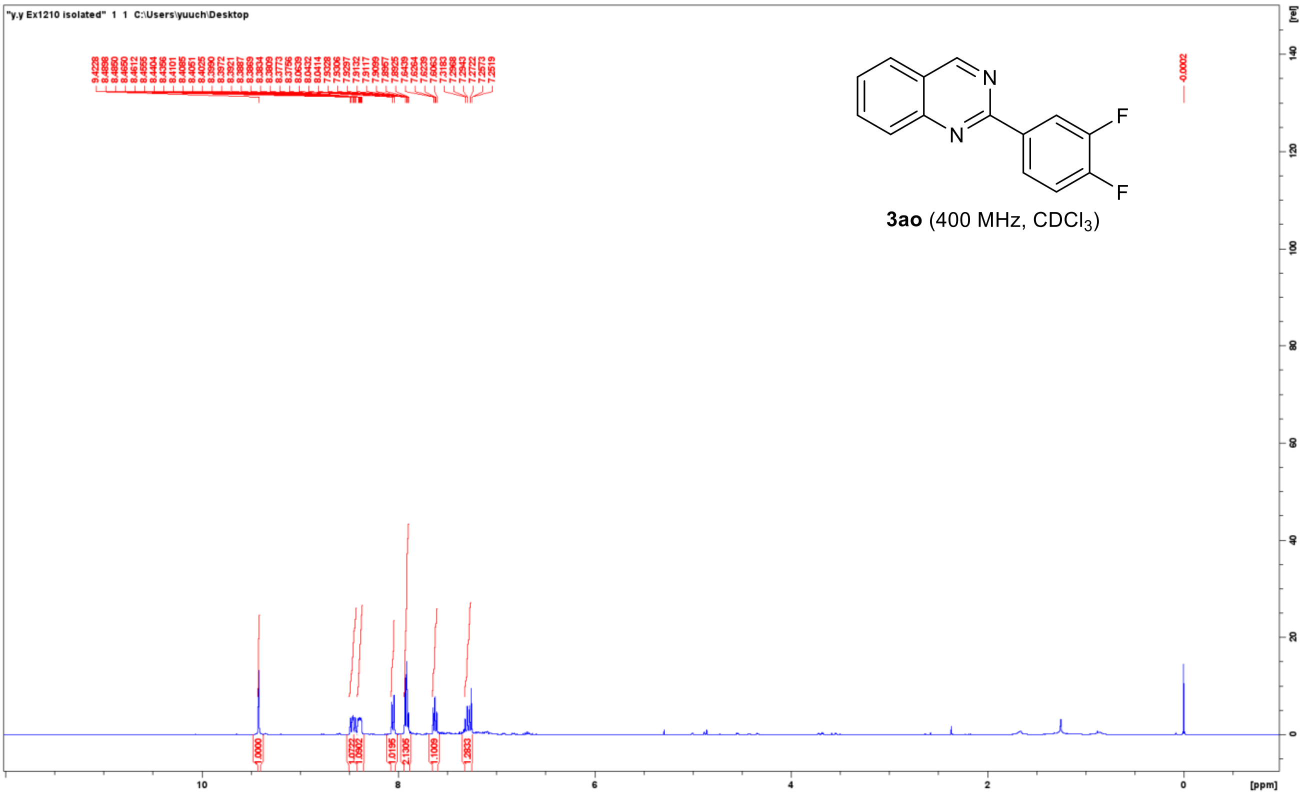

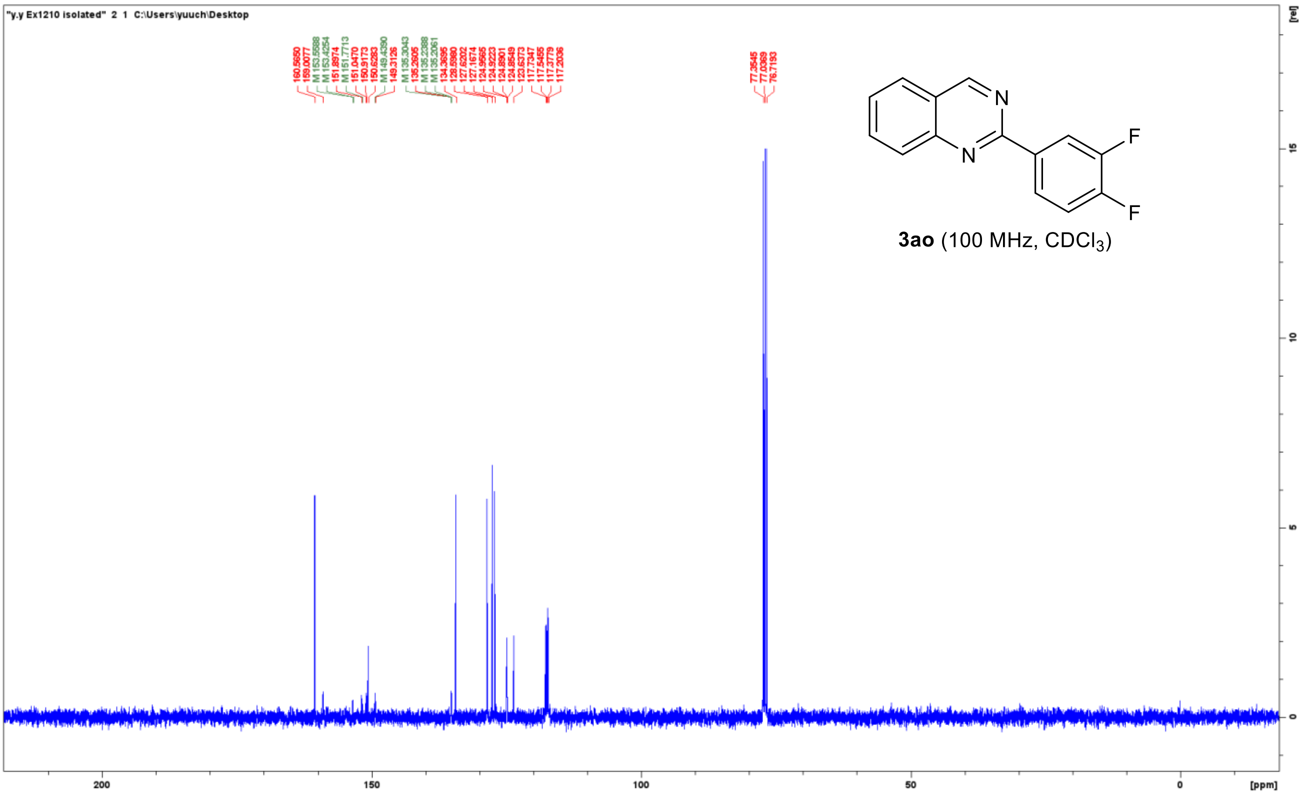

Figure S16: Copies of ^{1}H and $^{13}C\{^{1}H\}$ NMR spectra of compound **3ap**

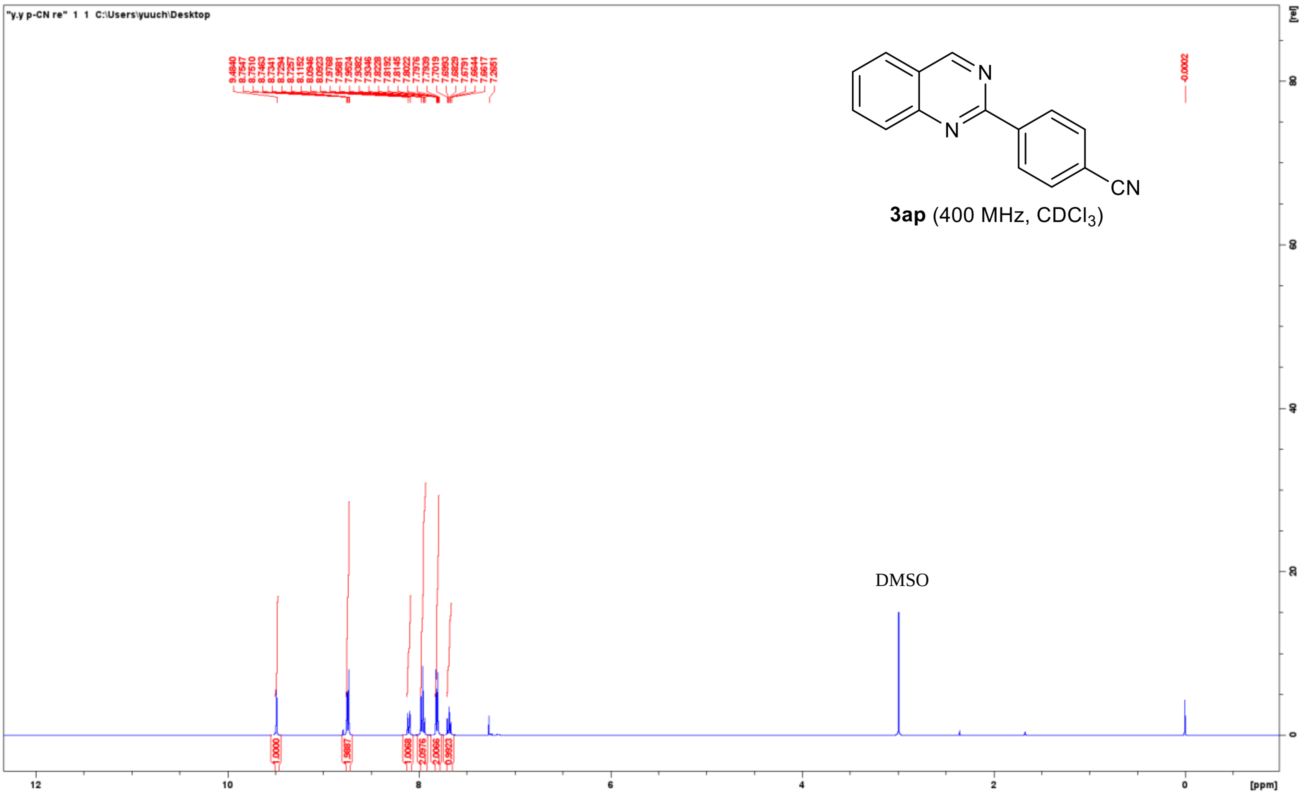

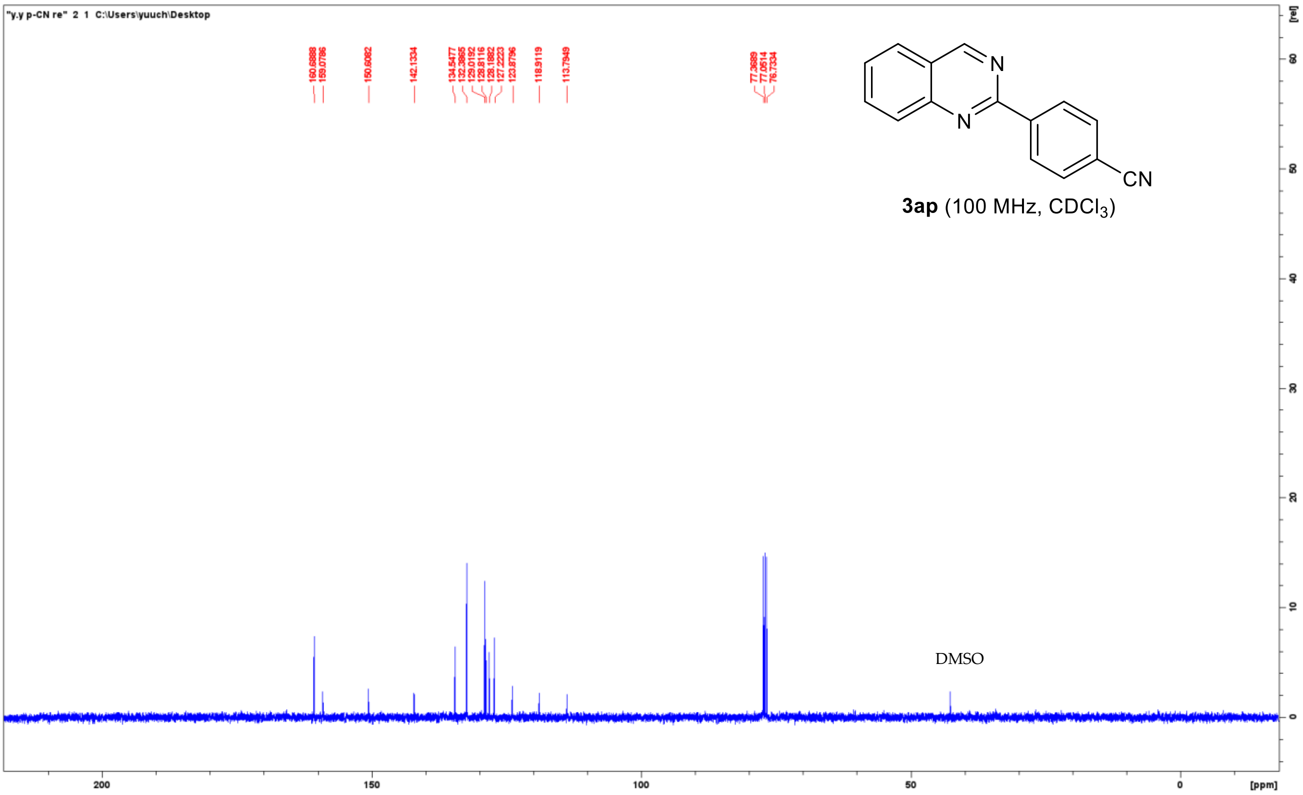

Figure S17: Copies of ^{1}H and $^{13}C\{^{1}H\}$ NMR spectra of compound **3aq**

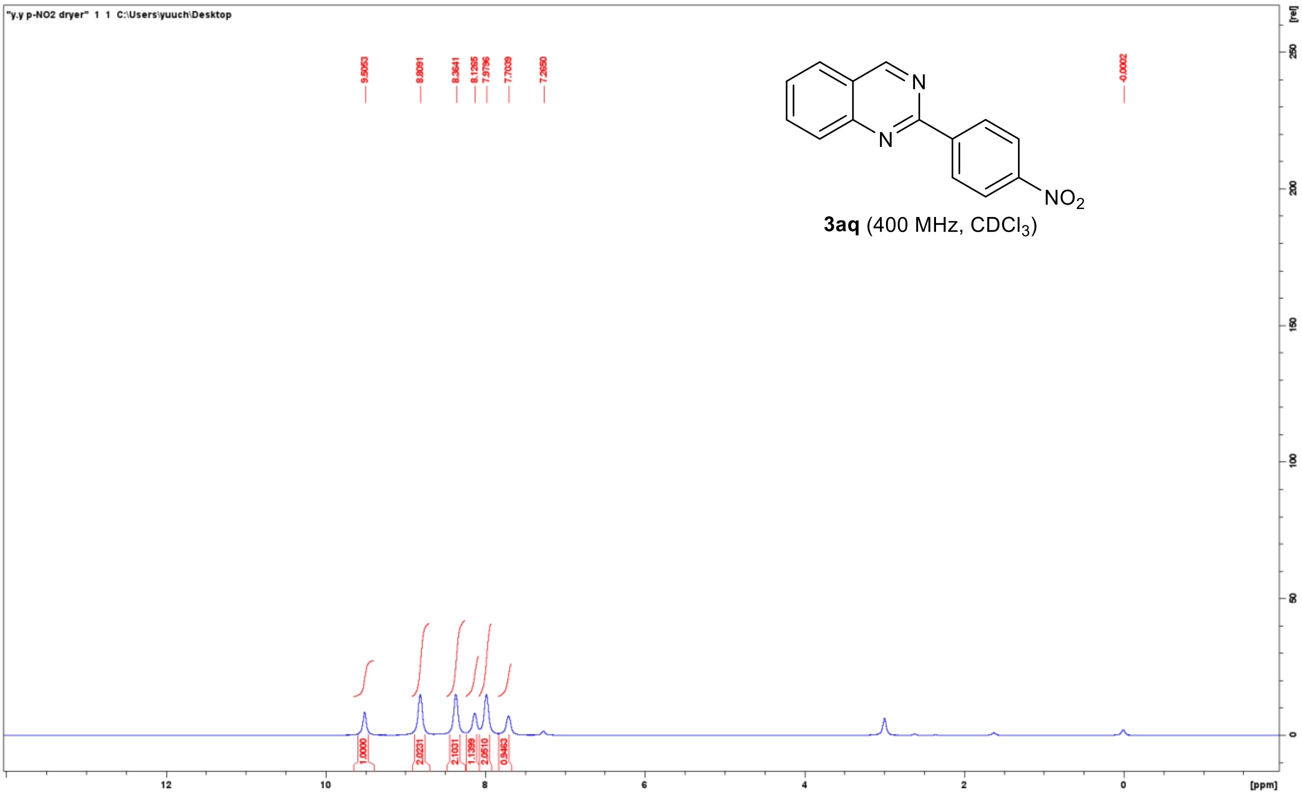

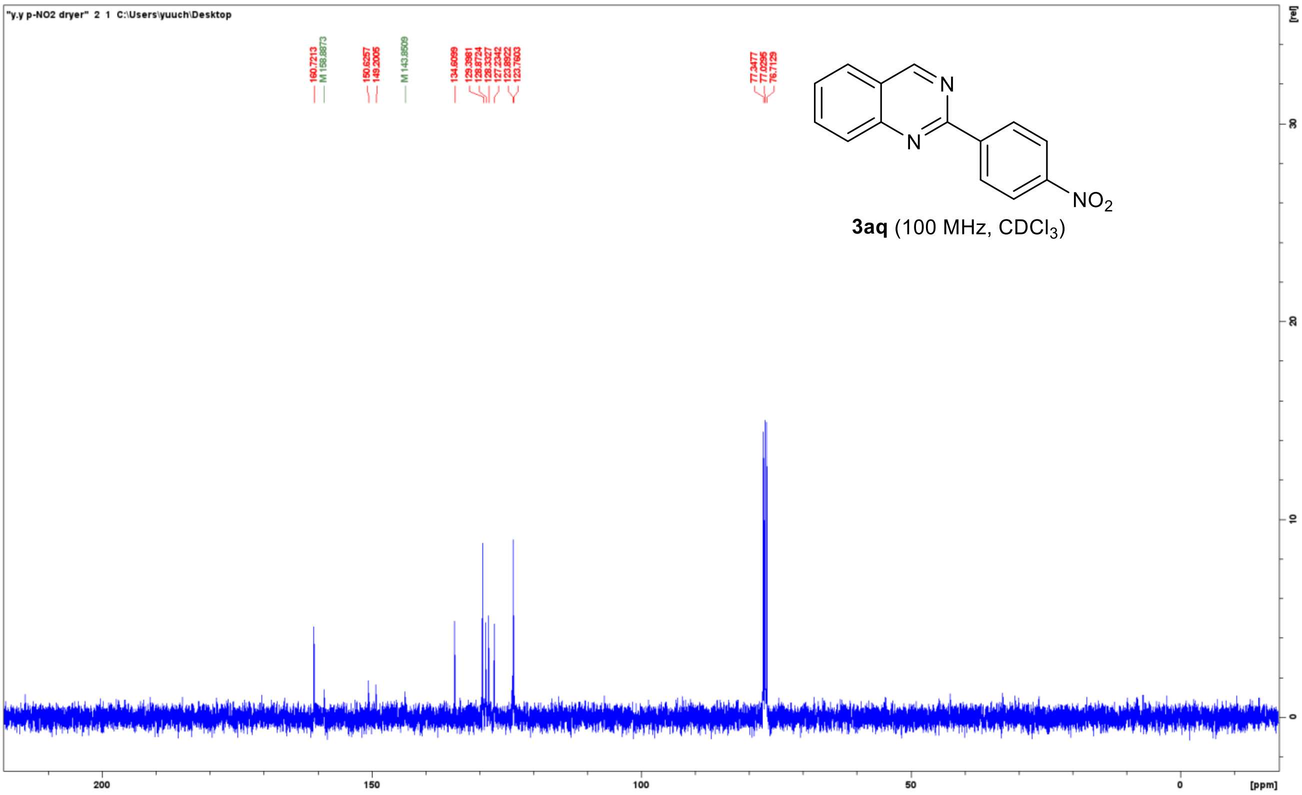

Figure S18: Copies of ^{1}H and $^{13}C\{^{1}H\}$ NMR spectra of compound **3ar**

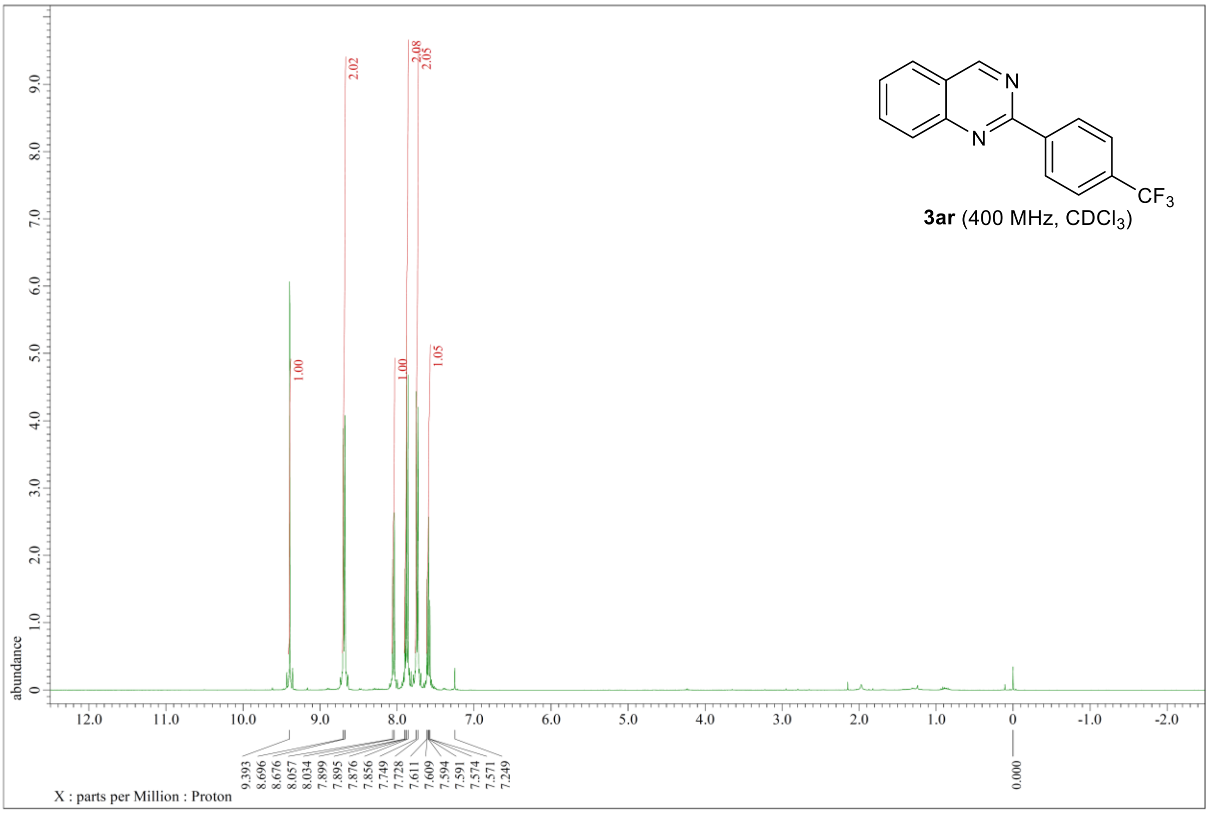

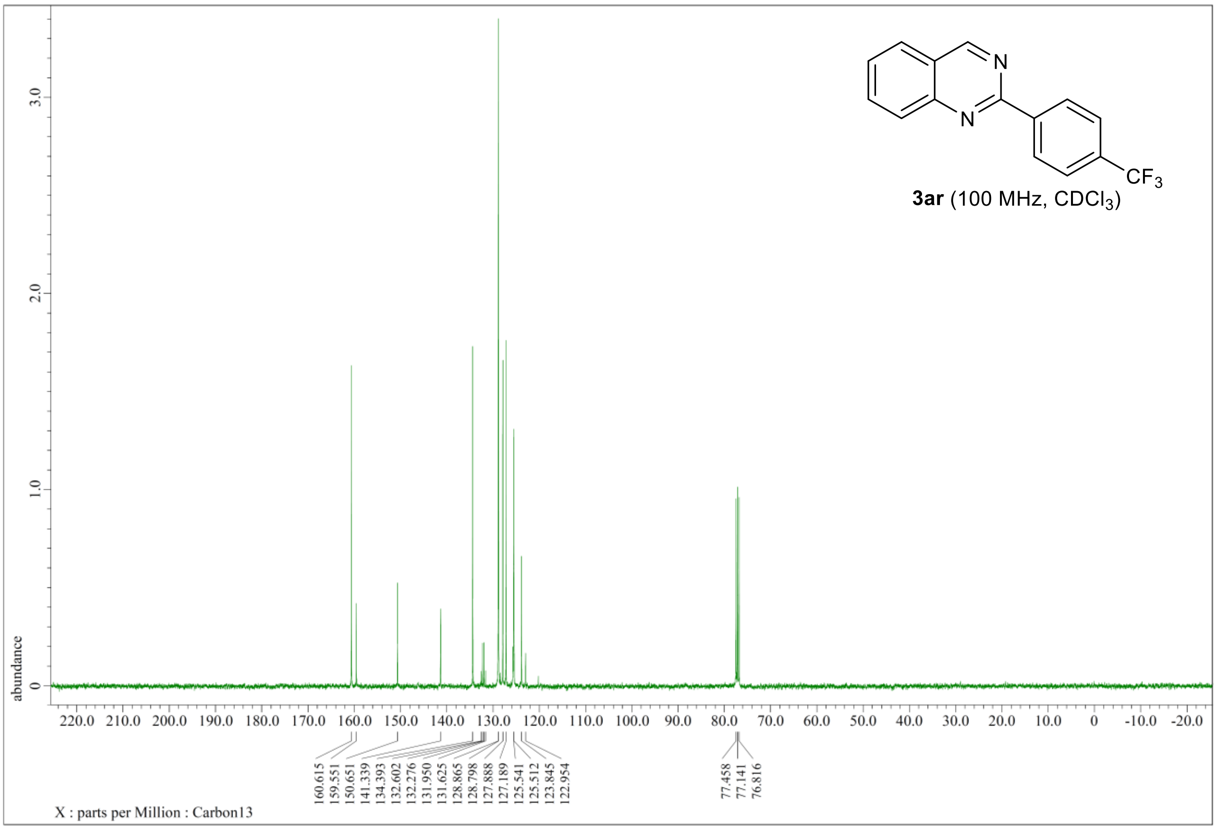

Figure S19: Copies of ^{1}H and $^{13}C\{^{1}H\}$ NMR spectra of compound **3as**

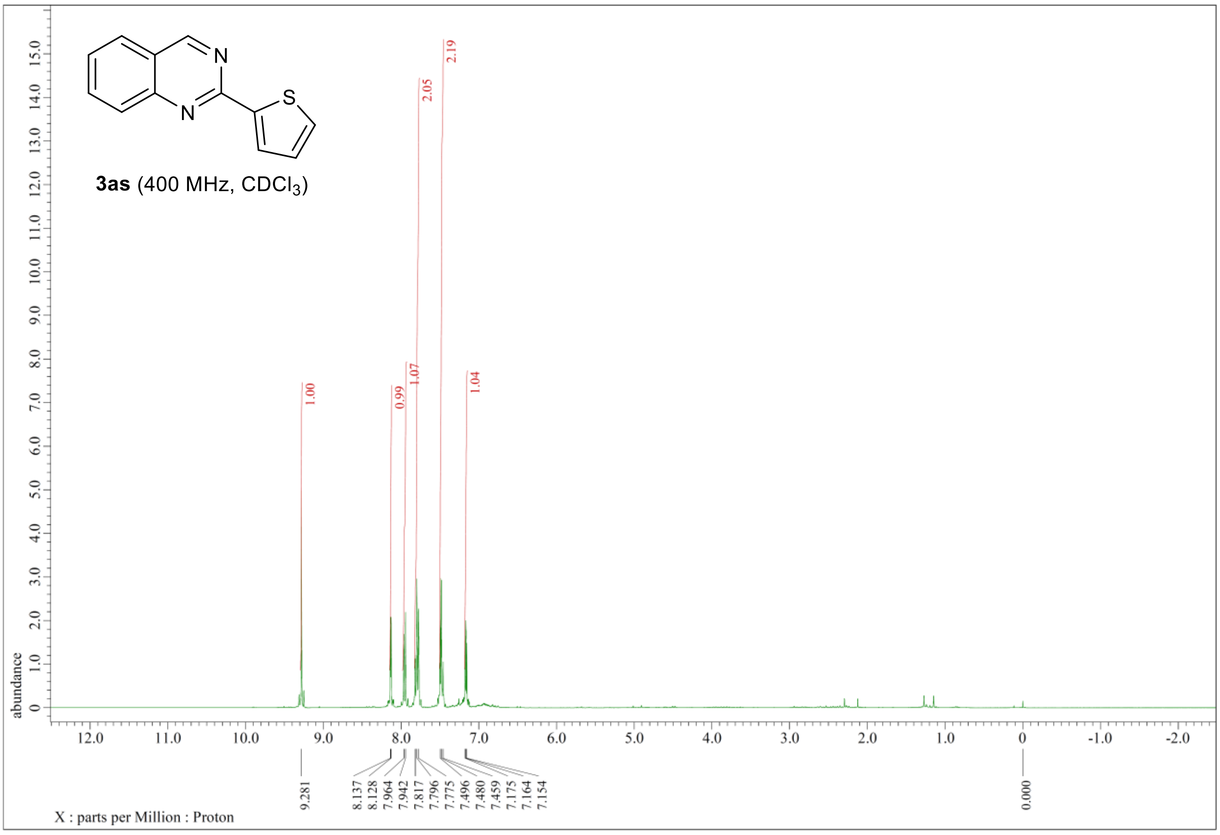

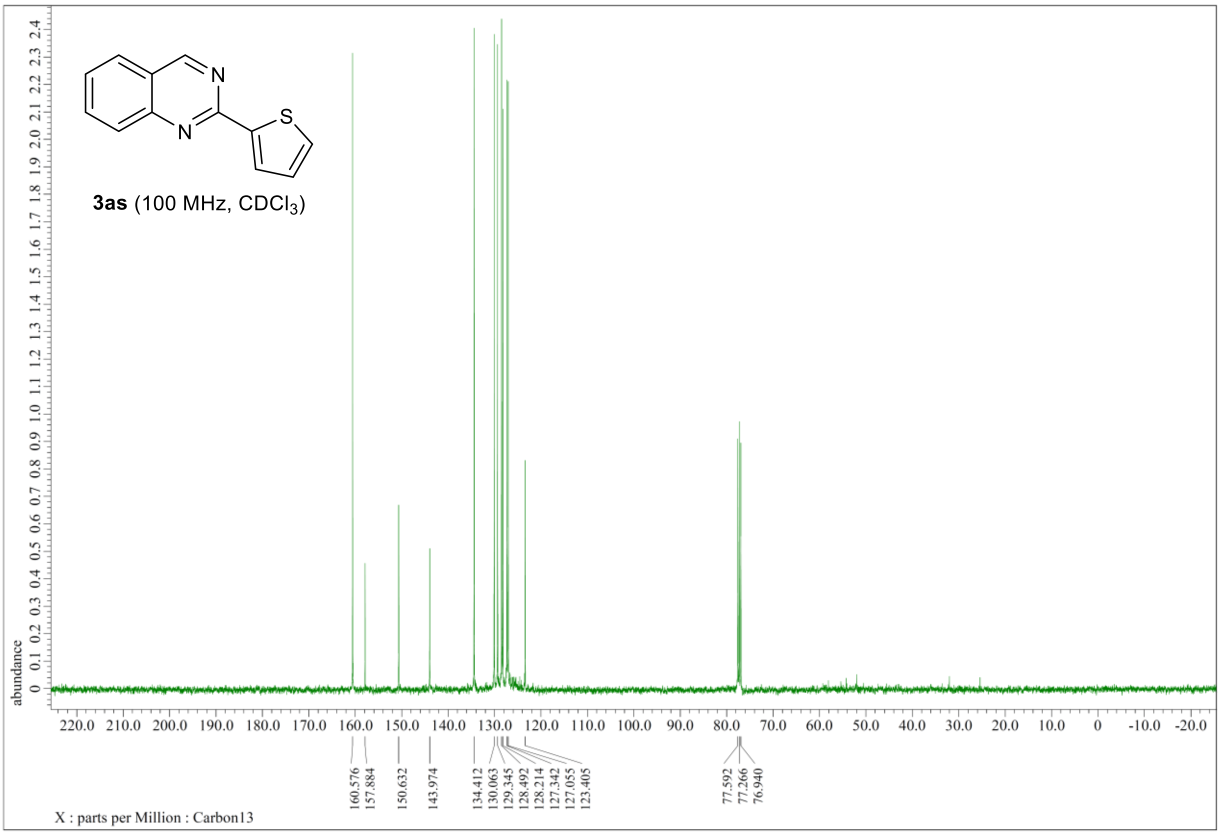

Figure S20: Copies of ^{1}H and $^{13}C\{^{1}H\}$ NMR spectra of compound **3at**

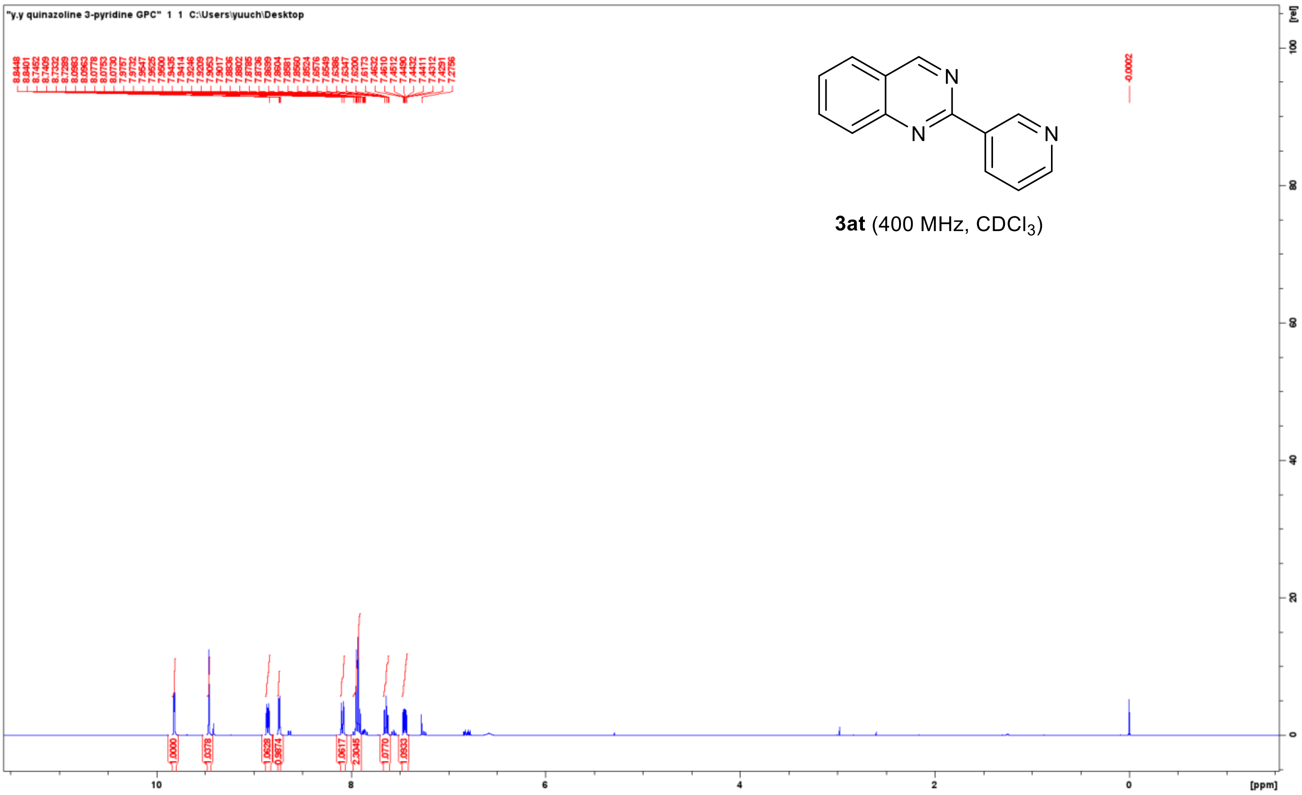

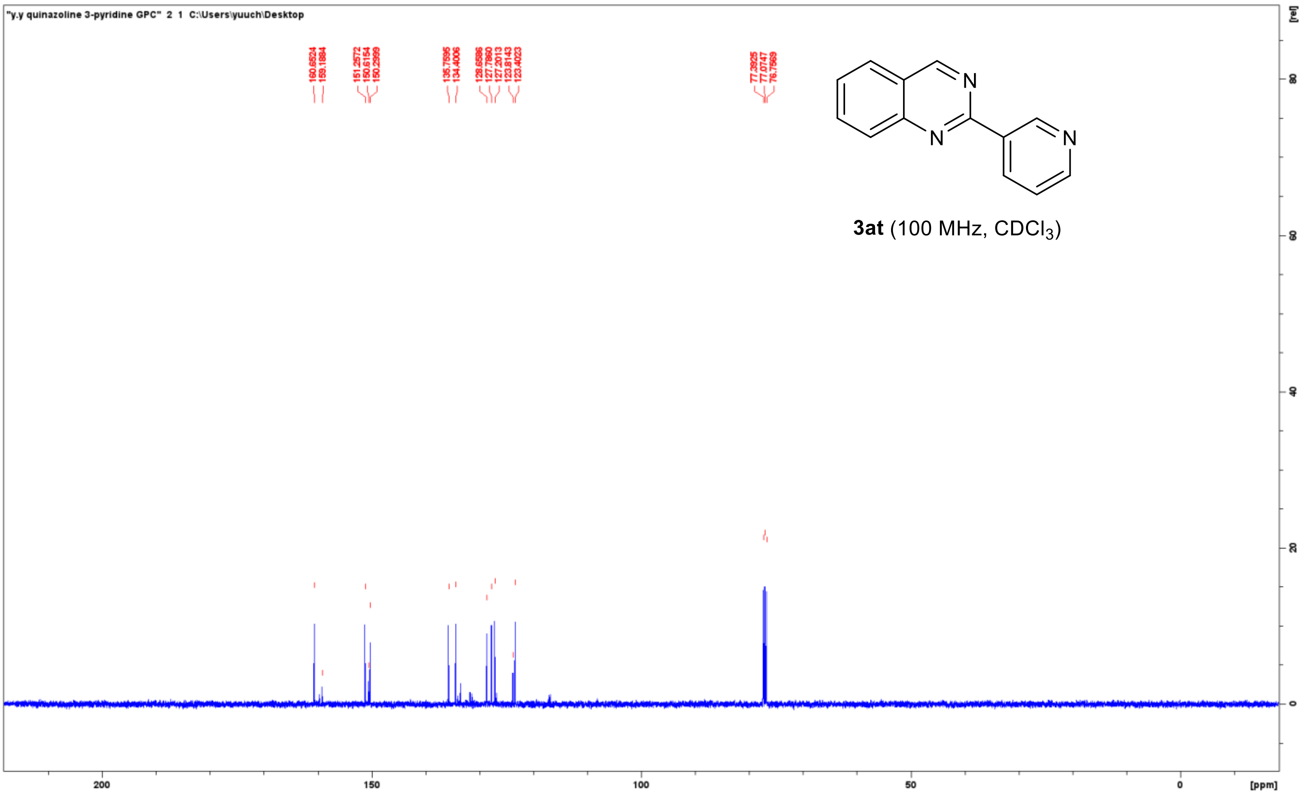

Figure S21: Copies of ^{1}H and $^{13}C\{^{1}H\}$ NMR spectra of compound **3ba**

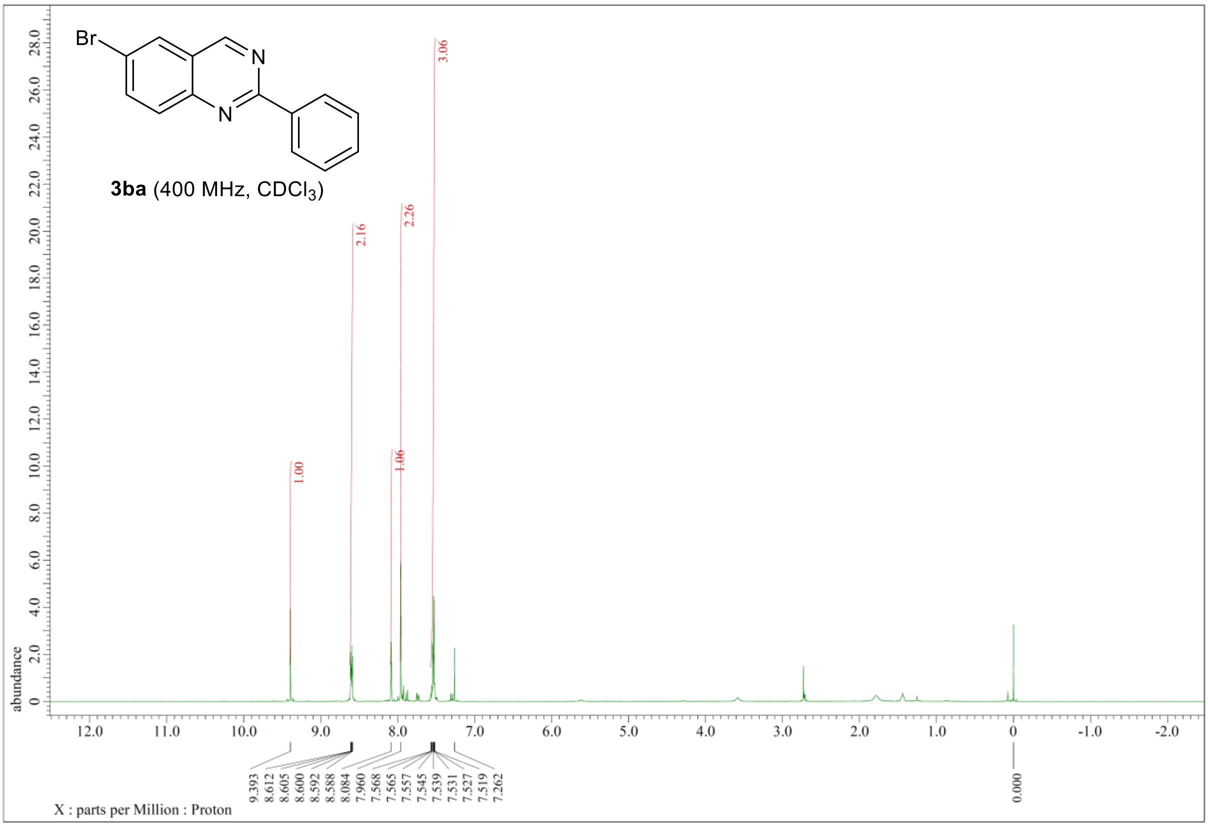

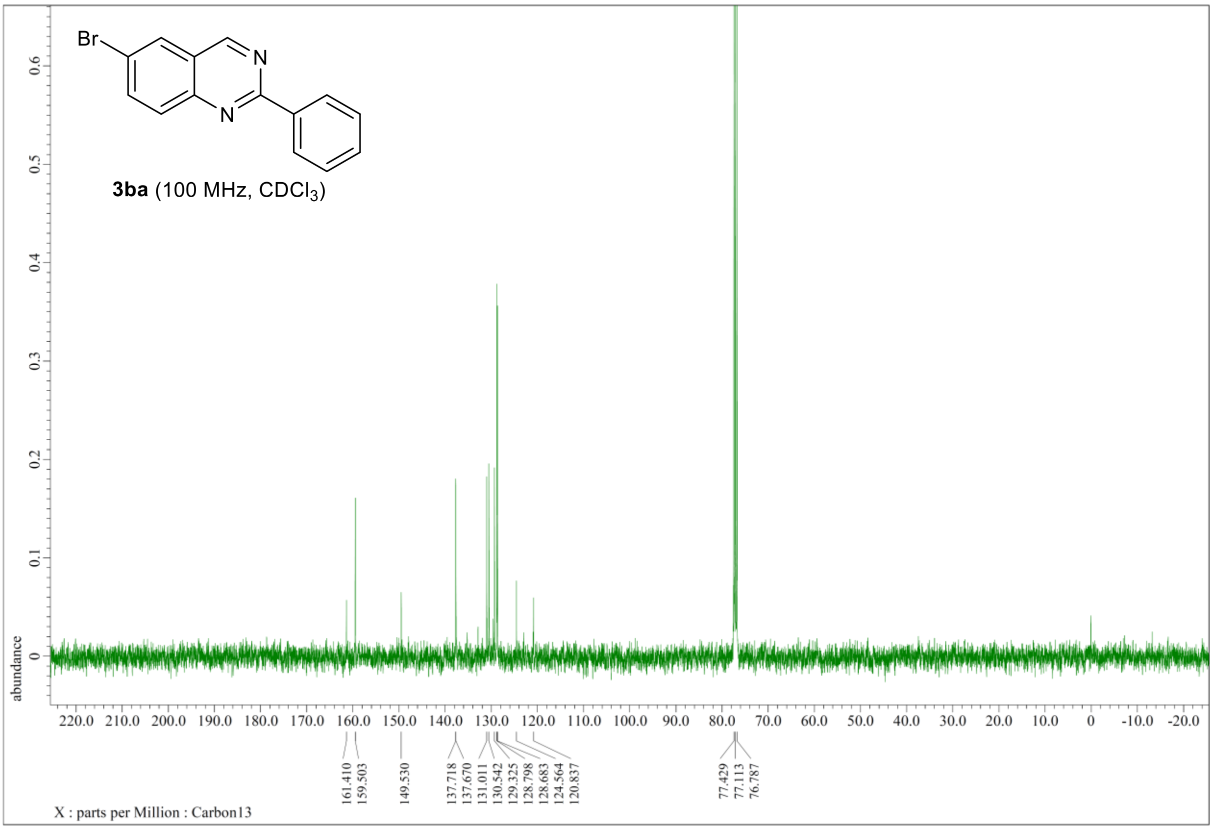

Figure S22: Copies of ^{1}H and ^{13}C{^{1}H} NMR spectra of compound **4a**

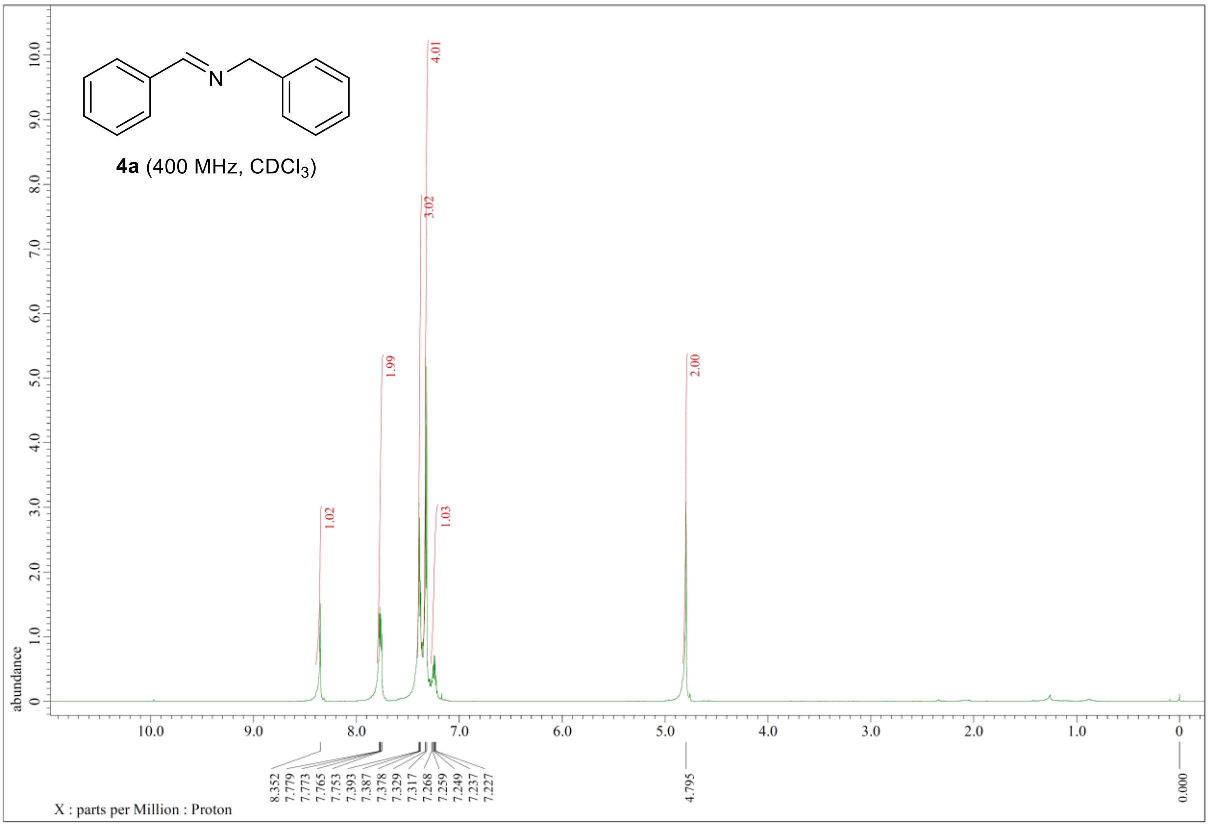

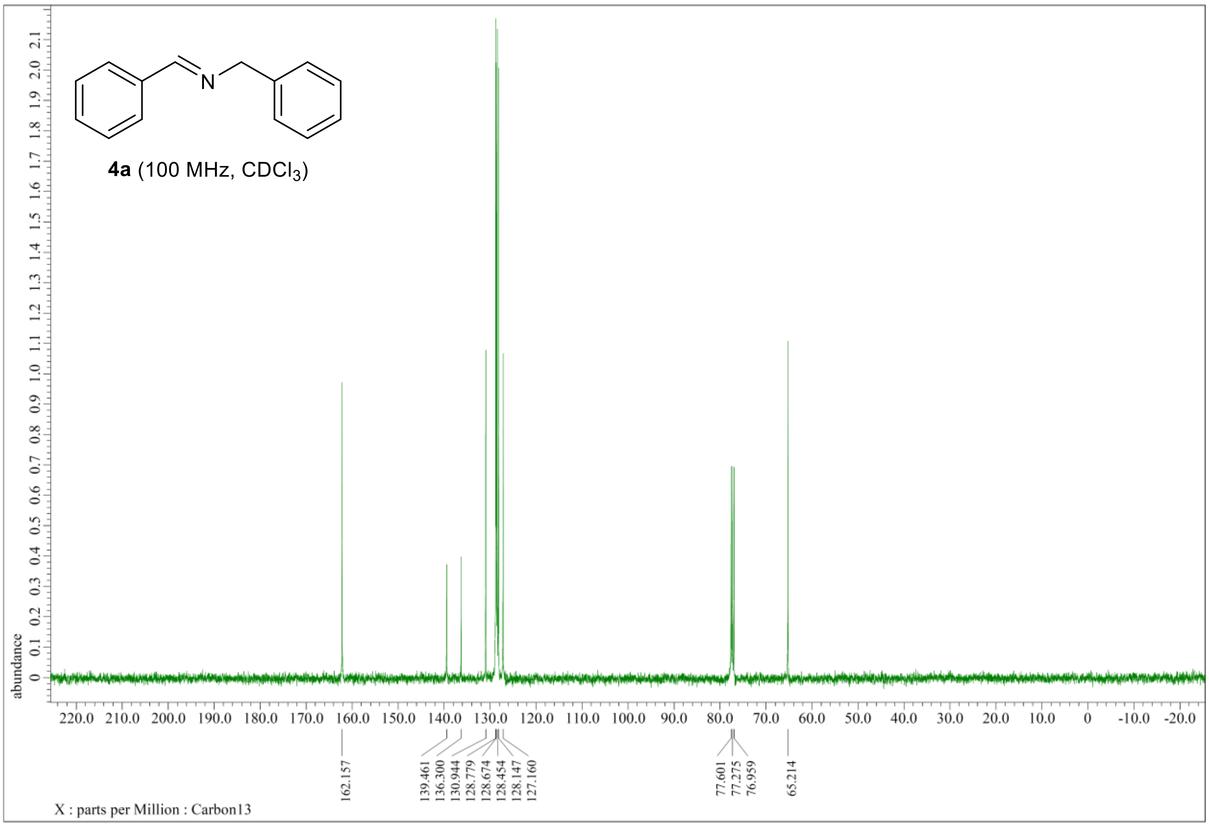

Figure S23: Copies of ^{1}H and ^{13}C{^{1}H} NMR spectra of compound **4b**

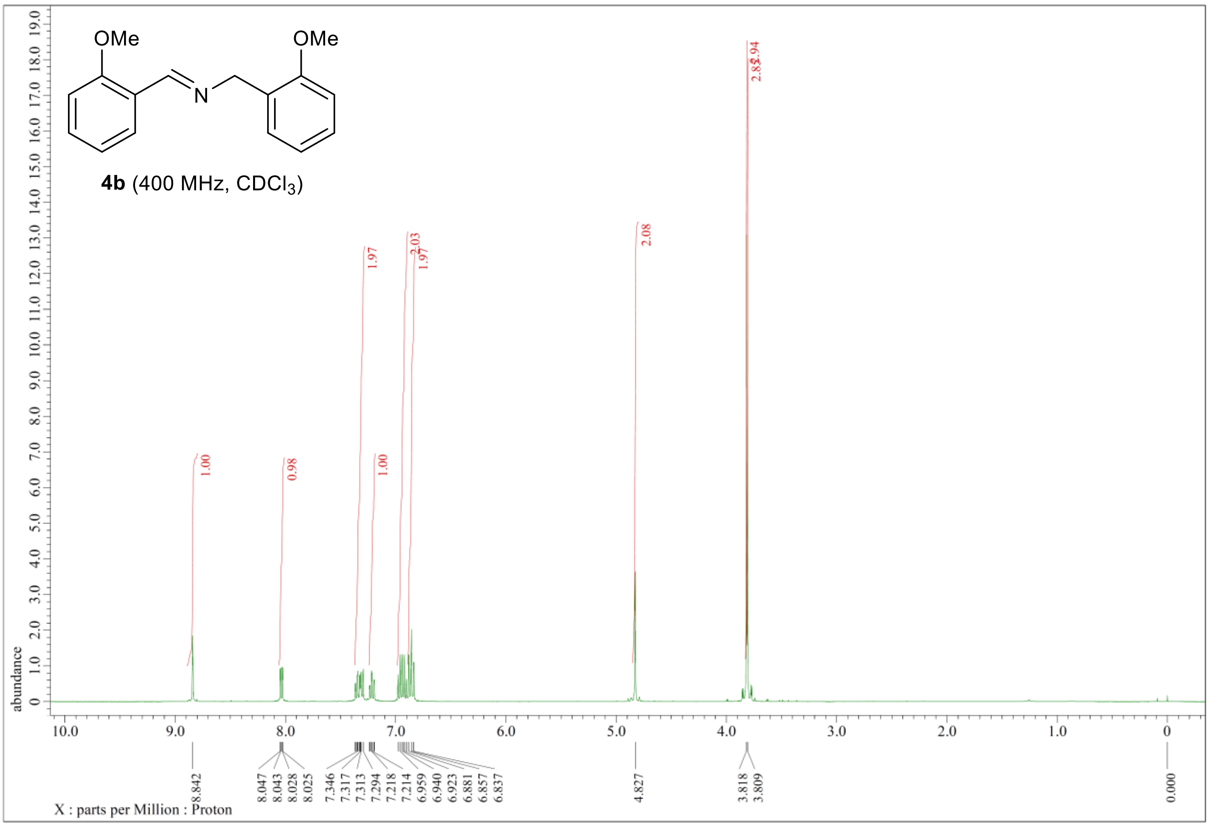

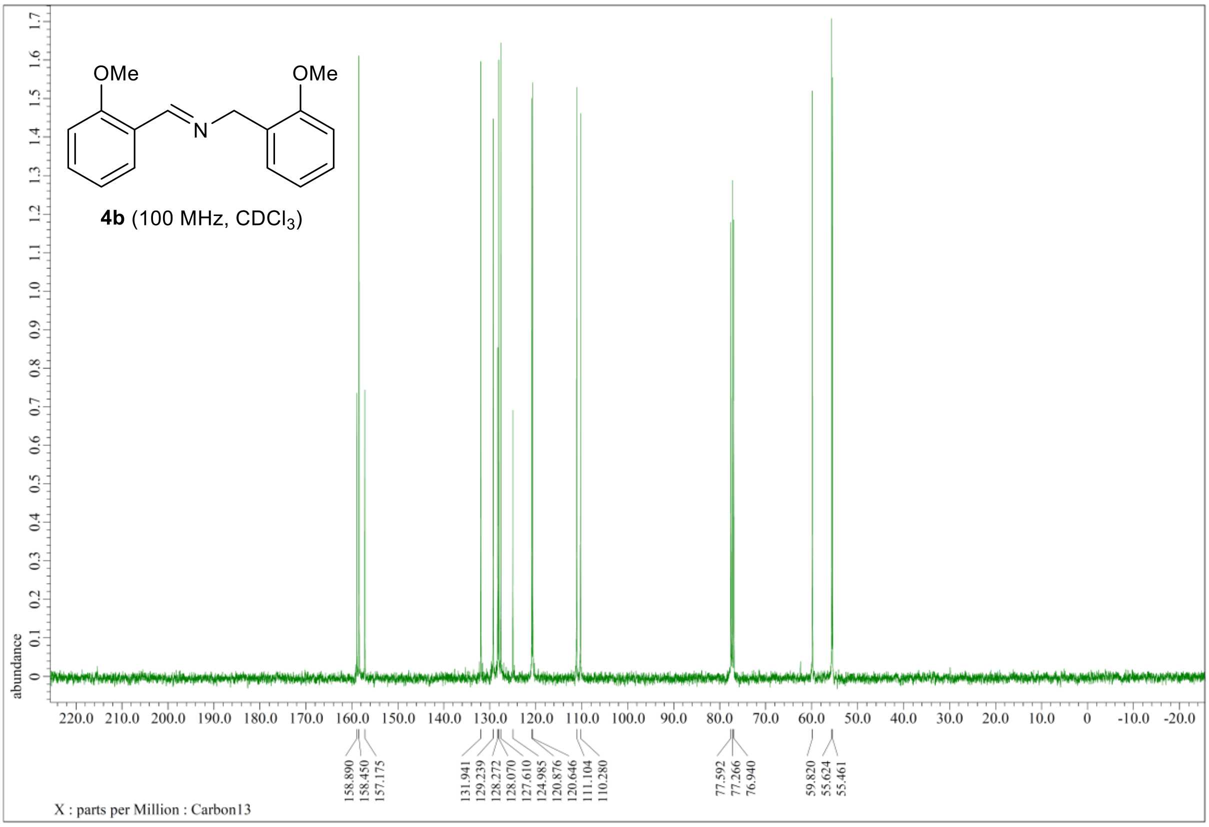

Figure S24: Copies of ^{1}H and $^{13}C\{^{1}H\}$ NMR spectra of compound **4c**

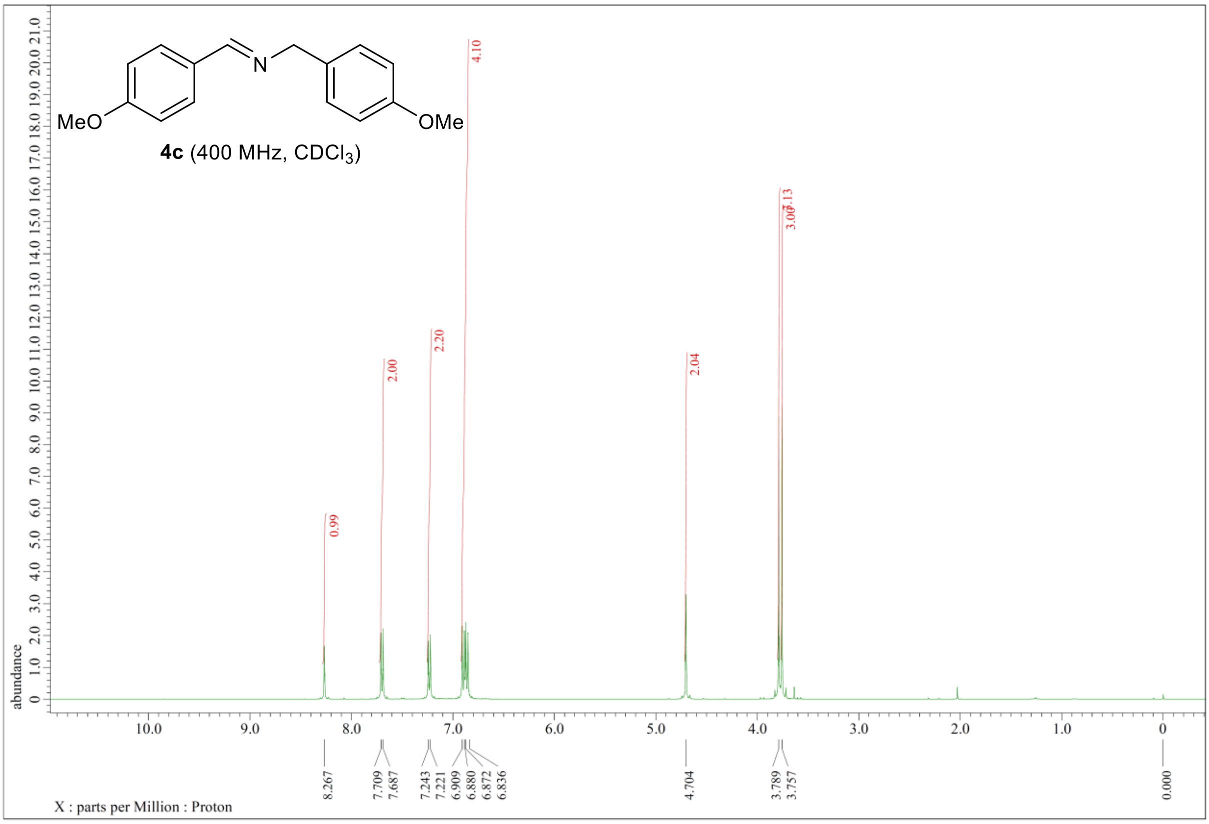

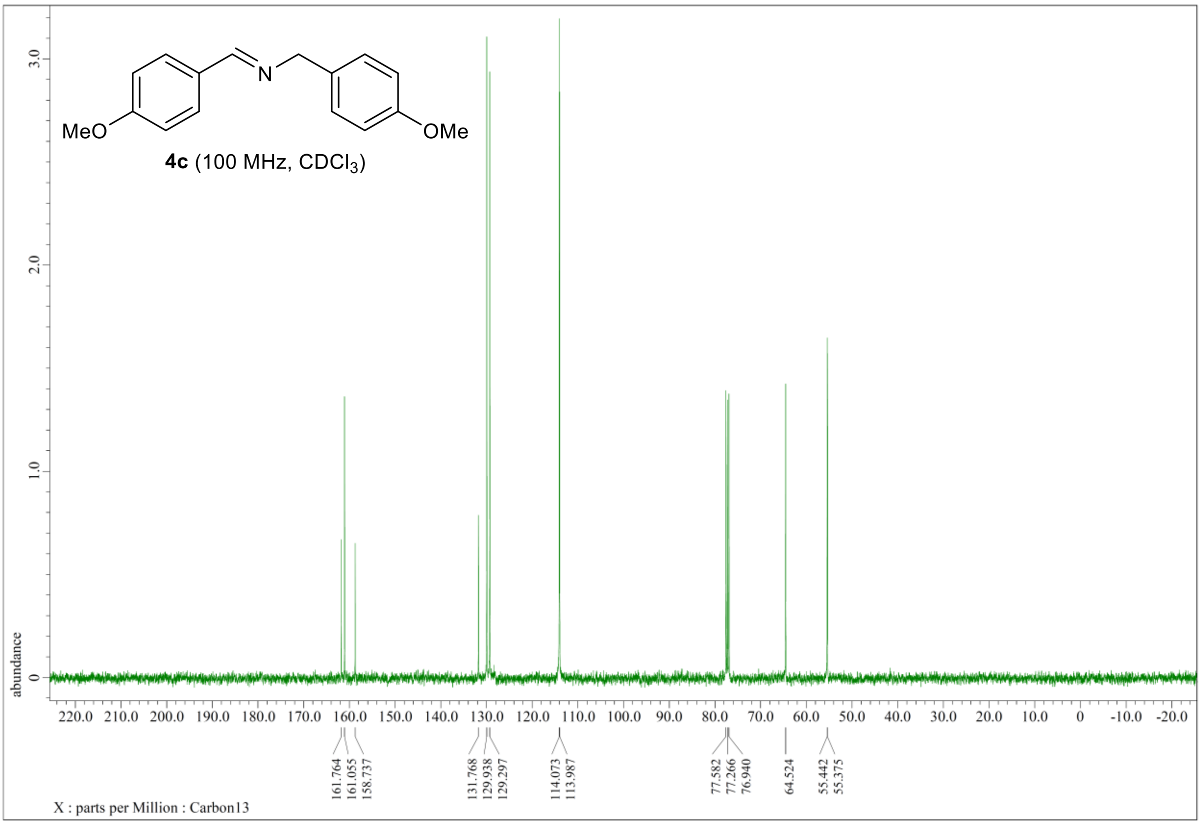

Figure S25: Copies of ^{1}H and $^{13}C\{^{1}H\}$ NMR spectra of compound **4d**

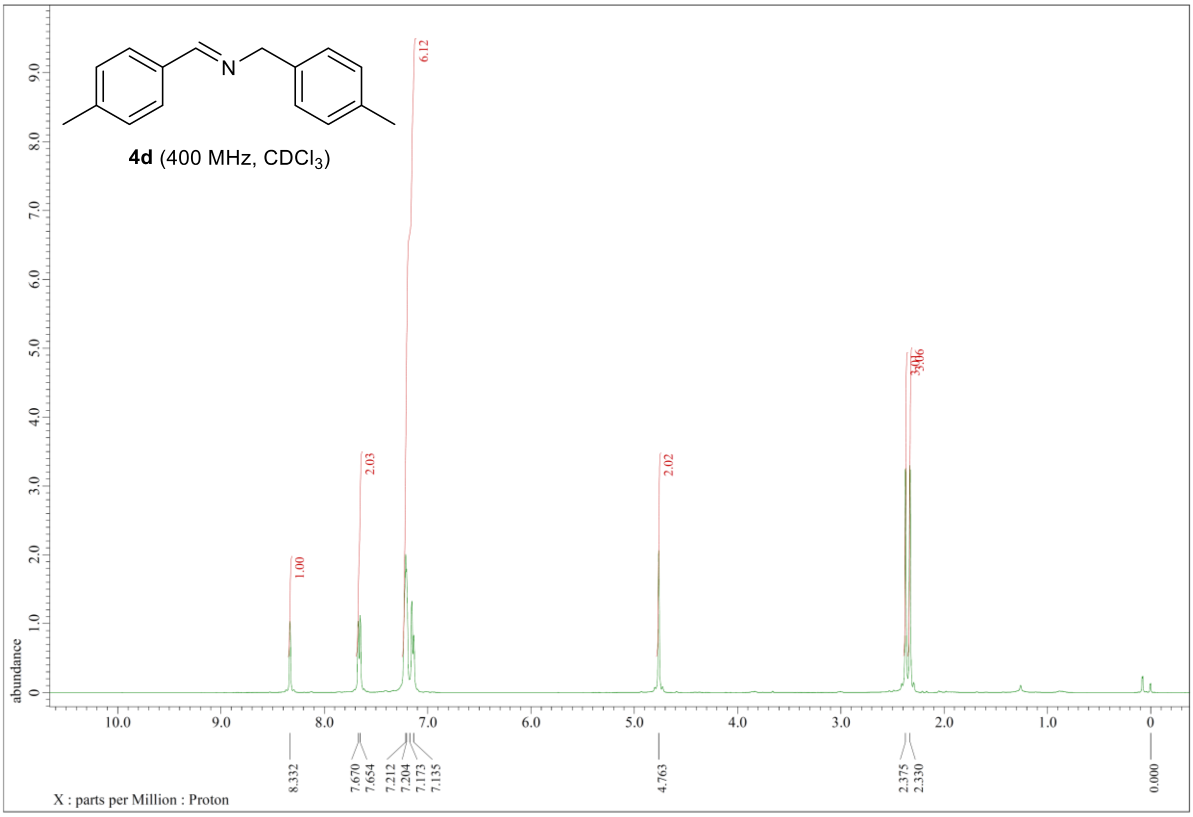

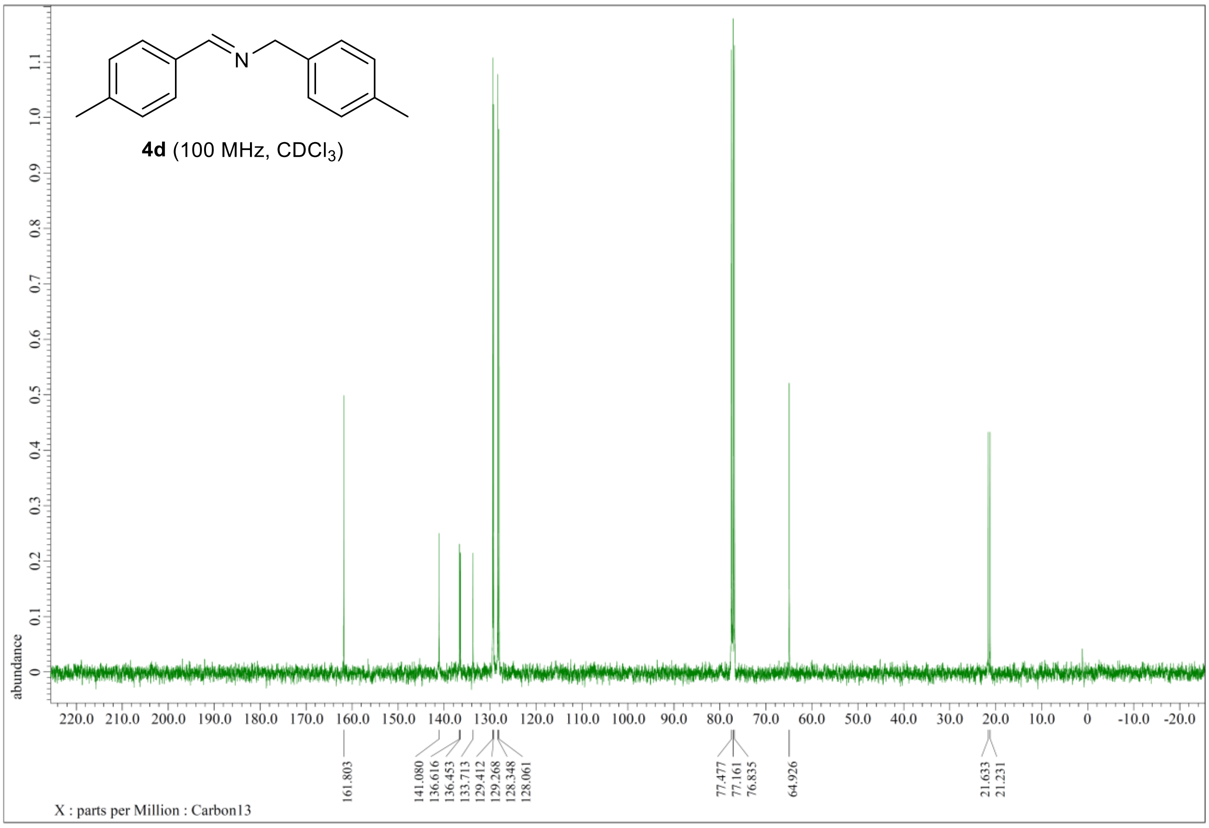

Figure S26: Copies of ^{1}H and ^{13}C{^{1}H} NMR spectra of compound **6a**

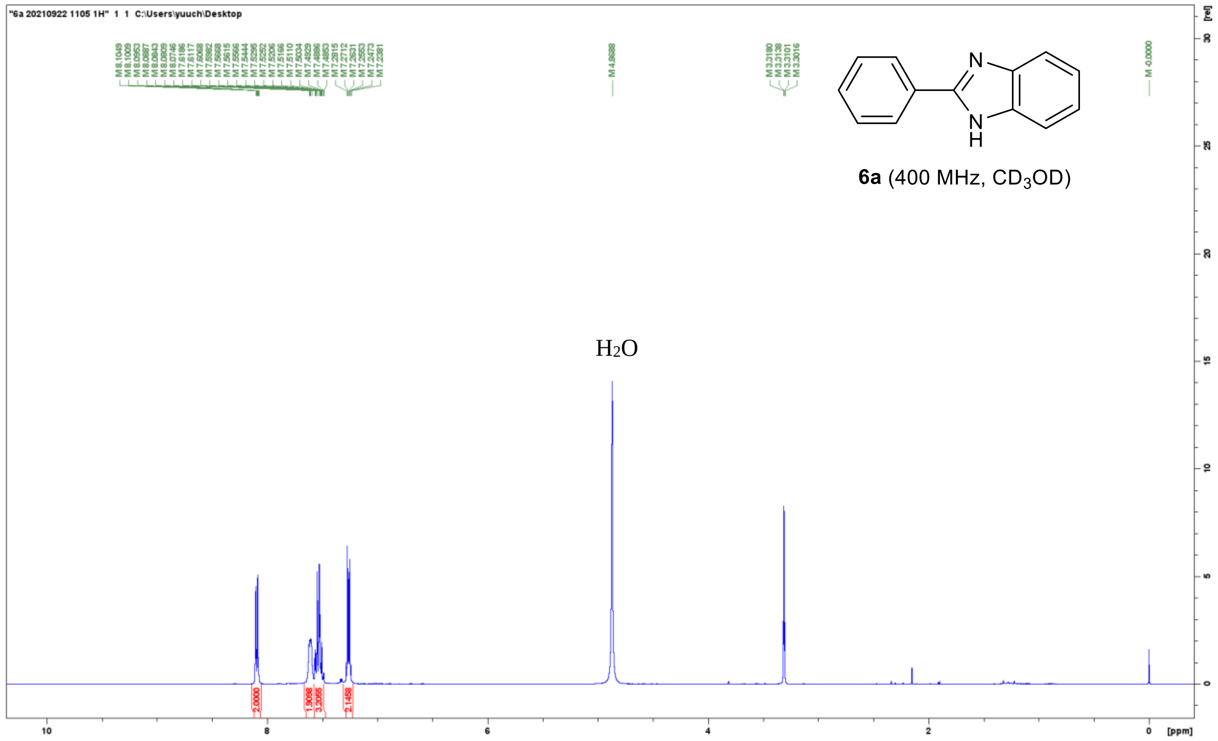

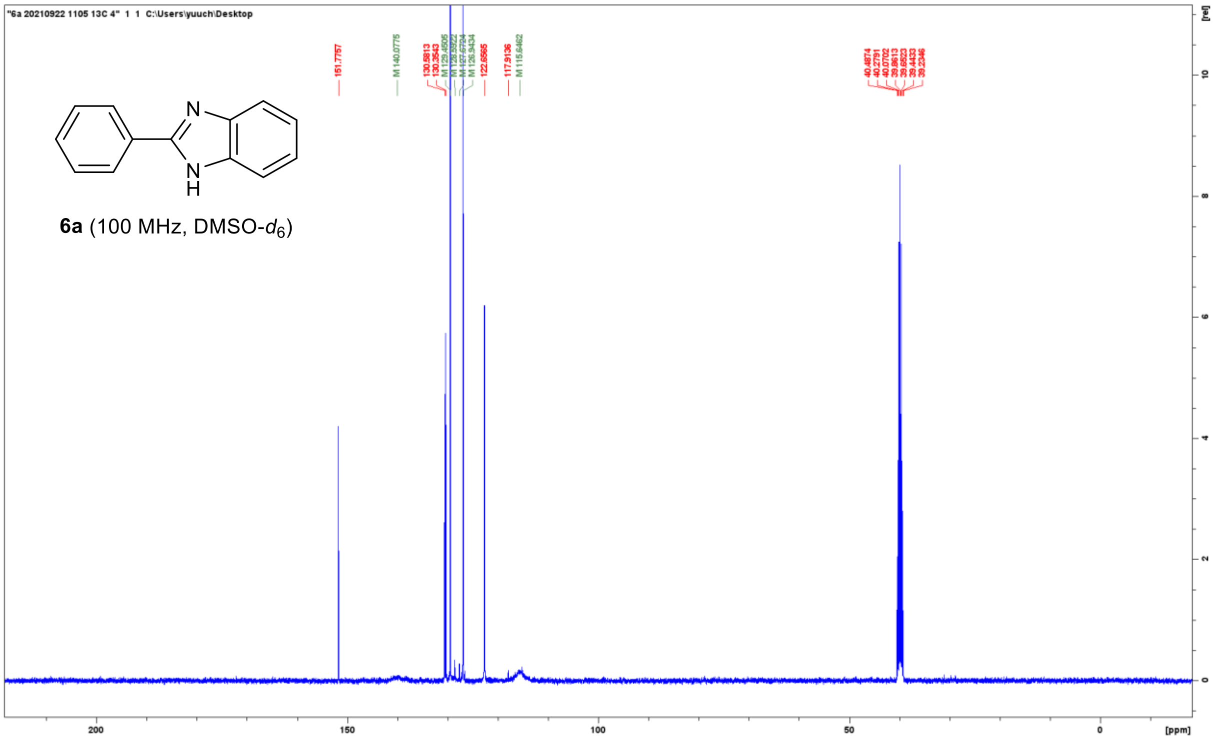

Figure S27: Copies of ^{1}H and ^{13}C{^{1}H} NMR spectra of compound **6b**

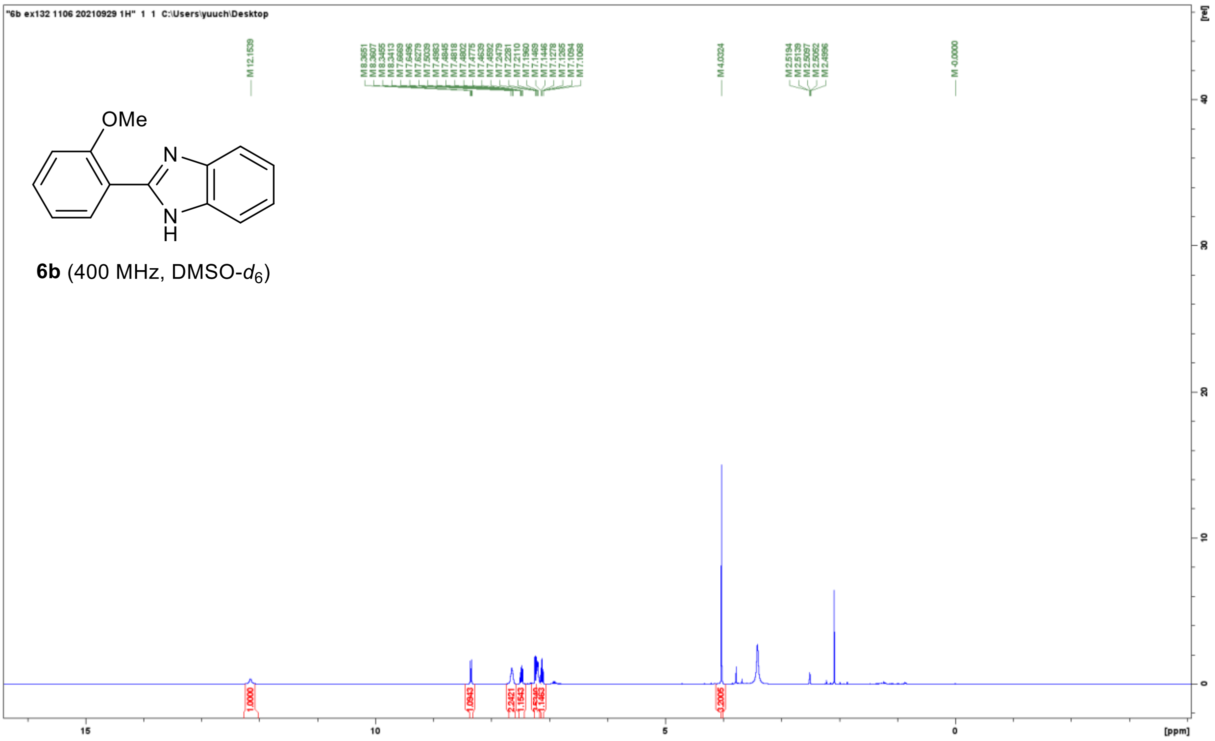

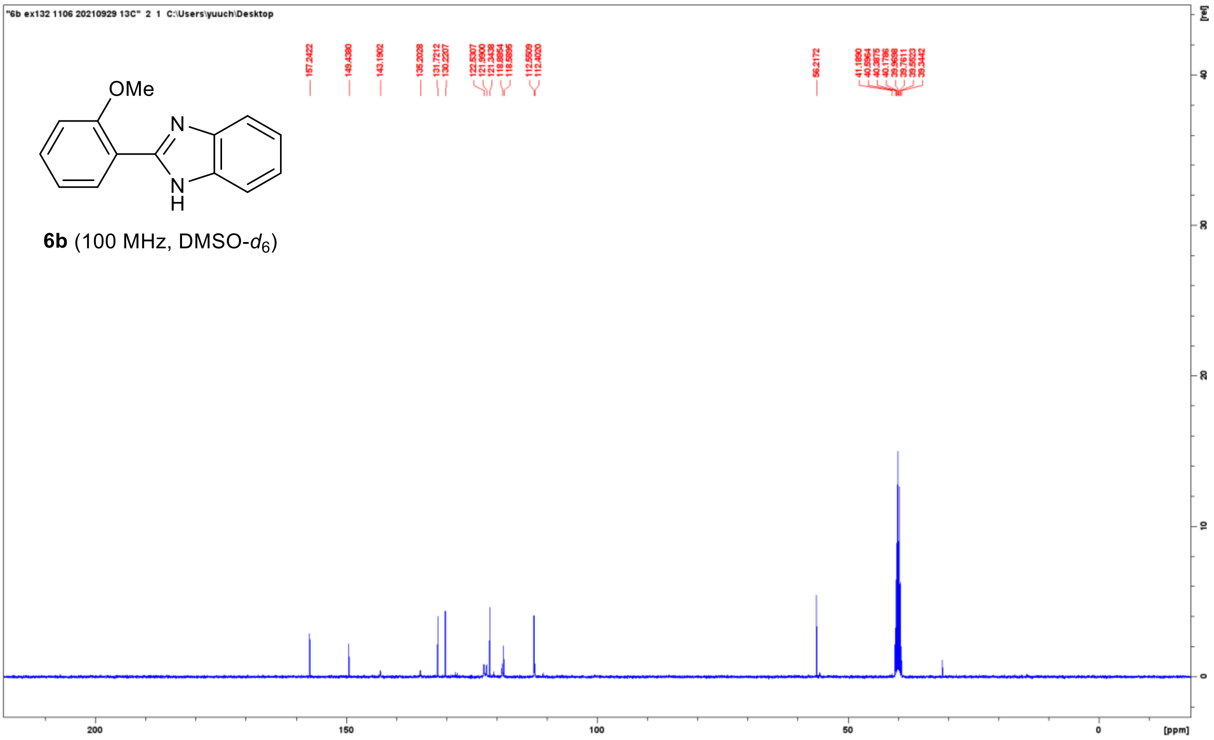

Figure S28: Copies of ^{1}H and ^{13}C{^{1}H} NMR spectra of compound **6c**

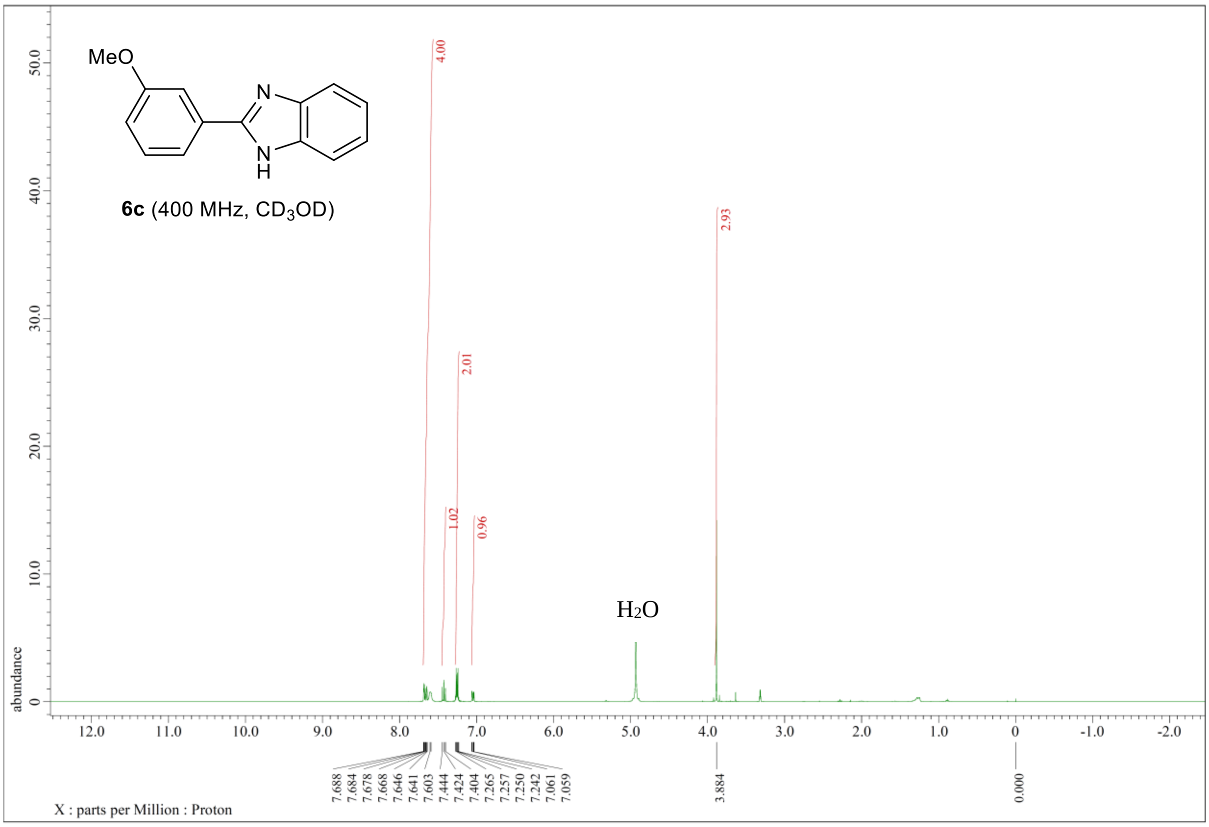

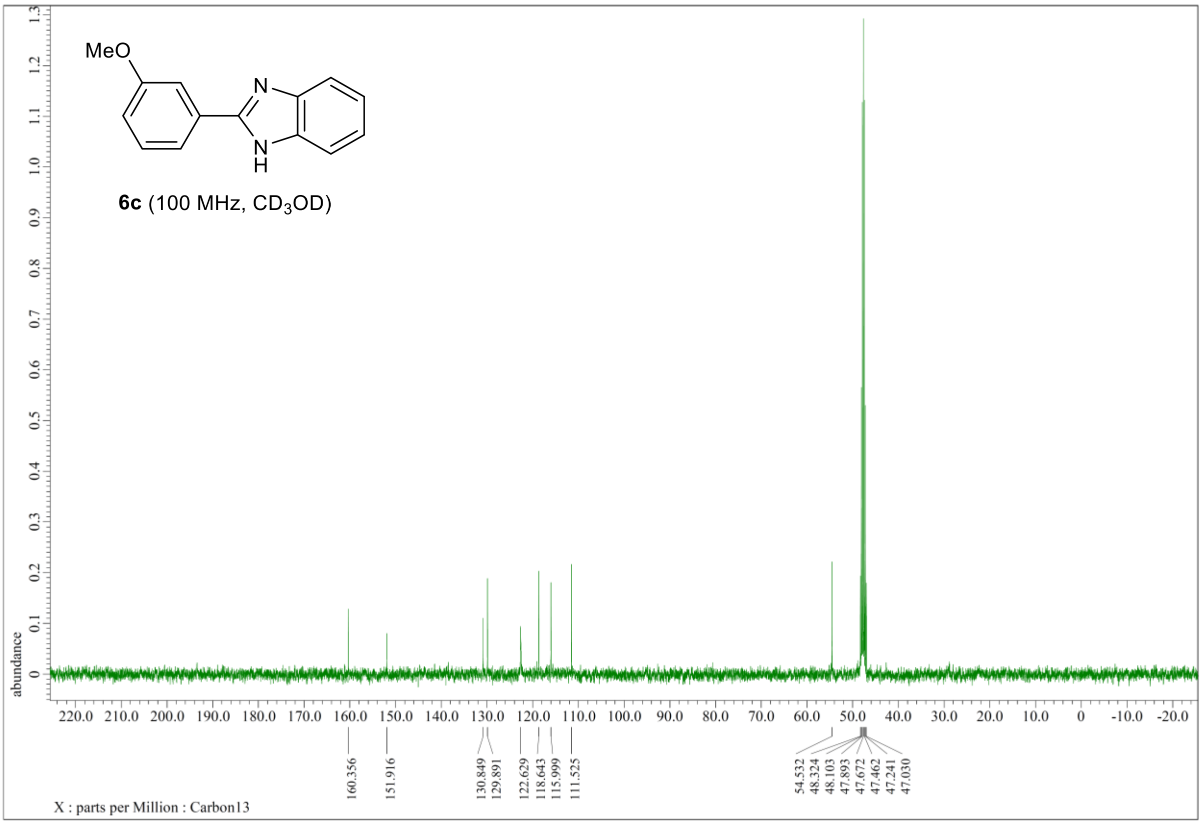

Figure S29: Copies of ^{1}H and $^{13}C\{^{1}H\}$ NMR spectra of compound **6d**

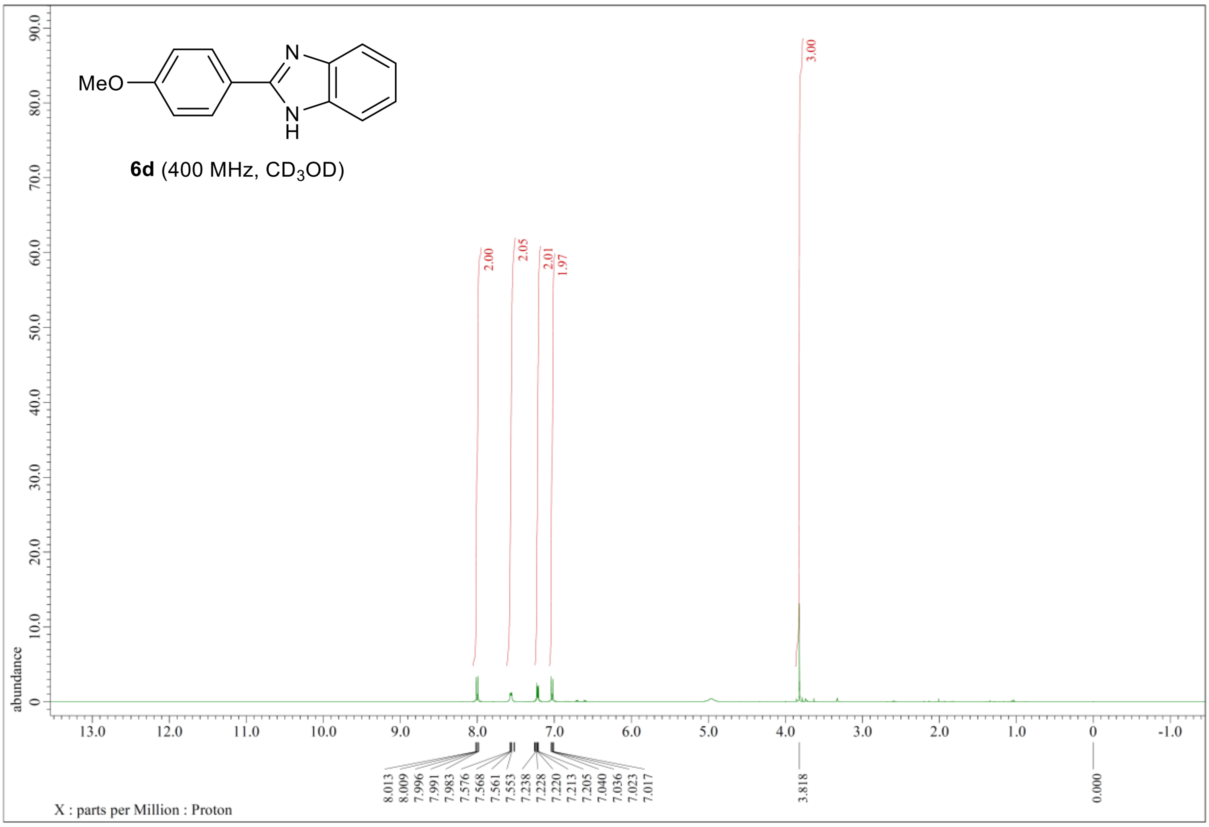

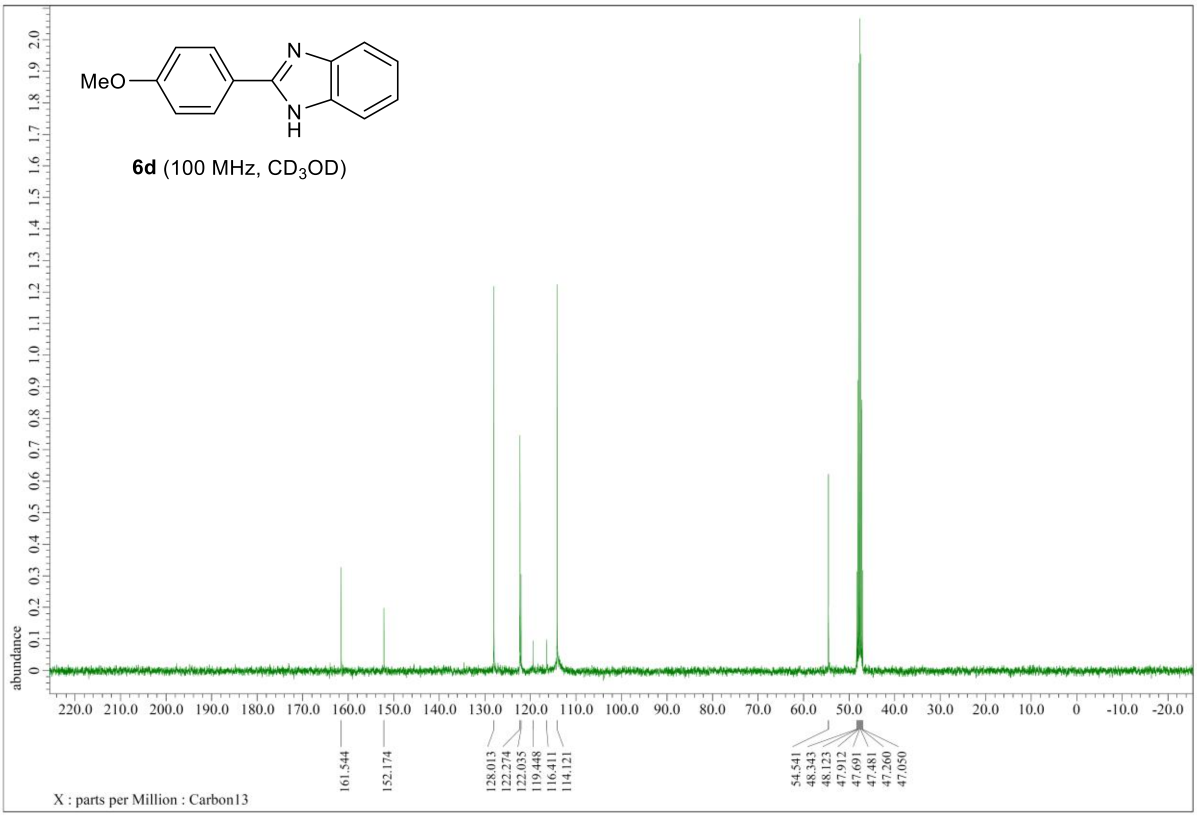

Figure S30: Copies of ^{1}H and $^{13}C\{^{1}H\}$ NMR spectra of compound **6e**

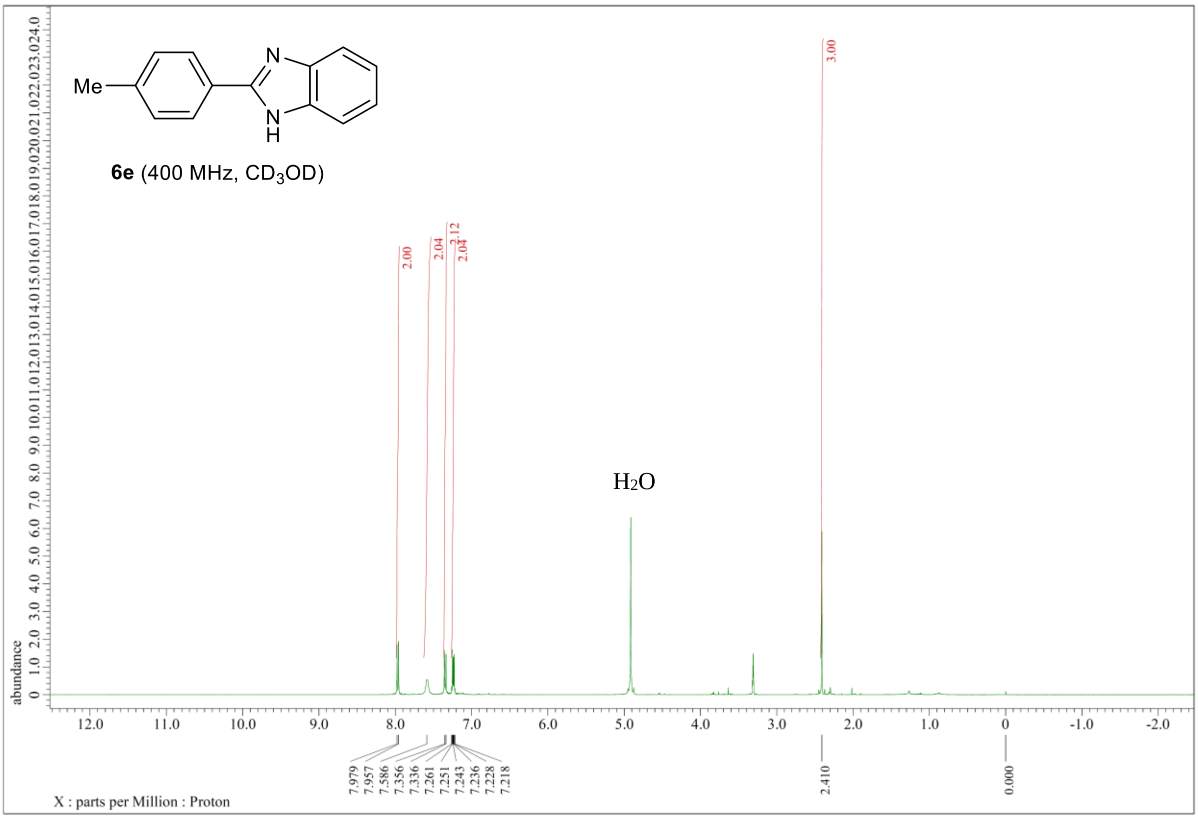

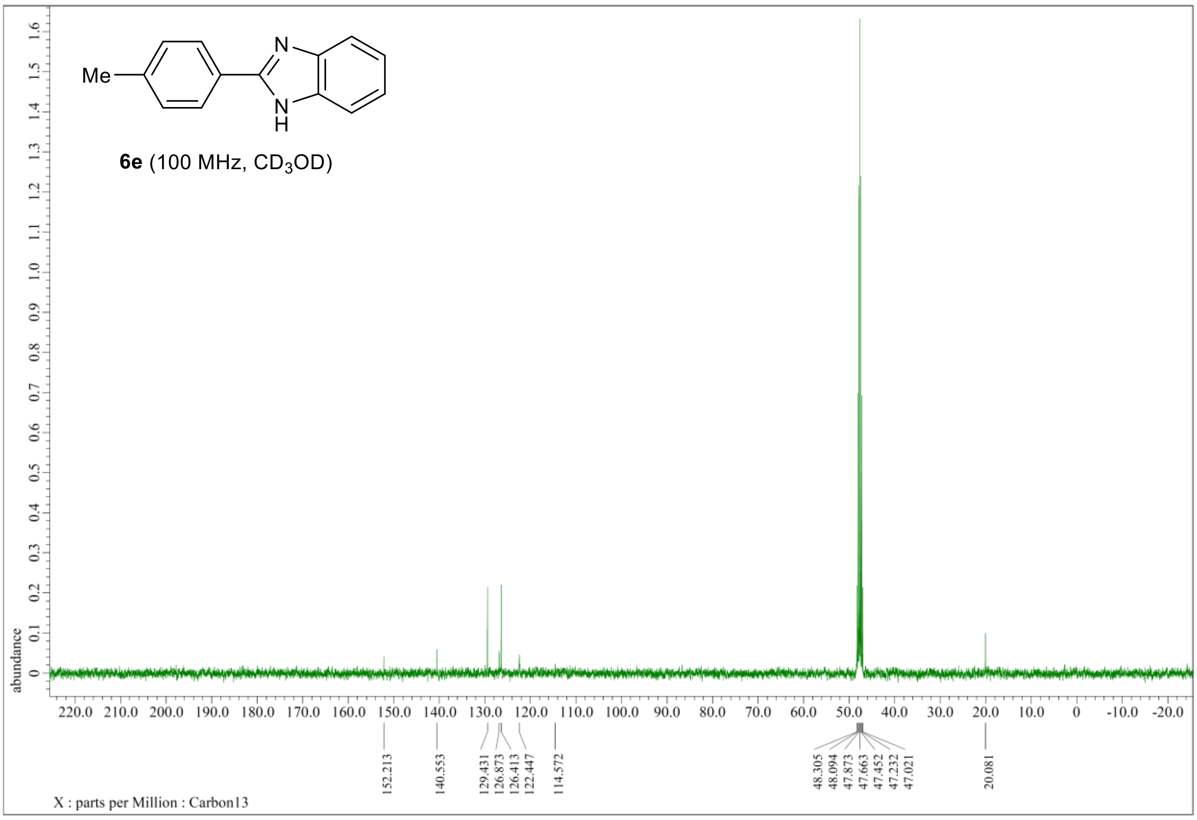

Figure S31: Copies of ^{1}H and $^{13}C\{^{1}H\}$ NMR spectra of compound **8**

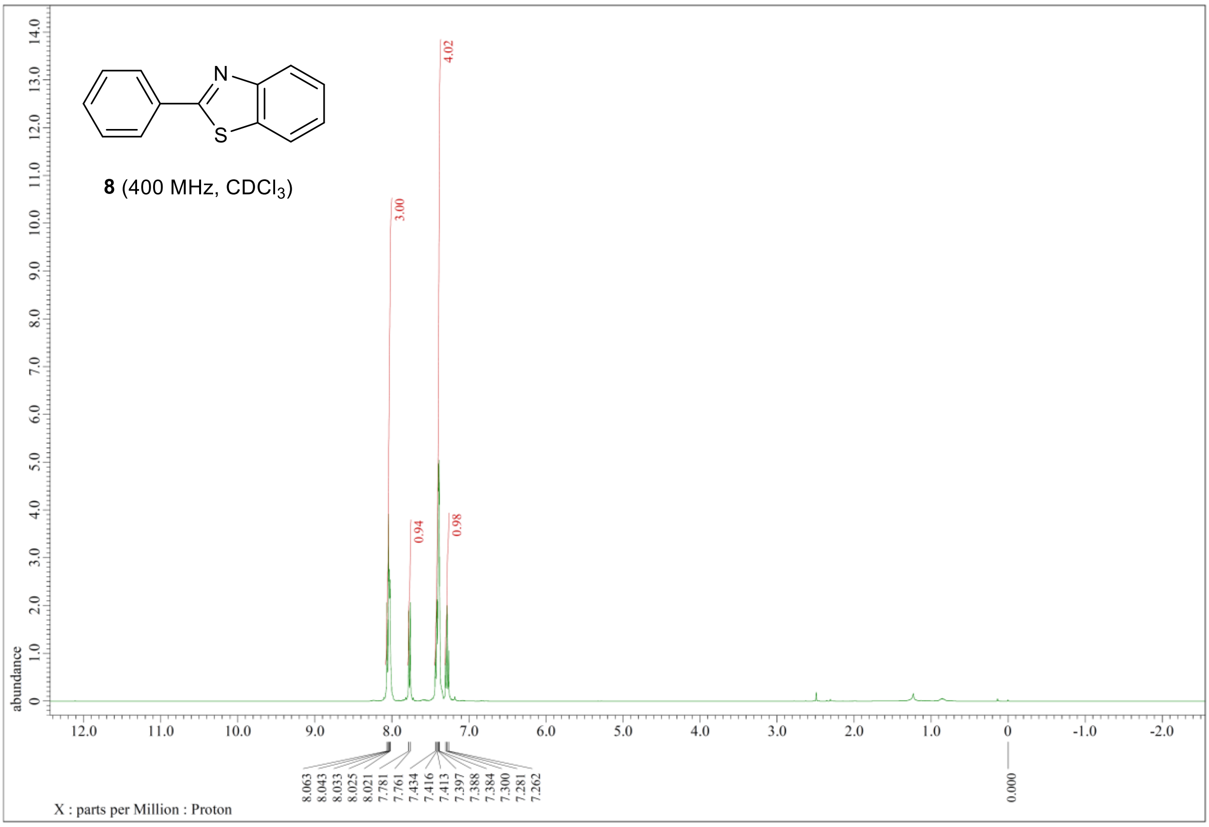

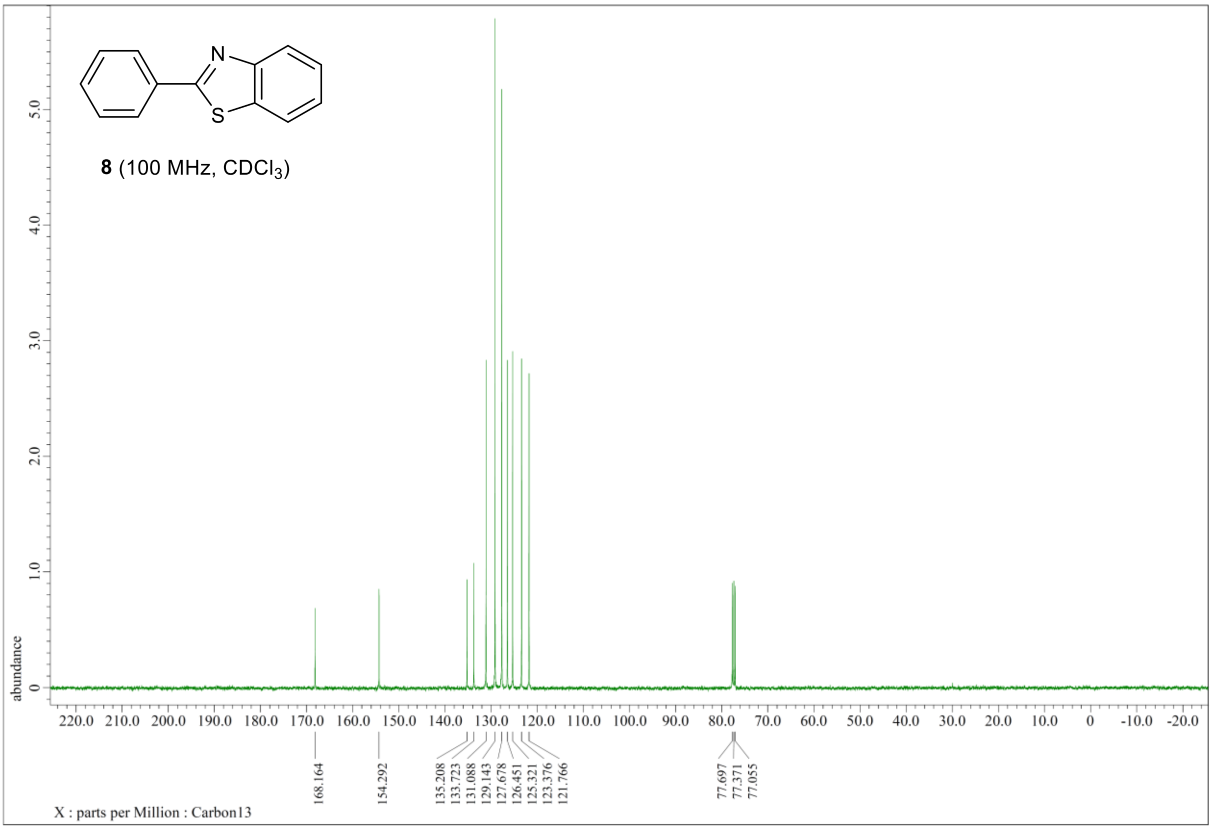

Figure S32: Copies of ^{1}H and ^{13}C{^{1}H} NMR spectra of compound **9**

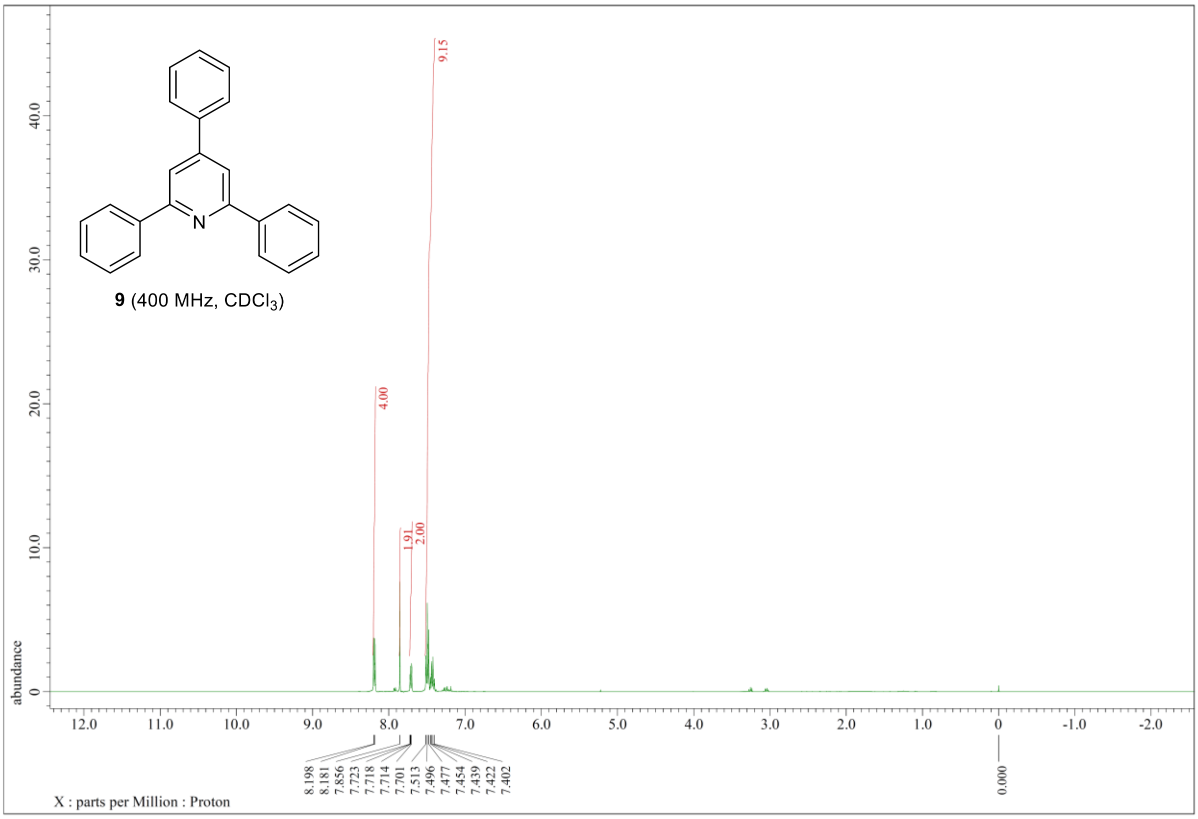

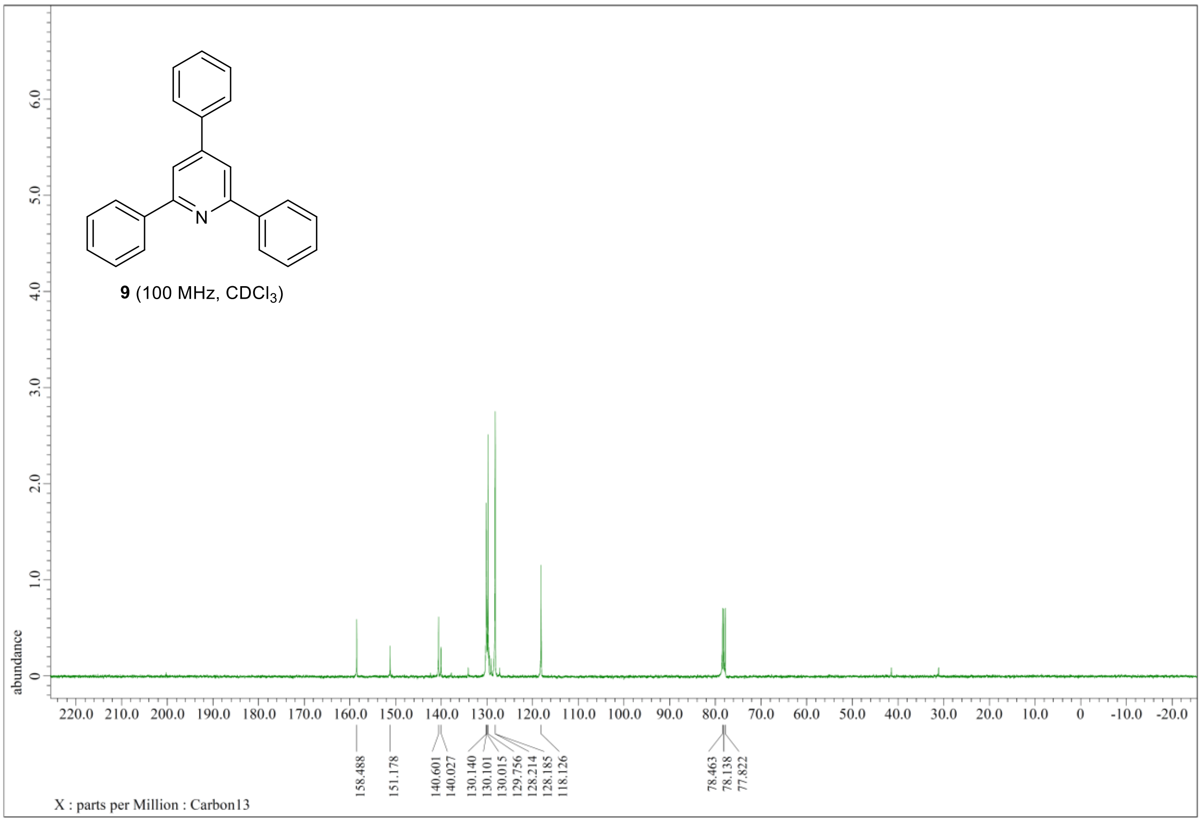

Supplement: Supplementary file 1 [file DataSheet1.pdf]
